# Development and Scale-Up of a New Sulfone-Based Bismacycle as a Universal Precursor for Bi(V)-Mediated Electrophilic Arylation

**DOI:** 10.1021/acs.oprd.3c00509

**Published:** 2024-02-07

**Authors:** Andrew Fox, Liam T. Ball

**Affiliations:** School of Chemistry, University of Nottingham, Nottingham NG7 2RD, U.K.

**Keywords:** bismuth, bismacycle, electrophilic arylation, lithiation, transmetalation, design of experiments

## Abstract

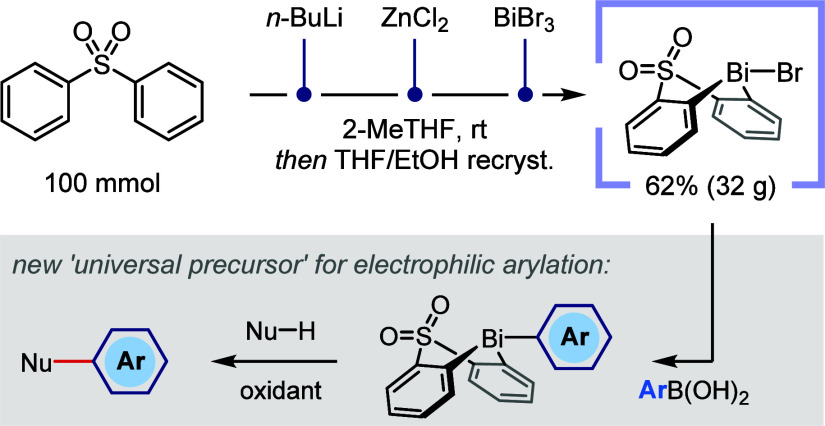

The scope and practical utility of bismuth(V)-mediated electrophilic arylation have been greatly improved by the recent development of user-friendly protocols based on modular bismacycle reagents. Here, we report the scalable synthesis of a new bench-stable bismacycle bromide and demonstrate that it can be used as a “universal precursor” in electrophilic arylation. Relative to established syntheses of related bismacycles, the new protocol benefits from improved step- and vessel-economy, reduced production time, and the complete elimination of cryogenic temperatures and undesirable solvents (Et_2_O and CH_2_Cl_2_). The synthesis is complemented by a robust, chromatography-free purification procedure that was developed by using design of experiments. We show that this process is highly reproducible at the 100 mmol scale, with two independent experiments giving 61 and 62% yields of isolated material. We anticipate that this efficient method for the synthesis of a new bismacycle precursor will expedite both (a) wider uptake of existing bismuth-mediated arylation methods by the synthetic community and (b) ongoing efforts to develop new bismuth-mediated transformations.

## Introduction

Electrophilic arylation strategies based on hypervalent bismuth(V) reagents^1−9^ are receiving renewed interest as powerful, and often complementary, alternatives to transition metal catalysis.^10−13^ This has been driven in part by the low cost and negligible toxicity of bismuth and its salts^14^ and in part by the unique disconnections that bismuth-mediated arylation enables. For example, Gagnon et al. have recently investigated the use of triarylbismuth(V) reagents for the C3-selective C–H arylation of indoles,^15^ whereas we demonstrated the utility of bismuthonium salts in a one-pot, three-component synthesis of quaternary amino acids.^16^ The atom economy, practicality, and generality of these, and the vast majority of previously reported,^17,18^ Bi-mediated methods are, however, limited by their reliance on homoleptic arylbismuth(V) reagents.

**Scheme 1:**
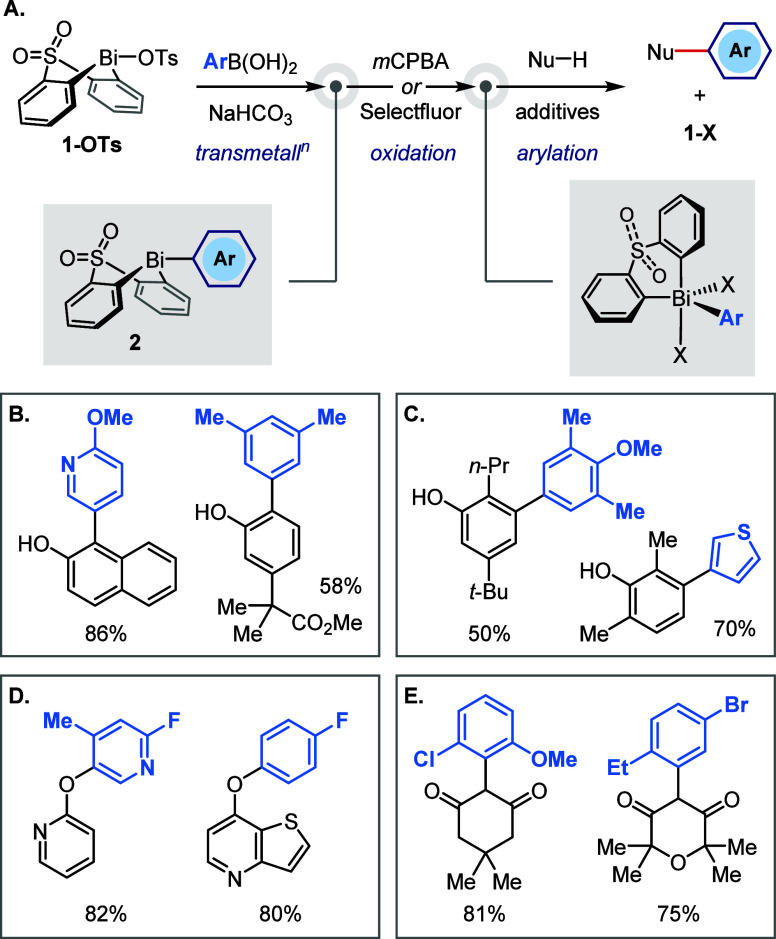
Bismacycle-Mediated Electrophilic Arylation^19,21−23^

In 2020,^19^ we reported the use of a sulfone-bridged bismacycle as a versatile platform for bismuth-mediated electrophilic arylation (Scheme 1A). Here, the two linked aryl rings represent an inert ligand scaffold that (1) modifies the reactivity and stability of the reagents relative to simple homoleptic triarylbismuthines and (2) improves atom economy by enabling selective transfer of solely the exocyclic aryl group. Excellent chemoselectivity and functional group compatibility are achieved by using the bismacycle in a stoichiometric, telescoped fashion that temporally separates the oxidant from the transmetallating agent and the nucleophilic partner. The practicality of this strategy relies on the ready accessibility of aryl bismacycles **2** from a general, bench-stable bismacycle precursor **1-OTs** via B-to-Bi transmetalation and benefits further from the ability to synthetically manipulate the aryl group following its installation at bismuth.^20^ Use of the bismacycle in a stoichiometric fashion is ultimately offset by its efficient recovery and recycling (as **1-X**; typically >90%).

We first demonstrated this strategy for the *ortho*-selective C–H arylation of phenols and naphthols (Scheme 1B),^19^ and subsequently extended it to the formal *meta*-selective C–H arylation of phenols as a concise route to densely functionalized biaryls featuring *contra*-electronic substitution patterns (Scheme 1C).^21^ Application to 2- and 4-pyridones results in chemoselective *O*-arylation (Scheme 1D),^22^ a reversal relative to the *N*-selectivity typically observed for pyridone nucleophiles.^24^ This represents a powerful alternative to the conventional S_N_Ar-disconnection of the aryloxypyridine motif, which we were able to showcase en route to four pharmaceutical and agrochemical active ingredients. Finally, the α-arylation of highly acidic cyclic diones enables the concise synthesis of herbicidal active ingredients that cannot be accessed using Pd- or Cu-catalysis, which have therefore traditionally been made with stoichiometric Pb (Scheme 1E).^23^ Concurrent with our first communication, Cornella et al. demonstrated the utility of similar bismacycles as catalysts for the conversion of arylboronic acids to industrially valuable aryl fluorides^25,26^ and subsequently extended this concept to the catalytic synthesis of aryl triflates.^27^

**Scheme 2:**
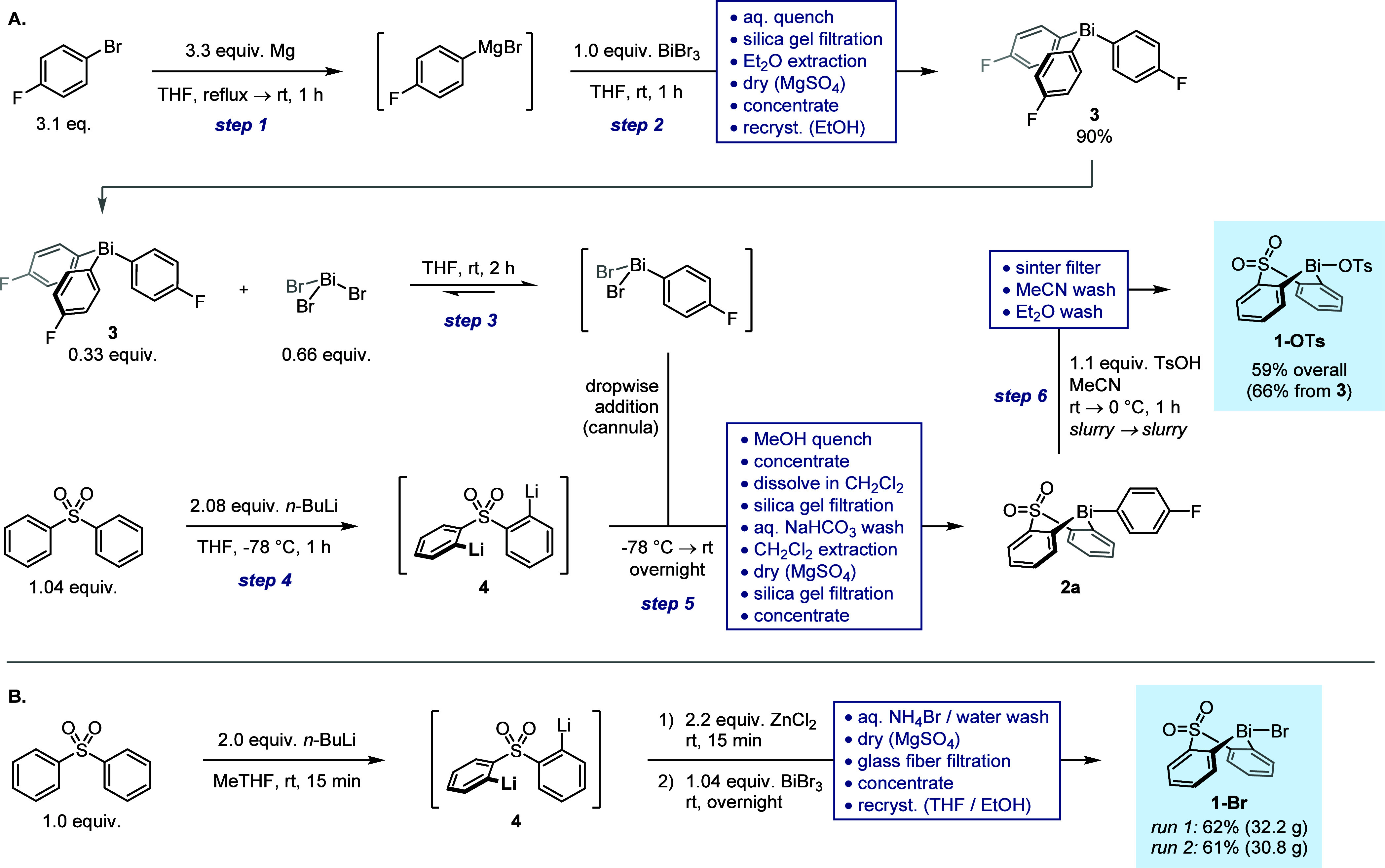
Established (A) and Improved (B) Processes for the Synthesis of Bismacycle Precursors **1-X**

Despite the recoverable nature of bismacycle **1**,^19,21−23^ access to significant quantities of the reagent is required for both large-scale and parallel small-scale studies. The current state-of-the art route to bismacycles of type **1**, a modification of Suzuki’s original procedure,^28^ is shown in Scheme 2A.^23^

Although the protocol illustrated in Scheme 2A does not involve any chromatographic purifications and employs only low-cost starting materials, it suffers from several major issues:

- The functional group manipulations in steps 1–3 and 6 introduce multiple operations that do not contribute directly to product formation, which ultimately result in poor atom/time efficiency.
- The Schlenk equilibrium^29^ to form an arylbismuth dibromide (step 3) is imperfect, and its dynamic nature often results in the presence of triarylbismuthine **3** as an impurity that must be removed from aryl bismacycle **2a**.
- The overall sequence exhibits a variable and unpredictable impurity profile (*vide infra*) that prevents development of a general, robust purification protocol.
- The final operation is a slurry-to-slurry process (step 6), which makes monitoring of the reaction progress challenging.
- The poor solubility of the final bismacycle tosylate **1-OTs** limits options for additional purification by recrystallization or chromatography if impurities persist after the standard precipitation/filtration.

The use of cryogenic conditions (Scheme 2A; steps 4 and 5), multiple unit operations, and solvents with poor sustainability/safety profiles (*e.g*., Et_2_O and CH_2_Cl_2_) present additional challenges to scale-up of this process. Furthermore, in our experience, the protocol exhibits poor reproducibility, presumably due to the practical challenges outlined above and the sensitivity of the key Schlenk equilibrium (step 3) to the quality of the BiBr_3_. Given the growing interest in and potential industrial utility of bismuth-mediated arylations, there is thus a need for an efficient, reliable, and more sustainable route to bismacycles of the type **1-X**.

Here, we report a scalable and reproducible approach to bismacycle bromide **1-Br** that allows >30 g to be made in a 24 h period without chromatographic purification (Scheme 2B). This new process avoids triarylbismuth intermediates and cryogenic conditions and has an improved process mass intensity (PMI). We demonstrate that bismacycle bromide **1-Br** is stable to storage and performs equivalently to bismacycle tosylate **1-OTs** in downstream chemistry, and therefore that it can be used as a drop-in replacement to enable the application of existing, and the discovery of new, Bi(V)-mediated arylations.

## Results and Discussion

In redesigning the synthesis of bismacycles of type **1-X**, a key aim was to avoid the intermediacy of aryl bismacycles **2**: this modification would not only reduce step-count and unit operations but would also improve atom efficiency and eliminate frequent sources of impurities and irreproducibility. We also sought to (a) avoid the use of cryogenic temperatures, (b) employ more sustainable solvents, and (c) develop a more robust purification protocol.

### Investigation of Bismuth Sources

We initiated our study by assessing different bismuth sources, which not only determines the identity of the bismacycle (i.e., “X” in **1-X**) but also has implications for process practicality and cost. BiI_3_ was immediately ruled out on the basis of its cost (£2030/mol),^30^ poor mass economy, and the environmental toxicity and disposal issues associated with iodide waste. Where BiCl_3_ is both significantly cheaper (£504/mol) and readily available, the salt is hygroscopic and hydrates rapidly under ambient air prior to hydrolysis to polymeric BiOCl.^31^ In contrast, BiBr_3_ is a financial compromise (£1040/mol) but is noticeably easier to weigh under ambient conditions. Furthermore, initial investigations using authentic samples of the bismacycle chloride **1-Cl** and bromide **1-Br** indicated that the former is appreciably hygroscopic, whereas the latter proved to be easy to handle. For example, solid samples of **1-Br** were unchanged after having been stored on the bench under ambient conditions for over 2 months, and a DMSO/water solution of **1-Br** showed less than *ca* 1% decomposition after 1 day at room temperature.

**Scheme 3:**
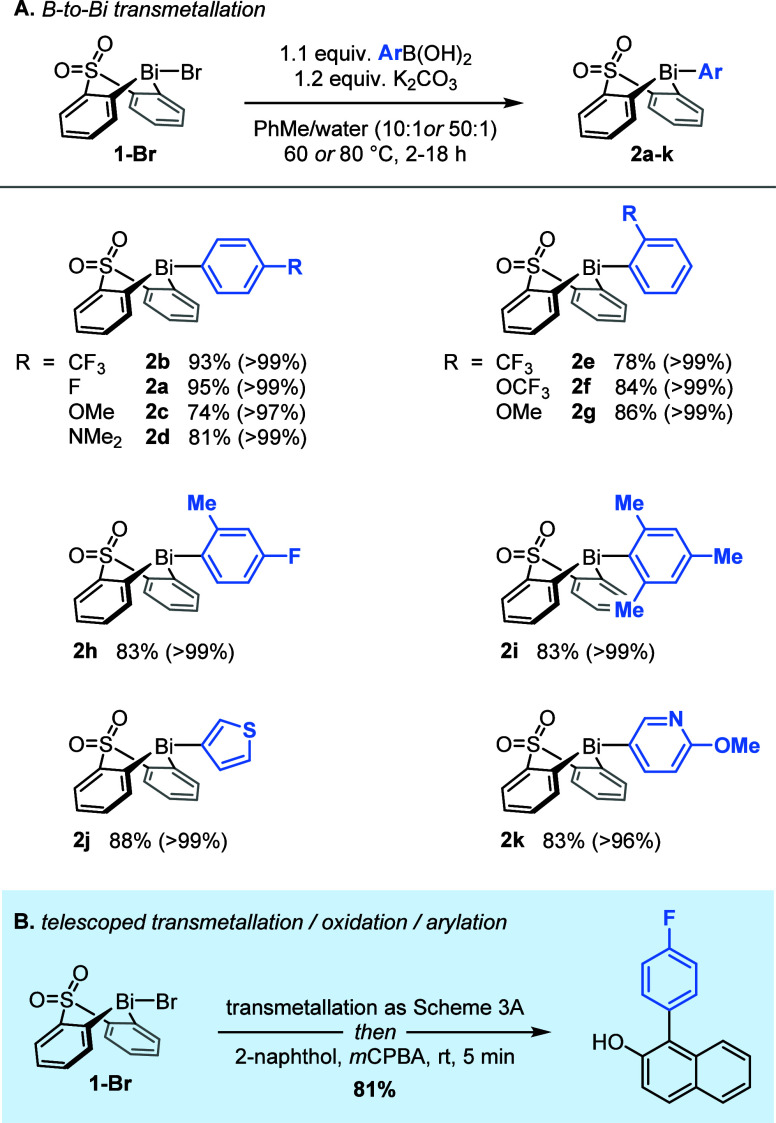
B-to-Bi Transmetallation and Telescoped Arylation Using Bismacycle **1-Br** Reactions performed on a 0.5 mmol scale (see Supporting Information for detailed conditions). Yields in Scheme 3A refer to material isolated following purification; values in parentheses refer to conversion as determined by ^1^H NMR spectroscopic analysis. Yield in Scheme 3B is determined by ^19^F NMR spectroscopic analysis vs internal standard.

To further confirm the suitability of the bismacycle bromide **1-Br** as an alternative to tosylate **1-OTs**, we investigated its competence in B-to-Bi transmetalation and hence its compatibility with established downstream applications. These studies indicated that facile transmetalation of sterically and electronically diverse aryl moieties could be achieved in excellent yields under conditions equivalent to those reported for **1-OTs** (Scheme 3A) and that telescoped arylation of 2-naphthol can be achieved without modification of the literature procedure (Scheme 3B, see the Supporting Information for additional examples).^19,23^ On this basis, we selected BiBr_3_ for use in further studies.

### Optimization of Bismacycle **1-Br** Synthesis

The most efficient approach to bismacycle **1-Br** is–at least on paper–via the direct reaction of a dimetalated diphenyl sulfone with the simple BiBr_3_ salt (Scheme 2B). However, this approach is not precedented for synthesis of the sulfone-bridged bismacycle scaffold, so we next sought to identify a suitable organometallic nucleophile that could yield **1-Br** directly.

**Scheme 4:**
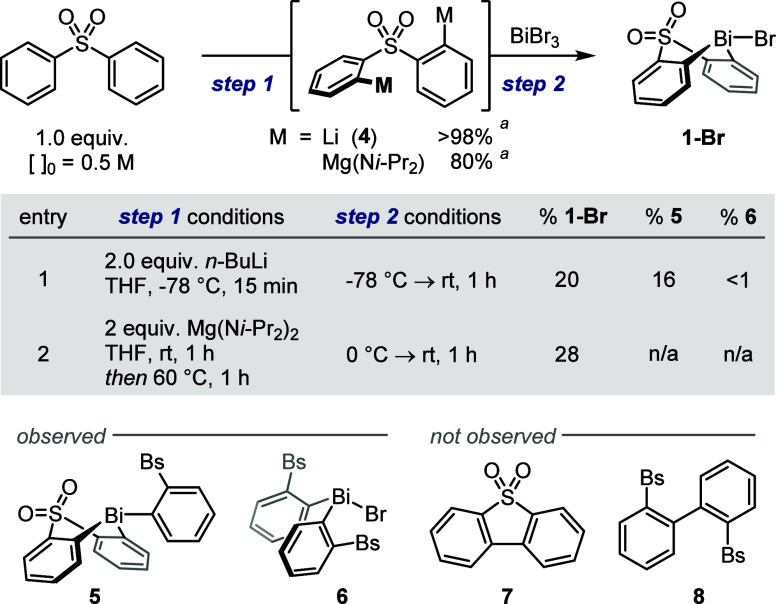
Direct Metalation/Bismuthation of Diphenyl Sulfone Conversion determined by ^1^H NMR spectroscopic analysis following D_2_O quench. Yields in entry 1 were determined by ^1^H NMR spectroscopic analysis vs internal standard (1,3,5-trimethoxybenzene); yield in entry 2 refers to material isolated following purification. n/a, not detected by ^1^H NMR spectroscopic analysis of the crude reaction mixture; Bs, benzenesulfonyl.

While lithiation of diphenyl sulfone with *n*-butyllithium is facile, addition of the resulting dilithiodiphenyl sulfone **4** to BiBr_3_ afforded less than 20% of the desired bismacycle **1-Br** as part of a complex mixture containing side-products **5** and **6** (Scheme 4, entry 1). The former is presumably formed via addition of highly nucleophilic dilithiodiphenyl sulfone **4** to the initially formed **1-Br**, followed by protonation of a pendant aryllithium or an intermediate 10-Bi-4 “ate” complex^32−34^ during reaction quench. As determined by comparison to authentic samples, the likely products of reductive coupling (**7** and **8**) were not formed in observable quantities.

The unsuitability of dilithiodiphenyl sulfone **4** for the direct synthesis of **1-Br** is comparable to the reported poor performance of organolithium reagents in the preparation of biphenylene-based bismacycles directly from bismuth trihalides.^35^ In contrast, Grignard reagents are used routinely as nucleophiles for the addition to BiX_3_ salts (*cf*. Scheme 2A, step 2), prompting an investigation into the direct synthesis of the softer dimagnesiated diphenyl sulfone (Scheme 4, entry 2). As determined by D_2_O quenching, metalation of diphenyl sulfone with two equivalents of magnesium bis(di*iso*propylamide) did not proceed to completion; subsequent addition of BiBr_3_ afforded bismacycle **1-Br** in only 28% isolated yield and led to the formation of appreciable amounts of black precipitate (presumably Bi(0), which is known to form from Bi(III) amides upon their exposure to light and/or heat^36−38^).

Given the ease with which dilithiodiphenyl sulfone **4** can be prepared, we investigated a two-step procedure in which C–H lithiation is followed by transmetalation onto either a copper(I) or a zinc(II) salt. The resulting organometallic reagents are less reducing, and both classes are known to react cleanly with bismuth(III) salts.^29,33^ At this stage, we also replaced THF with MeTHF in order to improve sustainability and facilitate subsequent aqueous work-ups.^39^ To account for the lower solubility of both diphenyl sulfone and bismacycle **1-Br** in MeTHF, the reaction concentration was adjusted from 0.5 to 0.25 M. Under these conditions, dilithiation of diphenyl sulfone was complete in less than 1 min at room temperature, and the resulting organolithium reagent **4** proved stable in solution for at least 2.5 h at room temperature.

Addition of 2.2 equiv of CuCl to dilithiodiphenyl sulfone **4** in MeTHF at room temperature gave a thick slurry, which reacted with BiBr_3_ to give **1-Br** in 66% spectroscopic yield (Table 1, entry 1). Using only 1.2 equiv of CuCl afforded a similarly impractical slurry and gave **1-Br** in reduced yield and purity (entry 2). In contrast, the inverse addition of **4** to a suspension of 2.2 equiv of CuCl in MeTHF afforded a soluble organocopper species that could easily be transferred into a solution of BiBr_3_, giving **1-Br** in 66% yield (entry 3). The use of CuBr in place of CuCl gave similar results (Table 1, entry 4), whereas CuI afforded the bismacycle in 28% yield as a 7:3 mixture of its bromide and iodide salts (entry 5). While **1-I** could be converted to **1-Br** by washing with a saturated aqueous solution of LiBr, the overall yield remained modest. Repeating the CuCl reaction outlined in entry 3 on a 10 mmol scale resulted in an improved spectroscopic yield and purity (entry 6). However, the subsequent workup required filtration through diatomaceous earth to remove fine particulates, and the resulting filtrate retained a faint green color consistent with copper contamination. This latter observation was deemed particularly problematic due to the established reactivity of both Bi(III) and Bi(V) reagents toward copper salts.^17,18^

**Table 1:**
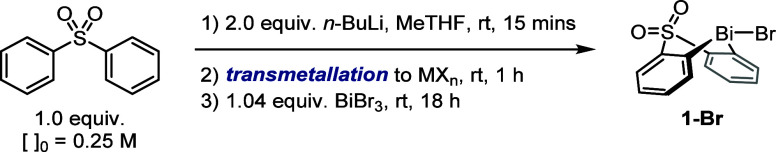
Synthesis of **1-Br** via Transmetallation of **4** to Cu or Zn Salts

		**% 1-Br**^a^	
**entry**	transmetalation **conditions**	**yield**	**purity**
1	2.2 equiv solid CuCl added to **4**	66	87
2	1.2 equiv solid CuCl added to **4**	50	81
3	**4** added to 2.2 equiv CuCl in MeTHF	66	82
4	**4** added to 2.2 equiv CuBr in MeTHF	72	88
5^b^	**4** added to 2.2 equiv CuI in MeTHF	28	60
6^c^	**4** added to 2.2 equiv CuCl in MeTHF	84	94
7^c^	2.2 equiv ZnCl_2_ in MeTHF added to **4**	76	81
8^c^	2.2 equiv ZnCl_2_ in MeTHF added to **4**	72	76

a Yields and w/w purity were determined by ^1^H NMR spectroscopic analysis vs internal standard (1,3,5-trimethoxybenzene).

b Bismacycle formed as a 7:3 mixture of **1-Br** and **1-I**.

c Reaction was performed using 10 mmol diphenyl sulfone. All metal salts were dried prior to use; see the Supporting Information for details.

Given the environmental toxicity of copper salts and the potential for metal contamination of **1-Br**, efforts were instead focused on the use of organozinc intermediates. Bismacycle **1-Br** was formed in reproducibly high yields and purities when dilithiodiphenyl sulfone **4** was transmetalated to a solution of 2.2 equiv of ZnCl_2_ before addition to BiBr_3_ (Table 1, entry 7). Both the lithiation and the Li-to-Zn transmetalation steps were observed to be accompanied by moderate exotherms, with reaction temperatures increasing by 14 and 11 °C (respectively) on a 10 mmol scale. These temperature increases were limited to <2 °C in subsequent repetitions on a 100 mmol scale (*vide infra*) simply by controlling the rate of addition and by applying external cooling with an ambient temperature water bath. Following addition of the organozinc intermediate to BiBr_3_, an aqueous quench afforded a 9.5:1 mixture of bismacycle bromide **1-Br** and the corresponding chloride **1-Cl**. The latter was converted to the desired bromide by washing with a 1 M aqueous ammonium bromide solution. The major residual impurities were determined to be **5** (*ca* 6% w/w) and **6** (9% w/w). Attempts to convert the former to **1-Br** by selective protonolysis of the exocyclic Bi–C bond with HBr was accompanied by competing decomposition of the bismacycle scaffold and was therefore not explored further.

### Optimization of Purification Protocol

Having developed a reproducible synthesis procedure, we sought to identify robust crystallization conditions that would allow the isolation of **1-Br** in both high yield and high purity. Initial studies identified THF and MeTHF as able to dissolve both bismacycle **1-Br** and the major process impurities **5** and **6** (Table 2, entries 1 and 2). TBME, EtOH, and MeOH showed promise as antisolvents (entries 3–5), selectively dissolving a large proportion of impurities **5** and/or **6**, and only single digit percentages of desired product **1-Br** (<6%). In contrast, cyclohexane and moderately polar solvents proved inappropriate, either partially dissolving **1-Br** or failing to efficiently solubilize **5** or **6** (entries 6–11).

**Table 2:**
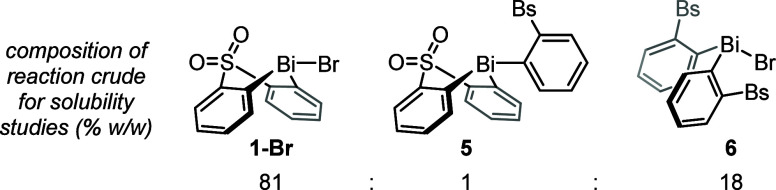
Solubility of **1-Br** and Side-Products **5** and **6**^a^

		**% dissolution**		
**entry**	**solvent**	**1-Br**	**5**	**6**
1	MeTHF	65	>99	78
2	THF	64	>99	75
3	TBME	6	38	71
4	EtOH	3	21	70
5	MeOH	3	13	68
6	cyclohexane	<1	<1	17
7	PhMe	26	75	71
8	*i*-PrOAc	17	53	74
9	EtOAc	23	96	70
10	MeCN	14	82	74
11	*i*-PrOH	1	16	41

a Conditions: crude material (w/w composition: **1-Br**, 81%; **5**, 6%; **6**, 9%) in 10 volumes solvent stirred for 2 h at rt, 800 rpm. Solubilities were determined by analysis of the filtrate by ^1^H NMR spectroscopy vs internal standard (1,3,5-trimethoxybenzene).

The data presented in Table 2 were used as the basis for development of a cooled antisolvent crystallization. To this end, dissolving the crude material in a minimum volume of MeTHF (8 volumes, where 1 volume = 1 mL of solvent per gram of material) at 50 °C followed by addition of an equivalent volume of EtOH, MTBE, or cyclohexane and cooling to room temperature gave modest recovery of pure crystalline **1-Br** (Table 3, entries 1–3). In contrast, the crude material could be dissolved in only three volumes of THF at 50 °C, which represents a preferable economy of volume, and pure **1-Br** was recovered in 51% yield following addition of three volumes of EtOH (entry 4).

**Table 3:**
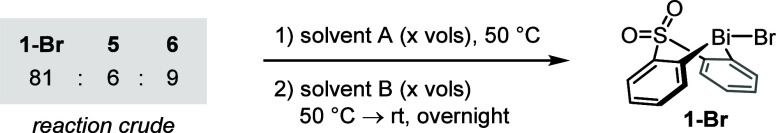
Assessment of Binary Solvent Systems for the Crystallization of **1-Br**^a^

	**solvents**			
**entry**	**A**	**B**	**X**	**% 1-Br**
1	MeTHF	EtOH	8	39 (6)
2	MeTHF	MTBE	8	26
3	MeTHF	CyH	8	32
4	THF	EtOH	3	41 (10)

a Conditions: crude material (w/w composition: **1-Br**, 81%; **5**, 6%; **6**, 9%) dissolved in solvent A at 50 °C; solvent B was added, and the reaction was cooled to room temperature overnight. Yields refer to pure material isolated by filtration; values in parentheses refer to additional pure material isolated as a second crop.

**Figure 1:**
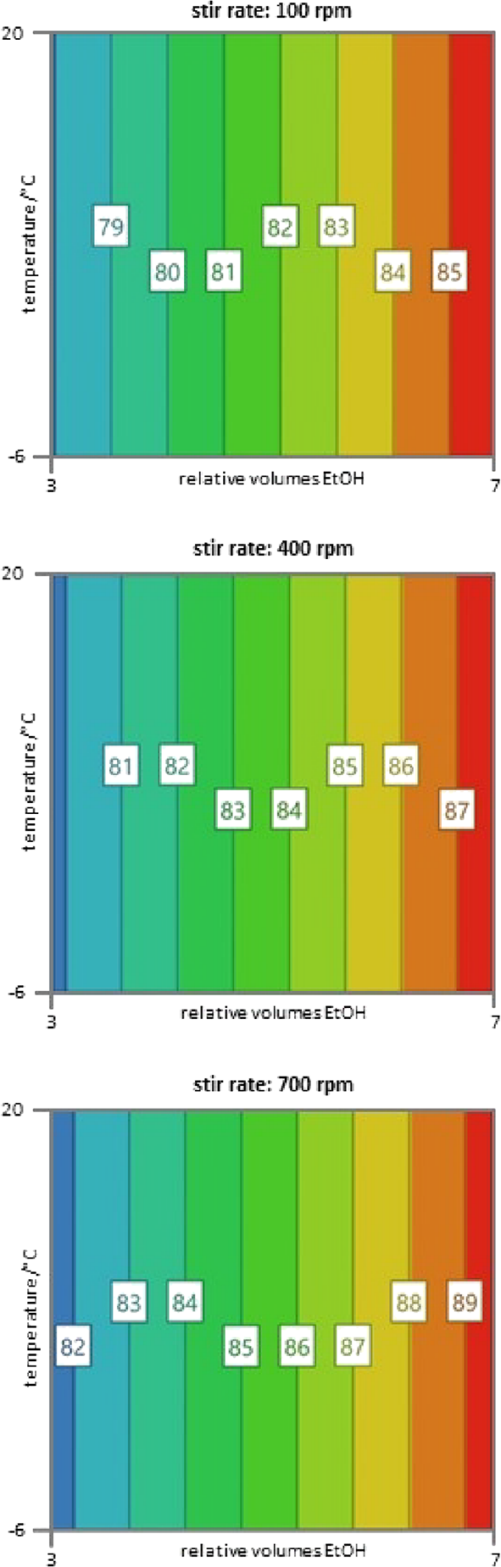
Crystallization Design of Experiments contour plots illustrating the effect of stirring rate, temperature, and relative volumes of EtOH on the recovery of **1-Br** from crude material with w/w composition: **1-Br**, 94%; **5**, 4%; **6**, <1%. Each plot corresponds to a different stirring rate (top, 100 rpm; middle, 400 rpm; bottom, 700 rpm); *x*-axes correspond to the relative volume of EtOH added to a solution of crude **1-Br** in three volumes of THF at 50 °C; *y*-axes show the final hold temperature of the crystallization (cooling rate from 50 °C: 1 °C min^–1^; hold duration: 20 h). Numerical values in white boxes refer to the percentage of **1-Br** precipitated, as determined by the ^1^H NMR spectroscopic analysis of the crystallization liquor following filtration. See the Supporting Information for details of the experimental procedure, raw data, and analysis of the design.

A more comprehensive assessment of crystallization from THF/EtOH was performed using design of experiments (full factorial, two levels, three factors; three center points).^40^ Keeping the initial volume and temperature of THF constant (three volumes, 50 °C) and considering as factors the stirring rate, the final hold temperature, and the relative volume of EtOH gave a remarkably flat response surface (Figure 1). The main effect was a positive dependence on the relative volume of EtOH, and no interactions were observed between factors. This insight enabled a marginal increase in yield and gave us confidence in the robustness of the crystallization, and hence its suitability for application at larger scales.

### Process Scale-Up

**Scheme 5:**
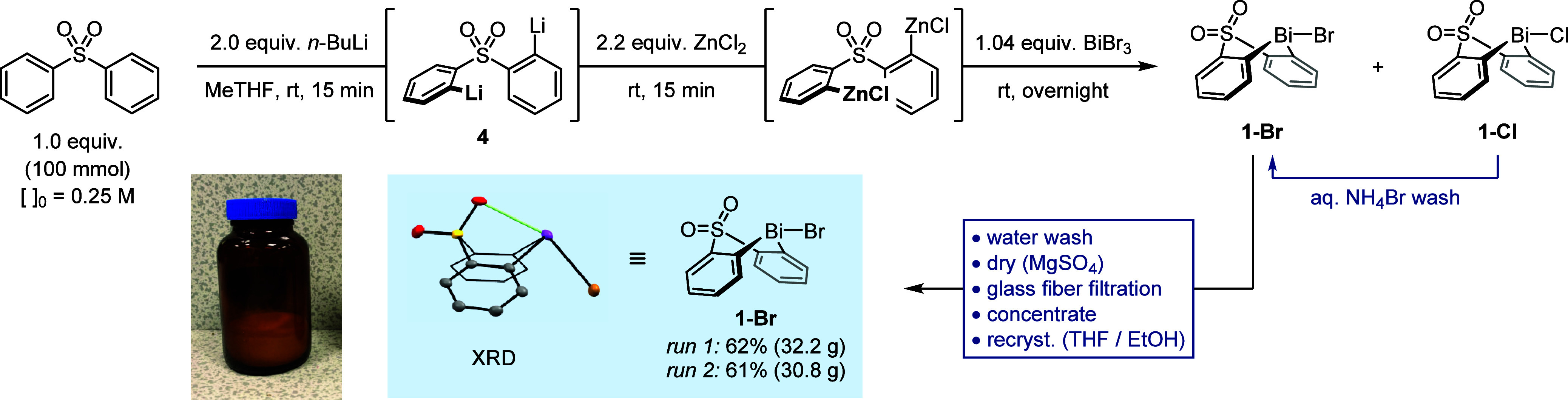
Scaled-Up Synthesis of Bismacycle Bromide **1-Br** Yields refer to three combined crops of material isolated following purification. ORTEP image of **1-Br**: H atoms omitted for clarity; thermal ellipsoids shown at 50% probability.

Performing the optimized synthesis procedure twice on a 100 mmol scale gave bismacycle **1-Br** in isolated yields of 61 and 62% (>30 g per run; Scheme 5). The average yield of **1-Br** is comparable to the yield of **1-OTs** (59% over two steps), and the new process benefits from greatly improved reproducibility and a significantly reduced production time. With a PMI^41^ of 95 (vs 108 for **1-OTs**), the synthesis of **1-Br** also has an improved sustainability profile: the total solvent usage is lowered from 33 to 26 mL/mmol, and the use of hazardous solvents (Et_2_O and CH_2_Cl_2_) is entirely eliminated and replaced with greener alternatives (MeTHF and EtOH). Furthermore, the removal of solid particulates by aqueous washing and filtration through glass fiber filter paper eliminates the need for a filtration aid, such as silica gel or diatomaceous earth, which had contributed 6.6 g/mmol of solid waste to the synthesis of **1-OTs**.

## Conclusions

We have developed a scalable route to bench-stable bismacycle bromide **1-Br**, which we demonstrate serves as a new “universal precursor” to aryl bismacycles of type **2**. The optimized synthesis procedure is highly reproducible on a 100 mmol scale, uses only commercial reagents, and avoids both cryogenic conditions and chromatographic purification. High selectivity (*ca.* 95 mol %) for bismacycle **1-Br** is achieved by transmetalation of the first-formed dilithiodiphenyl sulfone **4** to ZnCl_2_ prior to bismuthation, with final purification achieved via a robust cooled antisolvent crystallization. Unlike the preparation of analogous bismacycles (*e.g*., **1-OTs**), **1-Br** is accessed without the intermediacy of triarylbismuth reagents. Step-count, production time, and reagent usage are therefore reduced, which, in combination with the elimination of hazardous solvents (Et_2_O and CH_2_Cl_2_), ultimately results in reduced PMI. Therefore, given that bismacycle **1-Br** can be used as a replacement for **1-OTs** in existing electrophilic arylation methods, we anticipate that the convenience and scalability of its synthesis will expedite the uptake of established, and the development of new, bismuth-mediated transformations.

## Experimental Section

### 10-Bromo-10*H*-dibenzo[*b*,*e*][1,4]thiabismine 5,5-dioxide (**1-Br**)

#### Flask 1

ZnCl_2_ (31.1 g, 220 mmol, 2.2 equiv) was added to a flame-dried 300 mL Schlenk flask and dried under vacuum for 3 h at 150 °C. After cooling to room temperature and backfilling with anhydrous dinitrogen, anhydrous MeTHF (200 mL) was added, and the mixture was stirred until a homogeneous solution was formed.

#### Flask 2

A flame-dried 1 L three-necked round bottomed flask containing diphenyl sulfone (22.5 g, 100 mmol, 1.0 equiv) and fitted with a thermometer was evacuated and backfilled three times with anhydrous dinitrogen. Anhydrous MeTHF (400 mL) was added, and the mixture was stirred until a homogeneous solution was formed. The stirred solution was immersed in a bath of ambient-temperature water, and then *n*-butyllithium (2.47 M in hexanes; 81.0 mL, 200 mmol, 2.0 equiv) was added over 60 min so that the internal temperature did not rise by more than 2 °C. The resulting orange/brown suspension was stirred for 15 min, and then the ZnCl_2_ solution (from *flask 1*) was added dropwise via cannula over 25 min so that the internal temperature did not rise by more than 2 °C. The resulting yellow solution was stirred for a further 15 min.

#### Flask 3

A flame-dried 2 L three-necked round bottomed flask containing BiBr_3_ (46.7 g, 104 mmol, 1.04 equiv) was evacuated and backfilled thrice with anhydrous dinitrogen. Anhydrous MeTHF (400 mL) was added, and the mixture was stirred until a homogeneous solution was formed. The stirred solution was then immersed in a bath of ambient-temperature water, and the organozinc solution (from flask 2) was added dropwise via a cannula over 35 min. The resulting pale-yellow suspension was stirred overnight, and then the mixture was quenched with aq. NH_4_Br (1 M, 100 mL) and diluted with water (200 mL). The organic phase was separated and washed with aq NH_4_Br (1 M; 3 × 250 mL) and water (3 × 250 mL), dried with MgSO_4_, and then filtered through glass fiber paper. The filter cake was rinsed with EtOAc (50 mL). The filtrate was concentrated *in vacuo* to afford a pale-yellow powder (43 g), then THF (130 mL, three volumes) was added, and the mixture was stirred at 50 °C to give a homogeneous solution. EtOH (300 mL, 7 volumes; preheated to 50 °C) was added, and the mixture was allowed to cool to room temperature over *ca.* 30 min with stirring and then cooled in an ice bath and held at 0 °C for 1 h. The solid was collected on a sintered glass frit by vacuum filtration, and the filter cake was washed with cyclohexane (40 mL) and dried under a flow of air to afford the first crop of bismacycle **1-Br** as a colorless solid (25.3 g, 50 mmol, 50%). The filtrate was concentrated *in vacuo*, and the resulting yellow solid was resubjected to the crystallization procedure to afford the second (3.1 g, 6.2 mmol, 6%) and third (3.9 g, 7.6 mmol, 7%) crops of bismacycle **1-Br**, which were indistinguishable from the first crop by ^1^H and ^13^C{^1^H} NMR spectroscopy. The total yield of **1-Br** over three crops was 62% (32.2 g, 63.8 mmol; 94.5% w/w purity by qNMR). Repetition of the synthesis on the same scale afforded **1-Br** in 61% isolated yield (30.8 g, 61.0 mmol; 95.7% w/w purity by qNMR).

^1^H NMR (400 MHz, CDCl_3_): δ_H_ 8.98 (dd, *J* = 7.4, 1.1 Hz, 2H), 8.33 (dd, *J* = 7.7, 1.2 Hz, 2H), 7.70 (app. td, *J* = 7.5, 1.3 Hz, 2H), 7.49 (app. td, *J* = 7.6, 1.1 Hz, 2H).

^13^C{^1^H} NMR (101 MHz, CDCl_3_): δ_C_ 173.6, 140.4, 137.5, 135.9, 128.9, 128.3.

υ_max_(ATR)/cm^–1^: 3041, 1557, 1438, 1423, 1288, 1251, 1130, 1106, 1095, 1084, 1063, 1022, 1007, 951, 872, 756, 736, 711, 696, 636, 585, 559, 505, 462, 417.

HRMS calcd. for C_12_H_8_BiO_2_S^+^: 425.0044 [M-Br]^+^; found (ESI^+^): 425.0036.

m.p./°C: 241–243.

## Supporting Information Available

Experimental procedures; spectra; and optimization data (PDF)
X-ray crystallographic data (CIF)

## Supplementary Material

op3c00509_si_001.pdf

op3c00509_si_002.cif
