# Supplementary material for: Development and Scale-Up of a New Sulfone-Based Bismacycle as a Universal Precursor for Bi(V)-Mediated Electrophilic Arylation
Source: Org Process Res Dev. 2024 Feb 7;28(2):632–9. doi: 10.1021/acs.oprd.3c00509 (PMC10877598; doi:10.1021/acs.oprd.3c00509)

Supporting Information

Development and Scale-Up of a New Sulfone-Based Bismacycle as a Universal Precursor for Bi(V)-Mediated Electrophilic Arylation

Andrew Fox, Liam. T. Ball*

School of Chemistry, University of Nottingham, Nottingham NG7 2RD, U.K.

corresponding author: liam.ball@nottingham.ac.uk

Table of Contents

1 General Information ... 2
2 B-to-Bi Transmetallation and Electrophilic Arylation (manuscript Scheme 3) ... 4
2.1 General Procedure for B-to-Bi Transmetallation (GP1) ... 4
2.2 Characterization Data for Aryl Bismacycle Products ... 5
2.3 Procedures for Electrophilic Arylation ... 16
3 Additional Synthesis Procedures for Bismacycle Halides ... 18
3.1 10-Chloro-10*H*-dibenzo[*b*,*e*][1,4]thiabismine 5,5-dioxide **1-Cl** ... 18
3.2 10-Bromo-10*H*-dibenzo[*b*,*e*][1,4]thiabismine 5,5-dioxide **1-Br** ... 19
3.2.1 **1-Br** *via* Magnesiation (manuscript Scheme 4, entry 2) ... 19
3.2.2 **1-Br** *via* Transmetallation to CuCl (manuscript Table 1, entry 6) ... 19
3.2.3 **1-Br** *via* Transmetallation to $ZnCl_2$ (manuscript Table 1, entry 7) ... 20
4 Identification of Process Impurities ... 22
4.1 10-(2-(Phenylsulfonyl)phenyl)-10*H*-dibenzo[*b*,*e*][1,4]thiabismine 5,5-dioxide **5** ... 22
4.2 Bromobis(2-(phenylsulfonyl)phenyl)bismuthane **6** ... 23
4.3 Synthesis of Expected Impurities **7** and **8** ... 24
5 Solubility Studies ... 26
5.1 Crude **1-Br** in Pure Solvents (manuscript Table 2) ... 26
5.2 Crude **1-Br** in Binary Solvent Mixtures (manuscript Table 3) ... 26
5.3 Crystallization Design of Experiments (manuscript Figure 1) ... 26
6 Calculation of PMI ... 29
7 References ... 30
8 Crystallographic Data ... 31
9 NMR Spectra ... 32

1 General Information

Procedures employing oxygen- and/or moisture-sensitive materials were performed with anhydrous solvents (*vide infra*) using standard inert-atmosphere techniques (atmosphere of anhydrous dinitrogen). Analytical thin-layer chromatography was performed on precoated aluminium-backed plates (Silica Gel 60 F254; Merck) and visualized using a combination of UV light (254 nm) and aqueous basic potassium permanganate stains. Manual flash column chromatography was performed using Scharlab 60 silica gel (35-70 mesh); automated flash column chromatography was performed on disposable columns pre-packed with 50 μm spherical silica gel using a Büchi C-850 equipped with a UV-vis DAD (200-800 nm) and an ELSD detector.

NMR spectra were recorded at 25 °C on Bruker Avance 400 or 500 spectrometers (^{1}H, 400/500 MHz; ^{13}C{^{1}H}, 101/126 MHz; ^{19}F NMR, 377/471 MHz). Chemical shifts are reported in ppm; coupling constants, *J*, are reported in Hz and are uncorrected for digitization. The following abbreviations (and their combinations) are used to label the multiplicities: s (singlet), d (doublet), t (triplet), q (quartet), m (multiplet), br (broad) and app. (apparent). ^{1}H and ^{13}C{^{1}H} chemical shifts are reported relative to tetramethylsilane, and are referenced to the appropriate residual solvent peaks:

- $CDCl_3$: δ_H = 7.26 ppm, δ_C = 77.16 ppm
- CD_3CN: δ_H = 1.94 ppm, δ_C = 118.26 ppm
- DMSO-d_6: δ_H = 2.50 ppm, δ_C = 39.52 ppm
- CD_3OD: δ_H = 3.31 ppm, δ_C = 49.00 ppm

^{19}F chemical shifts are reported relative to $BF_3\cdot OEt_2$.

Infrared spectra of neat compounds were recorded over the range 4000-600 cm^{-1} using either a PerkinElmerSpectrum 1000 Series FTIR spectrometer with an ATR diamond cell, or a Bruker Alpha FTIR spectrometer fitted with a Bruker Platinum ATR Quicksnap101 diamond cell. Melting points were measured using Stuart SMP10 or Gallenkamp melting point apparatus in open capillaries.

High resolution electrospray ionization mass spectra (HRMS) were recorded using a Bruker ESI-TOF MicroTOF II spectrometer.

Unless stated otherwise, reagent grade solvents (Fisher Technical) were employed. THF was dried using an Inert PureSolv Grubbs-type system (alumina columns, argon atmosphere). Anhydrous MeTHF was purchased and used as received. All reagents were obtained from commercial sources and, with the following exceptions, were used as received:

- $BiBr_3$ (Acros) was dried by stirring under vacuum (*ca* 10^{-2} mbar) at rt for 1 h.
- $BiCl_3$ (Acros) was dried by stirring under vacuum (*ca* 10^{-2} mbar) at rt for 1 h.
- CuCl (Acros), CuBr (Alfa Aesar), CuI (Alfa Aesar), and $ZnCl_2$ (Sigma Aldrich) were dried by stirring under vacuum (*ca* 10^{-2} mbar) at rt for 1 h before use in reaction optimization studies (manuscript Table 1).
- $ZnCl_2$ (Sigma Aldrich) was dried by stirring under vacuum (*ca* 10^{-2} mbar) at 150 °C for 3 h before use in 100 mmol scale reactions (manuscript Scheme 5).
- *n*-Butyllithium (Sigma-Aldrich) was titrated against *N*-benzyl benzamide before use.[1]

X-ray measurements were made on crystals coated in vacuum grease and mounted on a glass needle using Mo-Kα (λ = 0.71073 Å) radiation on a SuperNova Atlas diffractometer at 120(2) K. The structure was solved using Olex2 with the SHELXT structure solution program using Intrinsic Phasing and refined with the SHELXL refinement package using Least Squares minimisation.

DoE designs were created and analyzed using MODDE® Pro 13 (Sartorius); crystallization experiments for DoE studies were performed using a Crystal16® parallel crystallizer (Technobis Crystallization Systems B.V).

Safety Note: *n*-BuLi reacts violently with water and is potentially pyrophoric. It should only be used by trained individuals following a thorough risk assessment. For an excellent description of the safe sandling of alkyllithium reagents on both lab and plant scales, see:

> **Rathman, T.; Schwindeman, J. A. Preparation, Properties, and Safe Handling of Commercial Organolithiums: Alkyllithiums, Lithium sec-Organoamides, and Lithium Alkoxides. *Org. Process Res. Dev.* 2014, *18*, 1192–1210.**
>
> **https://doi.org/10.1021/op500161b**

2 B-to-Bi Transmetallation and Electrophilic Arylation (manuscript Scheme 3)

2.1 General Procedure for B-to-Bi Transmetallation (GP1)

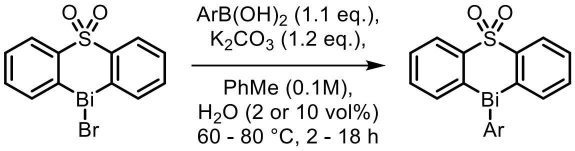

Under an atmosphere of air, a suspension of bismacycle bromide **1-Br** (253 mg, 0.50 mmol, 1.0 eq.), arylboronic acid (0.55 mmol, 1.1 eq.) and potassium carbonate (83 mg, 0.60 mmol, 1.2 eq.) in toluene (5 mL) and water (Z vol%) was heated at X °C for Y h in a round bottomed flask fitted with a water-cooled condenser. After heating for the specified time, the mixture was allowed to cool to room temperature, and an aliquot (*ca* 100 µL) of the reaction mixture was removed for analysis by ^{1}H NMR spectroscopy. The remaining reaction mixture was diluted with EtOAc or dichloromethane (50 mL), then washed with 2 M aq. NaOH (3 × 10 mL) and water (3 × 10 mL). The organic phase was dried over $MgSO_4$, filtered and concentrated *in vacuo* to give the aryl bismacycle. Any further purification was performed as described for individual entries.

2.2 Characterization Data for Aryl Bismacycle Products

10-(4-Fluorophenyl)-10*H*-dibenzo[*b,e*][1,4]thiabismine 5,5-dioxide 2a

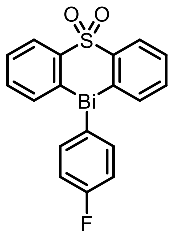

Synthesised according to *GP1* (X = 60 °C; Y = 2 h; Z = 10 vol%). Using 4-fluorophenylboronic acid (80.7 mg, 0.55 mmol) afforded the title compound (247 mg, 0.48 mmol, 95%) as a colourless solid. Characterization data were consistent with literature values: ^{1}H, ^{13}C{^{1}H} and ^{19}F NMR, HRMS, m.p.[2]

^{1}H NMR (400 MHz, CDCl$_3$): δ_H 8.38 (dd, *J* = 7.7, 1.5 Hz, 2H), 7.85 (dd, *J* = 7.1, 1.4 Hz, 2H), 7.76 – 7.67 (m, 2H), 7.41 (app. td, *J* = 7.6, 1.4 Hz, 2H), 7.36 (app. td, *J* = 7.3, 1.5 Hz, 2H), 7.12 – 7.03 (m, 2H).

^{13}C{^{1}H} NMR (101 MHz, CDCl$_3$): δ_C 163.2 (d, *J* = 248.4 Hz), 160.7 (br), 158.8 (br), 141.8, 141.0 (d, *J* = 7.0 Hz), 137.6, 133.7, 128.4, 127.3, 118.4 (d, *J* = 19.9 Hz).

^{19}F NMR (377 MHz, CDCl$_3$) δ_F -111.15 (tt, *J* = 9.4, 6.1 Hz).

υ_{max} (ATR)/cm^{-1}: 3043, 3029, 3002, 1573, 1562, 1483, 1434, 1298, 1286, 1251, 1222, 1160, 1150, 1122, 1106, 1088, 1075, 1045, 1029, 1011, 958, 935.

HRMS calcd. for $C_{18}H_{13}BiFO_2S^+$: 521.0419 [M+H]$^+$; found (ESI$^+$): 521.0418.

m.p./°C: 176 – 178.

10-(4-(Trifluoromethyl)phenyl)-10*H*-dibenzo[*b,e*][1,4]thiabismine 5,5-dioxide 2b

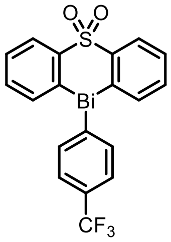

Synthesised according to *GP1* (X = 60 °C; Y = 2 h; Z = 10 vol%). Using (4-(trifluoromethyl)phenyl)boronic acid (108.9 mg, 0.55 mmol) afforded the title compound (263 mg, 0.46 mmol, 93%) as a colourless solid. Characterization data were consistent with literature values: ^{1}H, $^{13}C\{^{1}H\}$ and ^{19}F NMR, HRMS, m.p.[2]

^{1}H NMR (400 MHz, CDCl$_3$): δ_H 8.40 (dd, *J* = 7.5, 1.5 Hz, 2H), 7.90 (d, *J* = 7.7 Hz, 2H), 7.83 (dd, *J* = 7.2, 1.3 Hz, 2H), 7.62 (d, *J* = 7.7 Hz, 2H), 7.43 (app. td, *J* = 7.5, 1.4 Hz, 2H), 7.37 (app. td, *J* = 7.3, 1.5 Hz, 2H).

^{13}C{^{1}H} NMR (101 MHz, CDCl$_3$): δ_C 170.1 (br), 159.1 (br), 141.8, 139.3, 137.6, 133.8, 130.7 (q, *J* = 32.3 Hz), 128.6, 127.5, 127.4 (q, *J* = 3.7 Hz), 125.0 (q, *J* = 272.1 Hz.

^{19}F NMR (377 MHz, CDCl$_3$) δ_F -62.82.

υ_{max} (ATR)/cm^{-1}: 3053, 2923, 1593, 1564, 1440, 1387, 1323, 1303, 1284, 1252, 1151, 1115, 1098, 1089, 1073, 1041, 1008, 955.

HRMS calcd. for $C_{19}H_{13}BiF_3O_2S^+$: 571.0387 $[M+H]^+$; found (ESI$^+$): 571.0381.

m.p./°C: 178 – 179.

10-(4-Methoxyphenyl)-10*H*-dibenzo[*b,e*][1,4]thiabismine 5,5-dioxide <u>2c</u>

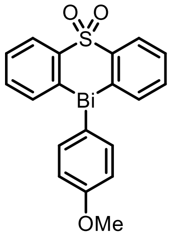

Synthesised according to *GP1* (X = 60 °C; Y = 2 h; Z = 10 vol%). Using (4-methoxyphenyl)boronic acid (88.8 mg, 0.55 mmol) afforded the title compound (195 mg, 0.37 mmol, 74%) as a colourless solid. Characterization data were consistent with literature values: ^{1}H and ^{13}C{^{1}H} NMR, HRMS, m.p.[2]

^{1}H NMR (400 MHz, CDCl$_3$): δ_H 8.37 (dd, *J* = 7.6, 1.4 Hz, 2H), 7.87 (dd, *J* = 7.2, 1.3 Hz, 2H), 7.67 – 7.63 (m, 2H), 7.39 (app. td, *J* = 7.6, 1.4 Hz, 2H), 7.33 (app. td, *J* = 7.3, 1.5 Hz, 2H), 6.95 – 6.91 (m, 2H), 3.80 (s, 3H).

^{13}C{^{1}H} NMR (101 MHz, CDCl$_3$): δ_C 160.0, 158.5 (br), 156.9 (br), 141.8, 140.4, 137.7, 133.5, 128.3, 127.1, 116.9, 55.2.

υ_{max} (ATR)/cm^{-1}: 3041, 2997, 2922, 2830, 1579, 1563, 1487, 1429, 1395, 1296, 1283, 1240, 1176, 1160 ,1148, 1122, 1105, 1085, 1071, 1058, 1024, 1012, 948, 930.

HRMS calcd. for $C_{19}H_{16}BiO_3S^+$: 533.0619 $[M+H]^+$; found (ESI$^+$): 533.0634.

m.p./°C: 223 – 224.

10-(4-(Dimethylamino)phenyl)-10*H*-dibenzo[*b*,*e*][1,4]thiabismine 5,5-dioxide <u>2d</u>

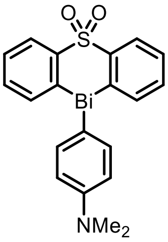

Synthesised according to *GP1* (X = 60 °C; Y = 2 h; Z = 10 vol%). Using (4-(dimethylamino)phenyl)boronic acid (96.4 mg, 0.55 mmol) afforded the title compound (218 mg, 0.40 mmol, 81%) as a colourless solid following trituration from absolute ethanol. Characterization data were consistent with literature values: ^{1}H and ^{13}C{^{1}H} NMR, HRMS, m.p.[2]

^{1}H NMR (400 MHz, $CDCl_3$): δ_H 8.36 (dd, *J* = 7.6, 1.4 Hz, 2H), 7.90 (dd, *J* = 7.2, 1.4 Hz, 2H), 7.62 – 7.54 (m, 2H), 7.38 (app. td, *J* = 7.5, 1.4 Hz, 2H), 7.32 (app. td, *J* = 7.3, 1.5 Hz, 2H), 6.77 – 6.69 (m, 2H), 2.96 (s, 6H).

^{13}C{^{1}H} NMR (101 MHz, $CDCl_3$): δ_C 158.2 (br), 152.1 (br), 150.5, 141.9, 140.1, 137.8, 133.3, 128.1, 127.0, 114.8, 40.2.

υ_{max} (ATR)/cm^{-1}: 3041, 2885, 2813, 1582, 1543, 1502, 1438, 1358, 1294, 1282, 1250, 1230, 1196, 1161, 1145, 1122, 1107, 1086, 1063, 1027.68, 1013, 962, 946.

HRMS calcd. for $C_{20}H_{19}BiNO_2S^+$: 546.0935 [M+H]$^+$; found (ESI$^+$): 546.0917.

m.p./°C: 202 – 203.

10-(2-(Trifluoromethyl)phenyl)-10*H*-dibenzo[*b,e*][1,4]thiabismine 5,5-dioxide 2e

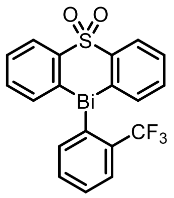

Synthesised according to *GP1* (X = 80 °C; Y = 18 h; Z = 2 vol%). Using (2-(trifluoromethyl)phenyl) boronic acid (108.7 mg, 0.55 mmol) afforded the title compound (223 mg, 0.39 mmol, 78%) as a colourless solid.

^{1}H NMR (400 MHz, $CDCl_3$): δ_H 8.40 (dd, *J* = 7.6, 1.5 Hz, 2H), 7.93 (dd, *J* = 7.9, 1.3 Hz, 1H), 7.85 (dd, *J* = 7.2, 1.3 Hz, 2H), 7.69 (app. d, *J* = 7.5 Hz, 1H), 7.48 (app. d, *J* = 7.5 Hz, 1H), 7.43 (app. td, *J* = 7.5, 1.4 Hz, 2H), 7.37 (app. td, *J* = 7.3, 1.5 Hz, 2H), 7.29 – 7.23 (m, 1H).

^{13}C{^{1}H} NMR (101 MHz, $CDCl_3$): δ_C 164.6 (br), 162.6 (br), 143.2, 141.9, 137.9, 136.5 (q, *J* = 29.9 Hz), 135.1, 133.9, 128.5, 128.3, 127.4, 125.9 (q, *J* = 5.0 Hz), 125.6 (q, *J* = 274.5 Hz).

^{19}F NMR (377 MHz, $CDCl_3$) δ_F -57.07.

υ_{max} (ATR)/cm^{-1}: 3046, 2922, 2851, 1562, 1426, 1308, 1295, 1286, 1251, 1170, 1151, 1133, 1099, 1085, 1072, 1028, 1011, 952.

HRMS calcd. for $C_{19}H_{13}BiF_3O_2S^+$: 571.0387 $[M+H]^+$; found (ESI$^+$): 571.0378.

m.p./°C: 206 – 207.

10-(2-(Trifluoromethoxy)phenyl)-10*H*-dibenzo[*b*,*e*][1,4]thiabismine 5,5-dioxide 2f

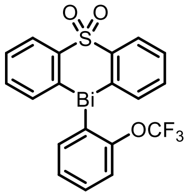

Synthesised according to *GP1* (X = 80 °C; Y = 18 h; Z = 2 vol%). Using (2-(trifluoromethoxy)phenyl)boronic acid (118 mg, 0.55 mmol) afforded the title compound (248 mg, 0.42 mmol, 84%) as a colourless solid. Characterization data were consistent with literature values: ^{1}H and $^{13}C\{^{1}H\}$ NMR, HRMS, m.p.[3]

^{1}H NMR (400 MHz, $CDCl_3$): δ_H 8.41 (dd, *J* = 7.5, 1.5 Hz, 2H), 7.89 (dd, *J* = 7.1, 1.4 Hz, 2H), 7.60 – 7.50 (m, 2H), 7.47 – 7.42 (m, 3H), 7.39 (app. td, *J* = 7.3, 1.5 Hz, 2H), 7.17 (app. td, *J* = 7.3, 1.1 Hz, 1H).

$^{13}C\{^{1}H\}$ NMR (126 MHz, $CDCl_3$): δ_C 159.6 (br), 157.5 (br), 153.7, 142.0, 141.4, 137.8, 133.8, 130.8, 130.6, 128.5, 127.4, 120.9 (q, *J* = 258.2 Hz), 120.2.

^{19}F NMR (377 MHz, $CDCl_3$) δ_F -56.64.

υ_{max} (ATR)/cm^{-1}: 3048, 2077, 1932, 1711, 1583, 1563, 1459, 1437, 1314, 1298, 1288, 1262, 1245, 1215, 1187, 1168, 1144, 1116, 1105, 1088, 1074, 1045, 1020, 1012, 951, 920.

HRMS calcd. for $C_{19}H_{13}BiF_3O_3S^+$: 587.0336 $[M+H]^+$; found (ESI$^+$): 587.0338.

m.p./°C: 179 – 180.

10-(2-Methoxyphenyl)-10*H*-dibenzo[*b*,*e*][1,4]thiabismine 5,5-dioxide 2g

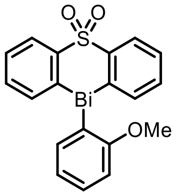

Synthesised according to GP1 (X = 80 °C; Y = 18 h; Z = 2 vol%). Using (2-methoxyphenyl)boronic acid (89.1 mg, 0.55 mmol) afforded the title compound (230 mg, 0.43 mmol, 86%) as a colourless solid.

^{1}H NMR (400 MHz, $CDCl_3$): δ_H 8.36 (dd, *J* = 7.8, 1.5 Hz, 2H), 7.91 (dd, *J* = 7.1, 1.4 Hz, 2H), 7.56 (dd, *J* = 7.2, 1.7 Hz, 1H), 7.43 – 7.29 (m, 5H), 7.17 (d, *J* = 8.2 Hz, 1H), 6.89 (app. td, *J* = 7.3, 1.1 Hz, 1H), 3.98 (s, 3H).

$^{13}C\{^1H\}$ NMR (101 MHz, $CDCl_3$): δ_C 162.7, 158.1 (br), 153.0 (br), 142.1, 139.7, 137.9, 133.3, 130.3, 128.1, 127.1, 125.0, 110.2, 55.8.

υ_{max} (ATR)/cm^{-1}: 3033, 2937, 2837, 1563, 1459, 1428, 1298, 1285, 1249, 1231, 1176, 1149, 1119, 1107, 1087, 1072, 1050, 1021, 1011.

HRMS calcd. for $C_{19}H_{16}BiO_3S^+$: 533.0619 $[M+H]^+$; found (ESI^+): 533.0623.

m.p./°C: 166 – 167.

10-(4-Fluoro-2-methylphenyl)-10*H*-dibenzo[*b,e*][1,4]thiabismine 5,5-dioxide 2h

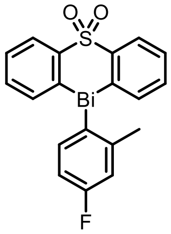

Synthesised according to *GP1* (X = 60 °C; Y = 2 h; Z = 10 vol%). Using (4-fluoro-2-methylphenyl)boronic acid (88.2 mg, 0.55 mmol) afforded the title compound (224 mg, 0.42 mmol, 83%) as a colourless solid. Characterization data were consistent with literature values: ^{1}H, ^{13}C{^{1}H} and ^{19}F NMR, HRMS, m.p.[4]

^{1}H NMR (400 MHz, CDCl$_3$): δ_H 8.38 (dd, *J* = 7.7, 1.4 Hz, 2H), 7.85 (dd, *J* = 7.3, 1.4 Hz, 2H), 7.48 (dd, *J* = 8.3, 6.6 Hz, 1H), 7.41 (app. td, *J* = 7.5, 1.3 Hz, 2H), 7.35 (app. td, *J* = 7.4, 1.5 Hz, 2H), 7.09 (dd, *J* = 10.3, 2.7 Hz, 1H), 6.72 (app. td, *J* = 8.6, 2.7 Hz, 1H), 2.54 (s, 3H).

^{13}C{^{1}H} NMR (101 MHz, CDCl$_3$): δ_C 163.2 (d, *J* = 247.8 Hz), 162.2 (br), 157.9 (br), 147.4 (d, *J* = 6.9 Hz), 143.4 (d, *J* = 7.4 Hz), 141.9, 137.8, 133.6, 128.5, 127.3, 117.6 (d, *J* = 20.0 Hz), 116.8 (d, *J* = 19.6 Hz), 26.6 (d, *J* = 1.9 Hz).

^{19}F NMR (377 MHz, CDCl$_3$) δ_F -112.09 (ddd, *J* = 10.1, 8.8, 6.6 Hz).

υ_{max} (ATR)/cm^{-1}: 3050, 1588, 1564, 1465, 1429, 1392, 1297, 1272, 1248, 1221, 1175, 1151, 1134, 1118, 1106, 1087, 1074, 1011, 938.

HRMS calcd. for $C_{19}H_{15}BiFO_2S^+$: 535.0575 [M+H]$^+$; found (ESI$^+$): 535.0576.

m.p./°C: 219 – 220.

10-Mesityl-10*H*-dibenzo[*b*,*e*][1,4]thiabismine 5,5-dioxide <u>2i</u>

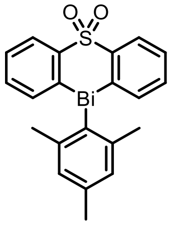

Synthesised according to *GP1* (X = 80 °C; Y = 18 h; Z = 10 vol%). Using mesitylboronic acid (99.0 mg, 0.55 mmol) afforded the title compound (223 mg, 0.41 mmol, 83%) as a colourless solid following trituration from absolute ethanol. Characterization data were consistent with literature values: ^{1}H and ^{13}C{^{1}H} NMR, HRMS, m.p.[2]

^{1}H NMR (400 MHz, CDCl$_3$): δ_H 8.42 (dd, *J* = 7.8, 1.3 Hz, 2H), 8.03 (dd, *J* = 7.4, 1.3 Hz, 2H), 7.42 (app. td, *J* = 7.6, 1.3 Hz, 2H), 7.32 (app. td, *J* = 7.4, 1.4 Hz, 2H), 7.04 (s, 2H), 2.27 (br s, 6H), 1.26 (s, 3H). *The signals corresponding to the ortho-Me substituents exhibit rotameric broadening and overlap with the signal for the para-Me, preventing reliable integration.*

^{13}C{^{1}H} NMR (101 MHz, CDCl$_3$): δ_C 165.6 (br), 155.3 (br), 146.5 (br), 141.2, 139.2, 137.9, 133.2, 129.9 (br), 128.5, 127.4, 28.1, 21.3.

υ_{max} (ATR)/cm^{-1}: 3047, 2916, 1562, 1438, 1427, 1375, 1296, 1282, 1249, 1179, 1164, 1147, 1133, 1119, 1106, 1088, 1072, 1012.

HRMS calcd. for $C_{21}H_{20}BiO_2S^+$: 545.0983 [M+H]$^+$; found (ESI$^+$): 545.0996.

m.p./°C: 163 – 164.

10-(Thiophen-3-yl)-10*H*-dibenzo[*b*,*e*][1,4]thiabismine 5,5-dioxide 2j

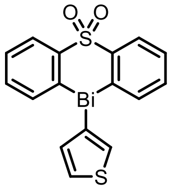

Synthesised according *GP1* (X = 60 °C; Y = 2 h; Z = 10 vol%). Using thiophen-3-ylboronic acid (77.6 mg, 0.55 mmol) afforded the title compound (225 mg, 0.44 mmol, 88%) as a colourless solid. Characterization data were consistent with literature values: ^{1}H and ^{13}C{^{1}H} NMR, HRMS, m.p.[2]

^{1}H NMR (400 MHz, CDCl$_3$): δ_H 8.37 (dd, *J* = 7.5, 1.6 Hz, 2H), 7.91 (dd, *J* = 7.2, 1.4 Hz, 2H), 7.66 (dd, *J* = 2.6, 1.0 Hz, 1H), 7.46 – 7.37 (m, 3H), 7.35 (app. td, *J* = 7.3, 1.5 Hz, 2H), 6.97 (dd, *J* = 4.8, 1.0 Hz, 1H).

^{13}C{^{1}H} NMR (101 MHz, CDCl$_3$): δ_C 158.8 (br), 157.2 (br), 141.8, 137.7, 136.8, 136.0, 133.4, 129.0, 128.4, 127.3.

υ_{max} (ATR)/cm^{-1}: 3052, 2921, 1561, 1438, 1423, 1297, 1284, 1250, 1147, 1134, 1118, 1106, 1084, 1070, 1027, 1011.

HRMS calcd. for $C_{16}H_{11}BiNaO_2S_2^+$: 530.9897 [M+Na]$^+$; found (ESI$^+$): 530.9884.

m.p./°C: 182 – 183.

10-(6-Methoxypyridin-3-yl)-10*H*-dibenzo[*b*,*e*][1,4]thiabismine 5,5-dioxide 2k

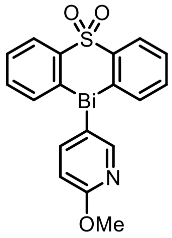

Synthesised according to *GP1* (X = 60 °C; Y = 3 h; Z = 10 vol%). Using (6-methoxypyridin-3-yl)boronic acid (87.9 mg, 0.55 mmol) afforded the title compound (218 mg, 0.41 mmol, 83%) as a colourless solid. Characterization data were consistent with literature values: ^{1}H and ^{13}C{^{1}H} NMR, HRMS, m.p.[2]

^{1}H NMR (400 MHz, $CDCl_3$): δ_H 8.42 – 8.35 (m, 3H), 7.86 (dd, *J* = 7.2, 1.3 Hz, 2H), 7.74 (dd, *J* = 8.3, 2.0 Hz, 1H), 7.41 (app .td, *J* = 7.4, 1.5 Hz, 2H), 7.36 (app. td, *J* = 7.4, 1.6 Hz, 2H), 6.70 (dd, *J* = 8.2, 0.8 Hz, 1H), 3.93 (s, 3H).

^{13}C{^{1}H} NMR (101 MHz, $CDCl_3$): δ_C 164.3, 157.6 (br), 156.0, 151.8 (br), 149.1, 141.8, 137.6, 133.7, 128.5, 127.3, 114.7, 53.5.

υ_{max} (ATR)/cm^{-1}: 3046, 3002, 2950, 1571, 1549, 1474, 1423, 1346, 1282, 1244, 1178, 1148, 1124, 1106, 1086, 1070, 1029, 1013, 997, 966.

HRMS calcd. for $C_{18}H_{15}BiNO_3S^+$: 534.0571 [M+H]$^+$; found (ESI$^+$): 534.0595.

m.p./°C: 233 – 234.

2.3 Procedures for Electrophilic Arylation

Arylation of 2-Naphthol

According to the procedure of *Nature Chem.* **2020**, *12*, 260-290, without modification:

Under an atmosphere of air, a suspension of bismacycle bromide **1-Br** (253 mg, 0.50 mmol), 4-fluorophenylboronic acid (77.0 mg, 0.55 mmol), potassium carbonate (83 mg, 0.60 mmol) and 3,3',5,5'-tetrakis(trifluoromethyl)-1,1'-biphenyl (internal standard for ^{19}F NMR; 17.8 mg, 0.042 mmol) in toluene (5 mL) and water (0.5 mL) was heated at 60 °C for 2 h in a round bottomed flask fitted with a water-cooled condenser. The mixture was allowed to cool to rt before 2-naphthol (64.9 mg, 0.45 mmol) was added, followed by *m*CPBA (80% purity; 162 mg, 0.75 mmol). The reaction was stirred for 5 min at rt, then an aliquot of the reaction mixture was removed for analysis by ^{19}F NMR spectroscopy. Yield: 81%.

Arylation of 2,6-Dimethylphenol

According to the procedure of *Nature Chem.* **2023**, *15*, 386-394, without modification:

Under an atmosphere of air, a suspension of bismacycle bromide **1-Br** (253 mg, 0.50 mmol), 4-fluorophenylboronic acid (77.0 mg, 0.55 mmol), potassium carbonate (83 mg, 0.60 mmol) and 3,3',5,5'-tetrakis(trifluoromethyl)-1,1'-biphenyl (internal standard for ^{19}F NMR; 17.8 mg, 0.042 mmol) in toluene (5 mL) and water (0.5 mL) was heated at 60 °C for 2 h in a round bottomed flask fitted with a water-cooled condenser. The mixture was allowed to cool to rt before aq. NaOH (2.0 M; 2 mL) was added and vigorously stirred for 5 min. The organic phase was removed, and the remaining aqueous portion was extracted with toluene (2 mL). The combined organic portions were filtered through a pad of $MgSO_4$ (1 cm^3) into a round-bottom flask containing 2,6-dimethylphenol (61.2 mg, 0.5 mmol). The mixture was stirred as *m*CPBA (80% purity; 108 mg, 0.5 mmol) was added portionwise over 5 min and left to stir for a further 30 min. An aliquot of the reaction mixture was removed for analysis by ^{19}F NMR spectroscopy. Yield: 83%.

Arylation of Dimedone

According to the procedure of *Angew. Chem.* Int. Ed. **2022**, *61*, e202210840, with the addition of a NaOH-wash after transmetallation:

Under an atmosphere of air, a suspension of bismacycle bromide **1-Br** (253 mg, 0.50 mmol), 2-methyl-4-fluorophenylboronic acid (84.5 mg, 0.55 mmol), potassium carbonate (83 mg, 0.60 mmol) and 3,3',5,5'-tetrakis(trifluoromethyl)-1,1'-biphenyl (internal standard for ^{19}F NMR; 17.8 mg, 0.042 mmol) in toluene (5 mL) and water (0.5 mL) was heated at 60 °C for 2 h in a round bottomed flask fitted with a water-cooled condenser. The mixture was allowed to cool to rt before aq. NaOH (2.0 M; 2 mL) was added and vigorously stirred for 5 min. The organic phase was removed, and the remaining aqueous portion was extracted with toluene (2 mL). The combined organic portions were concentrated to dryness, then Selectfluor (177 mg, 0.5 mmol), sodium benzoate (144 mg, 1.0 mmol) and benzoic acid (61.0 mg, 0.5 mmol) were added. MeCN (2.5 mL) was added, and the reaction mixture was stirred at rt for 0.5 h before diluting with toluene (7.5 mL). Dimedone (70.1 mg, 0.5 mmol) was added, and the mixture was stirred at rt for 0.5 h then heated at 80 °C for 2 h. An aliquot of the reaction mixture was removed for analysis by ^{19}F NMR spectroscopy. Yield: 85%.

Arylation of 2-Pyridone

According to the procedure of *Angew. Chem.* Int. Ed. **2022**, *61*, e202212873, without modification:

Under an atmosphere of air, a suspension of bismacycle bromide **1-Br** (253 mg, 0.50 mmol), 4-fluorophenylboronic acid (77.0 mg, 0.55 mmol), potassium carbonate (83 mg, 0.60 mmol) and 3,3',5,5'-tetrakis(trifluoromethyl)-1,1'-biphenyl (internal standard for ^{19}F NMR; 17.8 mg, 0.042 mmol) in toluene (5 mL) and water (0.5 mL) was heated at 60 °C for 2 h in a round bottomed flask fitted with a water-cooled condenser. The mixture was allowed to cool to rt, then EtOAc (5 mL) and 2 M aq. NaOH (5 mL) were added and the biphasic mixture was stirred vigorously for 5 min then allowed to separate. The organic layer was removed by syringe and filtered through a pad of $MgSO_4$ into a round bottomed flask. The aqueous portion was extracted with EtOAc (2 × 5 mL) and the combined organic portions were concentrated to dryness. Selectfluor (177 mg, 0.5 mmol) was added, followed by MeCN (5 mL). The reaction mixture was stirred at rt for 15 min, then 2-pyridone (47.6 mg, 0.5 mmol) and benzoic acid (61.0 mg, 0.5 mmol) were added, and the reaction mixture was heated at 80 °C for 2 h. An aliquot of the reaction mixture was removed for analysis by ^{19}F NMR spectroscopy. Yield: 72%.

3 Additional Synthesis Procedures for Bismacycle Halides

3.1 10-Chloro-10*H*-dibenzo[*b*,*e*][1,4]thiabismine 5,5-dioxide 1-Cl

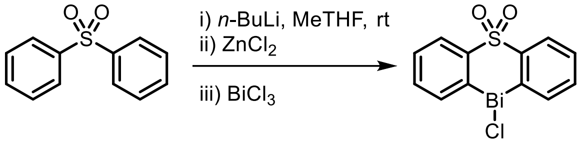

A flame dried Schlenk flask containing diphenyl sulfone (223 mg, 1.0 mmol, 1.0 eq.) was evacuated and backfilled thrice with anhydrous dinitrogen, then anhydrous MeTHF (4 mL) was added. *n*-butyllithium (2.25 M in hexanes; 0.88 mL, 2.0 mmol, 2.0 eq.) was added at room temperature and the resulting solution was stirred for 15 min. A solution of $ZnCl_2$ (1.9 M in 2MeTHF; 11.5 mL, 22.0 mmol) was added dropwise at room temperature.

A separate flame dried Schlenk flask containing $BiCl_3$ (321 mg, 1.04 mmol, 1.04 eq.) was evacuated and backfilled thrice with anhydrous dinitrogen, then anhydrous MeTHF (4 mL) was added to give a suspension. The solution of dilithiodiphenyl sulfone was added *via* cannula at room temperature and the resulting suspension was stirred overnight. The mixture was quenched with MeOH (1 mL), filtered through glass fibre filter paper, dried with $MgSO_4$ and concentrated to approximately 2 mL. TBME (4 mL) was added, and the resulting suspension was stirred for 1 h. The white solid was collected by Buchner filtration and washed with TBME to afford **1-Cl** as a hygroscopic white solid (198 mg, 0.43 mmol, 41%). Characterization data were consistent with literature values: ^{1}H NMR.[5]

^{1}H NMR (400 MHz, CDCl$_3$): δ_H ^{1}H NMR (400 MHz, CDCl$_3$) δ 8.84 (dd, *J* = 7.3, 1.1 Hz, 2H), 8.35 (dd, *J* = 7.7, 1.2 Hz, 2H), 7.74 (app. td, *J* = 7.4, 1.2 Hz, 2H), 7.48 (app. td, *J* = 7.6, 1.1 Hz, 2H).

^{13}C{^{1}H} NMR (101 MHz, CDCl$_3$): δ_C 178.9, 140.4, 136.1, 135.8, 129.0, 128.6.

υ_{max} (ATR)/cm^{-1}: 3042, 1621, 1557, 1437, 1425, 1291, 1251, 1139, 1131, 1109, 1098, 1085, 1067, 1028, 1008, 991.

HRMS calcd. for $C_{12}H_8BiO_2S^+$: 425.0044 [M-Cl]$^+$; found (ESI$^+$): 425.0042.

m.p./°C: Could not be obtained due to hygroscopicity, and hence high water content in the solid.

3.2 10-Bromo-10*H*-dibenzo[*b*,*e*][1,4]thiabismine 5,5-dioxide 1-Br

3.2.1 1-Br *via* Magnesiation (manuscript Scheme 4, entry 2)

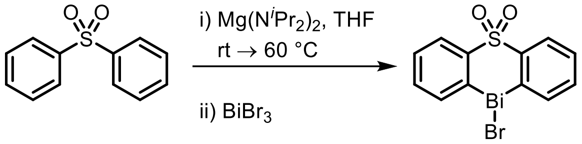

A flame dried Schlenk flask containing diphenyl sulfone (223 mg, 1.0 mmol, 1.0 eq.) was evacuated and backfilled thrice with anhydrous dinitrogen, then anhydrous THF (2 mL) was added. Magnesium bis(diisopropylamide)[6] (0.85 M in Et_2O, 2.4 mL, 2.0 eq.; titrated against BzOH with 4(phenylazo)diphenylamine as indicator[7]) was added at room temperature and the resulting solution was stirred for 1 h at room temperature, and then for 1 h at 60 °C. The mixture was allowed to cool to room temperature, then an aliquot (*ca* 100 µL) was removed and quenched with D_2O prior to analysis by ^{1}H NMR spectroscopy.

A separate flame dried Schlenk flask containing $BiBr_3$ (476 mg, 1.04 mmol, 1.04 eq.) was evacuated and backfilled thrice with anhydrous dinitrogen, then anhydrous THF (2 mL) was added to give a homogeneous solution. This solution was cooled to 0 °C, then the Grignard reagent was added dropwise *via* cannula. The mixture was stirred at room temperature overnight then quenched with sat. aq. NH_4Br (2.5 mL) and extracted with EtOAc (5 × 4 mL). The combined organic portions were filtered through a pad of Celite®, dried over $MgSO_4$ and concentrated *in vacuo*. The residue was triturated from MeCN (2 mL), and the solid was collected by Buchner filtration and washed with MeCN to give the title compound as a colourless solid (142 mg, 0.28 mmol, 28%).

3.2.2 1-Br *via* Transmetallation to CuCl (manuscript Table 1, entry 6)

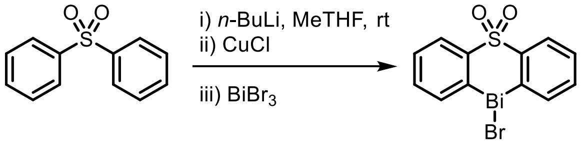

A flame dried Schlenk flask containing diphenyl sulfone (2.25 g, 10.0 mmol, 1.0 eq.) was evacuated and backfilled thrice with anhydrous dinitrogen, then anhydrous MeTHF (40 mL) was added. The flask was submerged in an ambient-temperature water bath, then *n*-butyllithium (2.32 M in hexanes; 8.6 mL, 20 mmol, 2.0 eq.) was added at room temperature and the resulting solution was stirred for 15 min.

CuCl (2.19 g, 22.0 mmol, 2.2 eq.) was dried under vacuum at 150 °C for 3 h in a separate flame dried Schlenk RBF. After cooling, anhydrous MeTHF (20 mL) was added to give a suspension. The flask was submerged in an ambient-temperature water bath, then the solution of dilithiodiphenyl sulfone was added *via* cannula at room temperature. The transfer was made complete by rinsing the dilithiodiphenyl sulfone flask with MeTHF (2 mL), and the resulting dark solution was stirred for 30 min.

A separate flame dried Schlenk flask containing $BiBr_3$ (4.67 g, 10.4 mmol, 1.04 eq.) was evacuated and backfilled thrice with anhydrous dinitrogen, then anhydrous MeTHF (40 mL) was added to give a homogeneous solution. The organocopper solution was added *via* cannula at room temperature. The transfer was made complete by rinsing the organocopper flask with MeTHF (2 mL), and the resulting suspension was stirred overnight. The mixture was quenched with aq. NH_4Br (1 M, 10 mL), and the aqueous phase was extracted with EtOAc (3 × 10 mL). The combined organic portions were washed with water (3 × 25 mL), aq. NH_4Br (1 M, 3 × 25 mL), dried with $MgSO_4$, then filtered through a pad of Celite®. The filter cake was washed with EtOAc (50 mL) and the filtrate was concentrated *in vacuo* to afford crude **1-Br** (4.62 g, 8.6 mmol 84%, 94% w/w) as a white powder. Purity by mass was determined by ^{1}H NMR using 1,3,5-trimethoxybenzene as internal standard. The material was used for solubility studies and crystallization DoE (manuscript Figure 1).

3.2.3 1-Br *via* Transmetallation to $ZnCl_2$ (manuscript Table 1, entry 7)

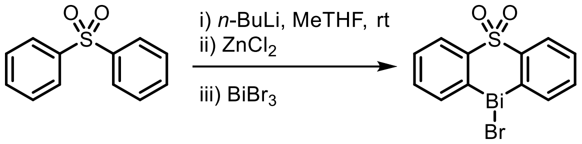

A flame dried three-necked 100 mL RBF fitted with a thermometer and containing diphenyl sulfone (2.25 g, 10.0 mmol, 1.0 eq.) was evacuated and backfilled thrice with anhydrous dinitrogen, then anhydrous MeTHF (40 mL) was added to give a colorless solution. *n*-butyllithium (2.38 M in hexanes; 8.6 mL, 20 mmol) was added at room temperature over 10 min, and the resulting solution was stirred for 15 min. $ZnCl_2$ solution (1.9 M in MeTHF; 11.5 mL, 22.0 mmol) was added dropwise at room temperature, and the resulting solution was stirred for 15 min.

A separate flame dried three-necked 250 mL RBF containing $BiBr_3$ (4.67 g, 10.4 mmol, 1.04 eq.) was evacuated and backfilled thrice with anhydrous dinitrogen, then anhydrous MeTHF

(40 mL) was added to give a homogeneous solution. The organozinc solution was added *via* cannula at room temperature and the resulting suspension was stirred overnight. The mixture was quenched with aq. NH_4Br (1 M, 10 mL) and the organic phase was separated and washed with aq. NH_4Br (1 M, 3 × 25 mL) and water (3 × 25 mL), dried with $MgSO_4$, then filtered through glass fibre filter paper. The filtrate was concentrated *in vacuo* to afford crude **1-Br** (4.87 g, 7.8 mmol, 76%, 81% w/w) as a pale yellow powder. Purity by mass was determined by ^{1}H NMR using 1,3,5-trimethoxybenzene as internal standard.

4 Identification of Process Impurities

4.1 10-(2-(Phenylsulfonyl)phenyl)-10*H*-dibenzo[*b*,*e*][1,4]thiabismine 5,5-dioxide 5

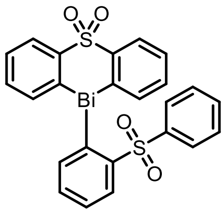

This procedure follows from the 100 mmol scale synthesis of compound ***1-Br*** *detailed in the manuscript*. The mother liquor remaining after isolation of the third crop of **1-Br** was concentrated *in vacuo* and subjected to silica gel column chromatography (0 – 20% EtOAc in cyclohexane). Solids that formed in column fractions enriched in **5** were collected by Buchner filtration, washed with cyclohexane and dried under a flow of air to give the title compound as colourless crystals.

^{1}H NMR (400 MHz, DMSO): δ_H ^{1}H NMR (400 MHz, DMSO) δ 8.33 – 8.24 (m, 3H), 8.21 – 8.14 (m, 2H), 7.99 – 7.91 (m, 2H), 7.83 (dd, *J* = 7.6, 1.2 Hz, 1H), 7.81 – 7.75 (m, 1H), 7.75 – 7.68 (m, 2H), 7.62 (app. td, *J* = 7.6, 1.3 Hz, 1H), 7.52 – 7.41 (m, 5H).

^{13}C{^{1}H} NMR (101 MHz, DMSO): δ_C 168.6 (br), 167.4 (br), 145.7, 142.2, 141.0, 140.5, 138.1, 136.0, 134.0, 133.5, 130.5, 130.0, 129.0, 128.2, 127.9, 126.3.

υ_{max} (ATR)/cm^{-1}: 1562, 1445, 1300, 1289, 1244, 1145, 1114, 1090, 1072, 1023, 995.

HRMS calcd. For $C_{24}H_{18}BiO_4S_2^+$: 643.0445 [M+H]$^+$; found (ESI$^+$): 643.0416.

m.p./°C: 244 – 245.

4.2 Bromobis(2-(phenylsulfonyl)phenyl)bismuthane 6

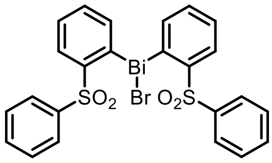

*This procedure follows from the 100 mmol scale synthesis of compound **1-Br** detailed in the manuscript*. The mother liquor remaining after isolation of the third crop of **1-Br** was concentrated *in vacuo* and subjected to silica gel column chromatography (0 – 20% EtOAc in cyclohexane). Fractions enriched with the reported compound were identified by ^{1}H NMR, combined, and resubjected to silica gel column chromatography (10% EtOAc in cyclohexane). The title compound crystallised as a colourless solid from $CDCl_3$.

^{1}H NMR (400 MHz, $CDCl_3$): δ_H 9.94 (dd, *J* = 7.8, 1.1 Hz, 2H), 8.14 – 8.08 (m, 2H), 8.08 – 8.05 (m, 4H), 7.92 (dd, *J* = 7.9, 1.2 Hz, 2H), 7.72 – 7.64 (m, 4H), 7.59 (dd, *J* = 8.5, 7.2 Hz, 4H).

^{13}C{^{1}H} NMR (101 MHz, $CDCl_3$): δ_C 194.7 (br)*, 145.6, 141.4, 139.7, 139.7, 134.8, 132.4, 130.4, 130.0, 128.3. **Observed by HMBC.*

υ_{max} (ATR)/cm^{-1}: 3060, 2922, 1557, 1446, 1421, 1257, 1135, 1112, 1095, 1064, 1031, 1021, 1008, 995, 901.

HRMS calcd. For $C_{24}H_{18}BiBrNaO_4S_2^+$: 744.9526 [M+Na]$^+$; found (ESI$^+$): 744.9543.

m.p./°C: 202 – 203.

4.3 Synthesis of Expected Impurities 7 and 8

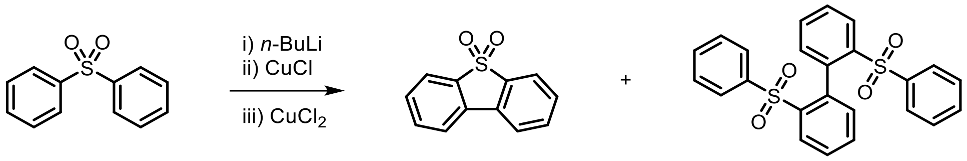

A flame dried Schlenk flask containing diphenyl sulfone (1.13 g, 5.0 mmol, 1.0 eq.) was evacuated and backfilled thrice with anhydrous dinitrogen, then anhydrous THF (15 mL) was added to give a homogeneous solution. The flask was submerged in an ambient temperature water bath, then *n*-butyllithium (2.37 M in hexanes; 2.1 mL, 5.00 mmol) was added at room temperature. The resulting solution was stirred for 15 min.

A separate flame dried Schlenk flask containing CuCl (0.26 g, 2.5 mmol, 0.5 eq.) was evacuated and backfilled thrice with anhydrous dinitrogen, then the solution of lithiodiphenyl sulfone was added *via* canula at room temperature. The resulting brown suspension was stirred for 1 h, then solid $CuCl_2$ (0.68 g, 5.0 mmol, 1.0 eq.) was added against a flow of dinitrogen. The mixture became olive green and was stirred overnight, then filtered through a pad of Celite®. The filtrate was concentrated *in vacuo* then purified by silica gel column chromatography (0 – 50% EtOAc/in cyclohexane).

Dibenzo[*b,d*]thiophene 5,5-dioxide 7

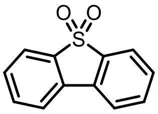

Eluted at 10% EtOAc in cyclohexane as a colourless solid (80.0 mg, 0.370 mmol, 7%). Characterization data were consistent with literature values: ^{1}H, and ^{13}C{^{1}H} NMR, HRMS, m.p.[8]

^{1}H NMR (400 MHz, $CDCl_3$): δ_H 7.86 – 7.78 (m, 4H), 7.65 (app. td, *J* = 7.6, 1.2 Hz, 2H), 7.54 (app. td, *J* = 7.6, 1.0 Hz, 2H).

^{13}C {^{1}H} NMR (101 MHz, $CDCl_3$): δ_C 137.9, 134.0, 131.8, 130.6, 122.4, 121.7.

υ_{max} (ATR)/cm^{-1}: 3079, 2922, 1728, 1571, 1479, 1447, 1283, 1152, 1119, 1099, 1074, 1045, 1003, 943.

HRMS calcd. For $C_{12}H_9O_2S^+$: 217.0318 $[M+H]^+$; found (ESI^+): 217.0315.

m.p./°C: 233 – 234

2,2′-Bis(phenylsulfonyl)-1,1′biphenyl 8

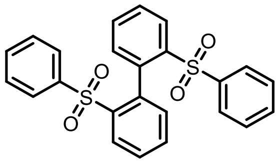

Eluted at 50% EtOAc in cyclohexane as a colourless solid (210.3 mg, 0.484 mmol, 10%).

^{1}H NMR (400 MHz, $CDCl_3$): δ_H 8.18 (dd, J = 8.0, 1.4 Hz, 2H), 7.59 (td, J = 7.7, 1.4 Hz, 2H), 7.56 – 7.47 (m, 4H), 7.47 – 7.41 (m, 4H), 7.37 (dd, J = 8.4, 7.2 Hz, 4H), 7.02 (dd, J = 7.6, 1.4 Hz, 2H).

^{13}C {^{1}H} NMR (101 MHz, $CDCl_3$): δ_C 141.3, 139.7, 137.5, 133.3, 133.0, 132.1, 129.6, 129.0, 128.9, 128.4.

υ_{max} (ATR)/cm^{-1}: 3061, 2924, 1732, 1582, 1567, 1475, 1446, 1315, 1303, 1253, 1154, 1129, 1095, 1073, 1058, 1022, 1000, 957.

HRMS calcd. For $C_{24}H_{19}O_4S_2^+$: 435.0719 $[M+H]^+$; found (ESI^+): 435.0734.

m.p./°C: >155 (decomp).

5 Solubility Studies

5.1 Crude <u>1-Br</u> in Pure Solvents (manuscript Table 2)

The appropriate solvent (0.5 mL, 10 volumes) was added to a 2 mL vial containing crude **1-Br** (50 mg; 81:6:9 mixture of **1-Br**:**5**:**6**) and the mixture was stirred (800 rpm) at room temperature for 2 h. The contents of the vial were filtered through a filter cartridge (pore size = 0.45 μm) into an NMR tube and the solvent was removed *in vacuo*. A stock solution of 1,3,5-trimethoxy benzene internal standard (201.3 mg) in $CDCl_3$ (5.00 mL) was prepared, and an aliquot (500 μL) of this solution was added into the NMR tube and ^1H NMR spectrum was measured. Solubility was determined as the quantity of product or impurity observed in solution, which corresponds to the loss of material from the crude sample to filtrate.

5.2 Crude <u>1-Br</u> in Binary Solvent Mixtures (manuscript Table 3)

The appropriate solvent (MeTHF or THF) was added to a 2 mL vial containing crude **1-Br** (100 mg; 81:6:9 mixture of **1-Br**:**5**:**6**) and the mixture was stirred (800 rpm) at 50 °C, then the appropriate anti-solvent (EtOH, TBME or cyclohexane) added. The mixture was held for 30 min before cooling to room temperature overnight. **1-Br** was collected by Buchner filtration and dried under a flow of air to give the % **1-Br** material isolated; for all entries, purity was determined to be >95% w/w (quantitative ^1H NMR spectroscopy *vs* 1,3,5-trimethoxybenzene).

5.3 Crystallization Design of Experiments (manuscript Figure 1)

Specific conditions are detailed in Table S1; a representative ^1H NMR spectrum used for analysis is presented in Figure S1.

<u>Experimental procedure:</u> THF (0.3 mL, 3 volumes) was added to a 1 mL vial containing crude **1-Br** (100 mg; 81:6:9 mixture of **1-Br**:**5**:**6**). The mixture was stirred and heated to 50 °C at a rate of 1 °C/min, then held at 50 °C for 15 minutes. EtOH (preheated to 50 °C) was added, and the mixture was heated at 50 °C for a further 15 min. The mixture was then cooled to the desired end temperature at a rate of -1 °C/min, then held at the end temperature for 20 h with stirring. The stirring was stopped, and an aliquot (100 μL) of the supernatant was analyzed by ^1H NMR

spectroscopy (using double presaturated solvent suppression); quantitative integrations were calculated against 1,3,5-trimethoxybenzene as internal standard.

<u>Analytical procedure:</u> An example of the analysis procedure is given for experiment 10 (mid-point; run order 5, 5 V EtOH, 7 °C, 400 rpm):

- Bismacycle **1-Br** (8.89 to 8.84 ppm, 2H, integral = 3.87) was integrated against 1,3,5-trimethoxybenzene (6.10 to 5.84 ppm, 3H, integral = 100) as internal standard.
- Internal standard: molecular weight = 168.19 g/mol, mass weighed = 10.51 mg
- **1-Br**: molecular weight = 505.14 g/mol, mass of crude weighed = 100.40 mg, 94.3% purity. Purity corrected mass of **1-Br** = 94.68 mg
- Mass of **1-Br** calculated for 100 µL sample = 1.83 mg. Therefore for 800 µL (300 µL THF + 500 µL EtOH), mass of **1-Br** = 14.66 mg (0.029 mmol).
- Therefore 14.66 / 94.68 × 100 = **15.5% in filtrate**, so remainder is 85.5% present as solid.

Table S1. Conditions used for crystallization Design of Experiments (DoE).

Exp no.	Run order	EtOH / V	*T* / °C	Stirring Rate / rpm	% 1-Br	
					filtrate	solid
7	1	3	20	700	16.6	83.4
11	2	5	7	400	14.3	85.7
6	3	7	-6	700	10.1	89.9
8	4	7	20	700	12.6	87.4
10	5	5	7	400	15.5	84.5
2	6	7	-6	100	10.6	89.5
4	7	7	20	100	15.3	84.7
9	8	5	7	400	15.7	84.3
3	9	3	20	100	24.8	75.2
5	10	3	-6	700	20.0	80.0
1	11	3	-6	100	21.3	78.7

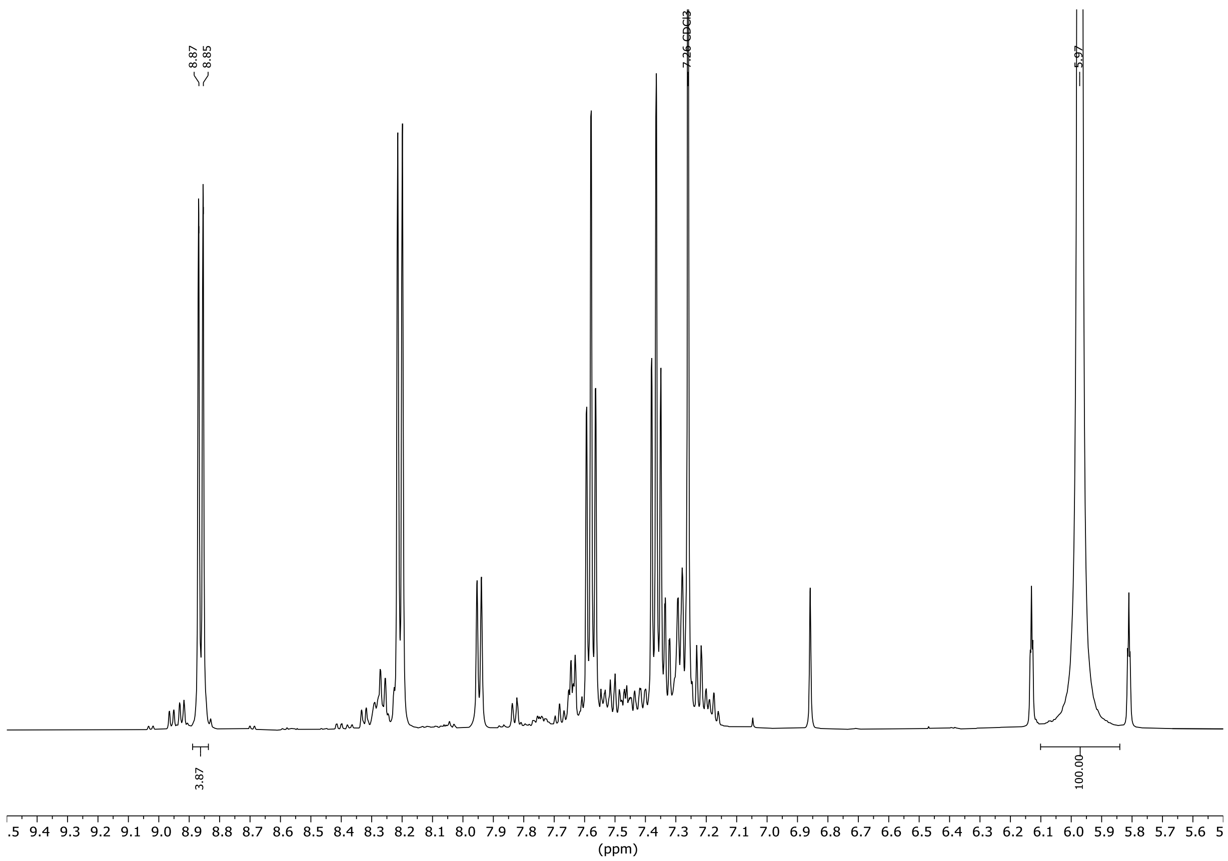

Figure S1. Representative ^{1}H NMR spectrum (500 MHz, $CDCl_3$; double presaturated solvent suppression) used in analysis of crystallization DoE.

6 Calculation of PMI

Calculation of PMI was based on the synthesis of bismacycle **1-OTs** illustrated in manuscript Scheme 2A, and the synthesis of bismacycle **1-Br** illustrated in manuscript Scheme 5.

Table S2. Calculation of PMI.

		Synthesis of **1-OTs**			**1-Br**
		**3** (steps 1-2) [44.8 mmol, 90%]	**1-OTs** (steps 3-6) [38.1 mmol, 66%]	**1-OTs** total	[63.8 mmol, 62%]
Solvent	DCM	0	400 (100×3+100)	400	0
	THF	135 (60+75)	220 (100+120)	355	195 (130+36+29)
	Et_2O	300 (100×3)	68.1 (22.7*×3)	368.1	0
	MeCN	0	148.1 (80+22.7*×3)	148.1	0
	EtOH	50	0	50	452 (300+84+68)
	MeOH	0	10	10	0
	MeTHF	0	0	0	1020 (220+400+400)
	total	485	846.2	1331.2	1667
Aq. waste		100	200	300	1500
Solid waste	Silica/ cm^3	33.2	71.4 (33.2×2)		0
	Silica/ g	87.9	189.1		
	$MgSO_4$/ g	5	5	10	5
	Total waste/ g	92.9	194.1		5
	Starting materials/ g	31.1	73.1		156.4
	Solvent/ g	373.2	900.2		1400.9
	Work up/aq.	100	200		1500
	Solid waste/ g	92.9	194.1		5
	Total mass of process/ g	597.3	1367.4		3062.3
	Product mass/ g	22.2	16.9		32.2
	PMI	27	81	**108**	**95**

Notes:

- For values not specified in previous procedures (such as volumes of solvent for extractions, filter cake washes, and amount of silica used) reasonable estimates have been made.
- Filter cake wash. Volume based on mass of product × 3 washes
- Mass of silica waste based on sinter funnel dimensions of d = 6.5 cm, h = 1 cm.
- Density of silica = 2.65 g/cm^3, so mass = 33.18 cm^3 * 2.65 g/cm^3 = 87.927 g

7 References

(1) Burchat, A. F.; Chong, J. M.; Nielsen, N. Titration of Alkyllithiums with a Simple Reagent to a Blue Endpoint. *Journal of Organometallic Chemistry* **1997**, *542*, 281–283. https://doi.org/10.1016/S0022-328X(97)00143-5.

(2) Jurrat, M.; Maggi, L.; Lewis, W.; Ball, L. T. Modular Bismacycles for the Selective C–H Arylation of Phenols and Naphthols. *Nat. Chem.* **2020**, *12*, 260–269. https://doi.org/10.1038/s41557-020-0425-4.

(3) Ruffell, K.; Argent, S. P.; Ling, K. B.; Ball, L. T. Bismuth-Mediated α-Arylation of Acidic Diketones with Ortho-Substituted Boronic Acids. *Angewandte Chemie International Edition* **2022**, *61*, e202210840. https://doi.org/10.1002/anie.202210840.

(4) Ruffell, K.; Gallegos, L. C.; Ling, K. B.; Paton, R. S.; Ball, L. T. Umpolung Synthesis of Pyridyl Ethers by BiV-Mediated O-Arylation of Pyridones. *Angewandte Chemie International Edition* **2022**, *61*, e202212873. https://doi.org/10.1002/anie.202212873.

(5) Suzuki, H.; Murafuji, T.; Azuma, N. Synthesis and Reactions of Some New Heterocyclic Bismuth-(III) and -(V) Compounds. 5,10-Dihydrodibenzo[b,e]Bismine and Related Systems. *J. Chem. Soc., Perkin Trans. 1* **1992**, 1593–1600. https://doi.org/10.1039/P19920001593.

(6) Hess, A.; Alandini, N.; Guersoy, Y. C.; Knochel, P. Regioselective Magnesiations of Fluorinated Arenes and Heteroarenes Using Magnesium-Bis-Diisopropylamide (MBDA) in Hydrocarbons. *Angewandte Chemie International Edition* **2022**, *61*, e202206176. https://doi.org/10.1002/anie.202206176.

(7) Blumberg, S.; Martin, S. F. 4-(Phenylazo)Diphenylamine (PDA): A Universal Indicator for the Colorimetric Titration of Strong Bases, Lewis Acids, and Hydride Reducing Agents. *Tetrahedron Letters* **2015**, *56*, 3674–3678. https://doi.org/10.1016/j.tetlet.2015.02.068.

(8) Yu, B.; Liu, A.-H.; He, L.-N.; Li, B.; Diao, Z.-F.; Li, Y.-N. Catalyst-Free Approach for Solvent-Dependent Selective Oxidation of Organic Sulfides with Oxone. *Green Chem.* **2012**, *14*, 957–962. https://doi.org/10.1039/C2GC00027J.

8 Crystallographic Data

Crystallographic data for <u>1-Br</u>

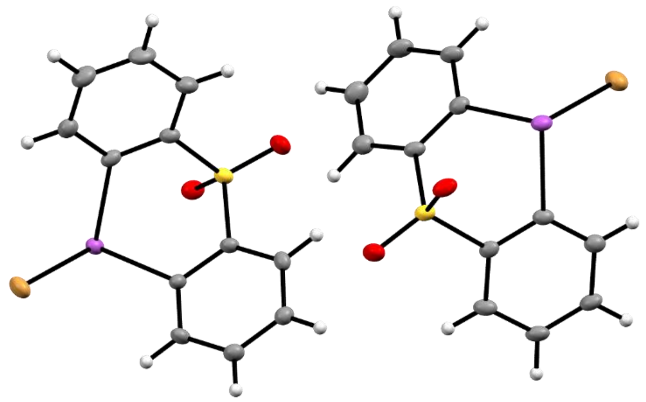

Empirical formula	$C_{12}H_8BiBrO_2S$
Formula weight	505.13
Temperature/K	120.00(10)
Crystal system	monoclinic
Space group	$P2_1/c$
a/Å	15.3959(3)
b/Å	19.2379(4)
c/Å	8.6034(2)
α/°	90
β/°	97.324(2)
γ/°	90
Volume/Å^3	2527.41(9)
Z	8
ρ_{calc}g/cm^3	2.655
μ/mm^{-1}	17.264
F(000)	1840.0
Crystal size/mm^3	0.298 × 0.202 × 0.182
Radiation	Mo Kα (λ = 0.71073)
2Θ range for data collection/°	6.68 to 70.298
Index ranges	$-24 \leq h \leq 24$, $-31 \leq k \leq 30$, $-13 \leq l \leq 13$
Reflections collected	81982
Independent reflections	10787 [R_{int} = 0.0652, R_{sigma} = 0.0393]
Data/restraints/parameters	10787/0/307
Goodness-of-fit on F^2	1.087
Final R indexes [I>=2σ (I)]	R_1 = 0.0306, wR_2 = 0.0500
Final R indexes [all data]	R_1 = 0.0481, wR_2 = 0.0547
Largest diff. peak/hole / e Å^{-3}	1.51/-1.17

9 NMR Spectra

1-Br – ^{1}H NMR (400 MHz, $CDCl_3$):

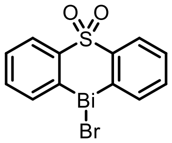

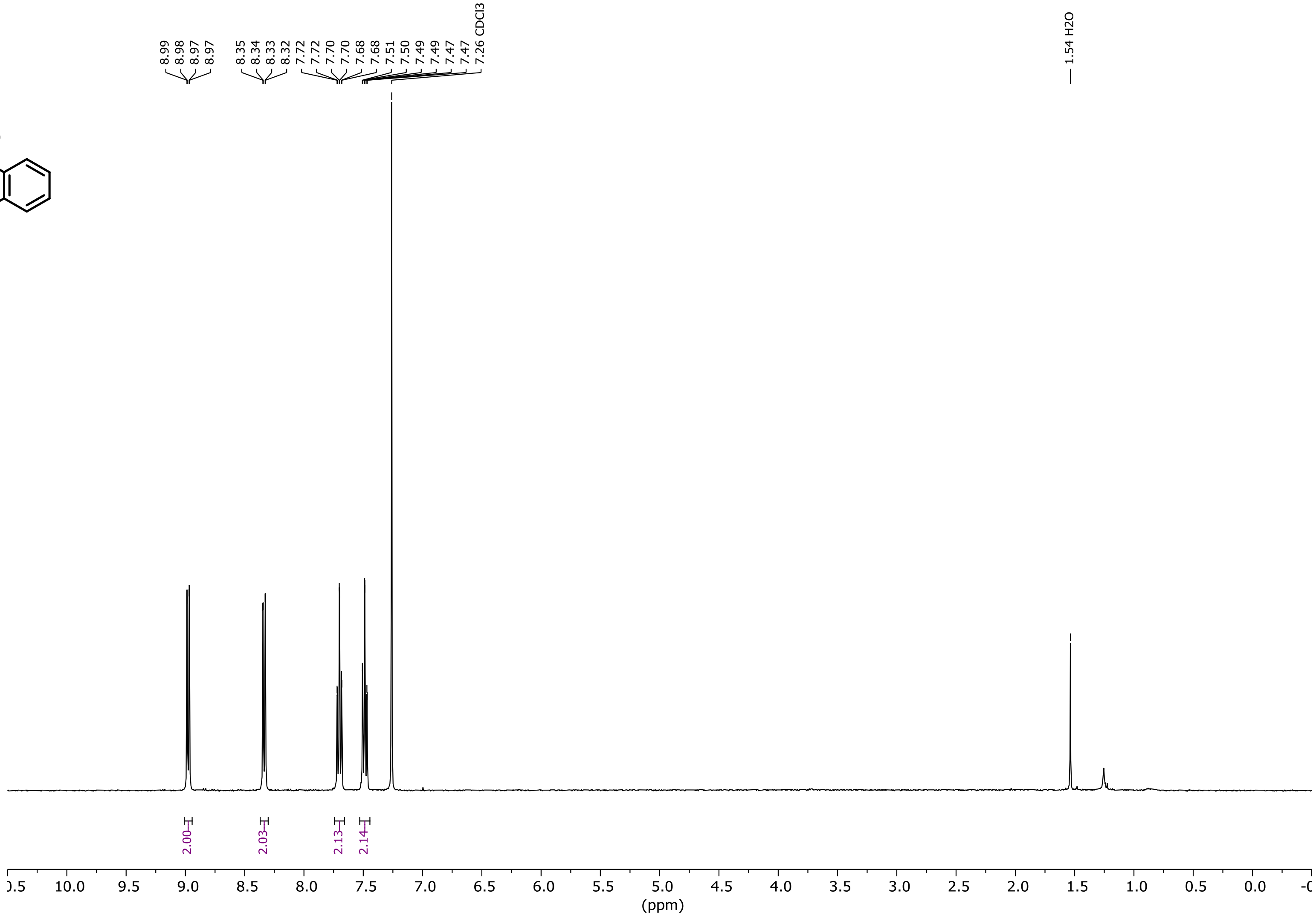

1-Br – $^{13}C\{^{1}H\}$ NMR (101 MHz, $CDCl_3$):

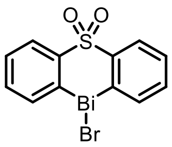

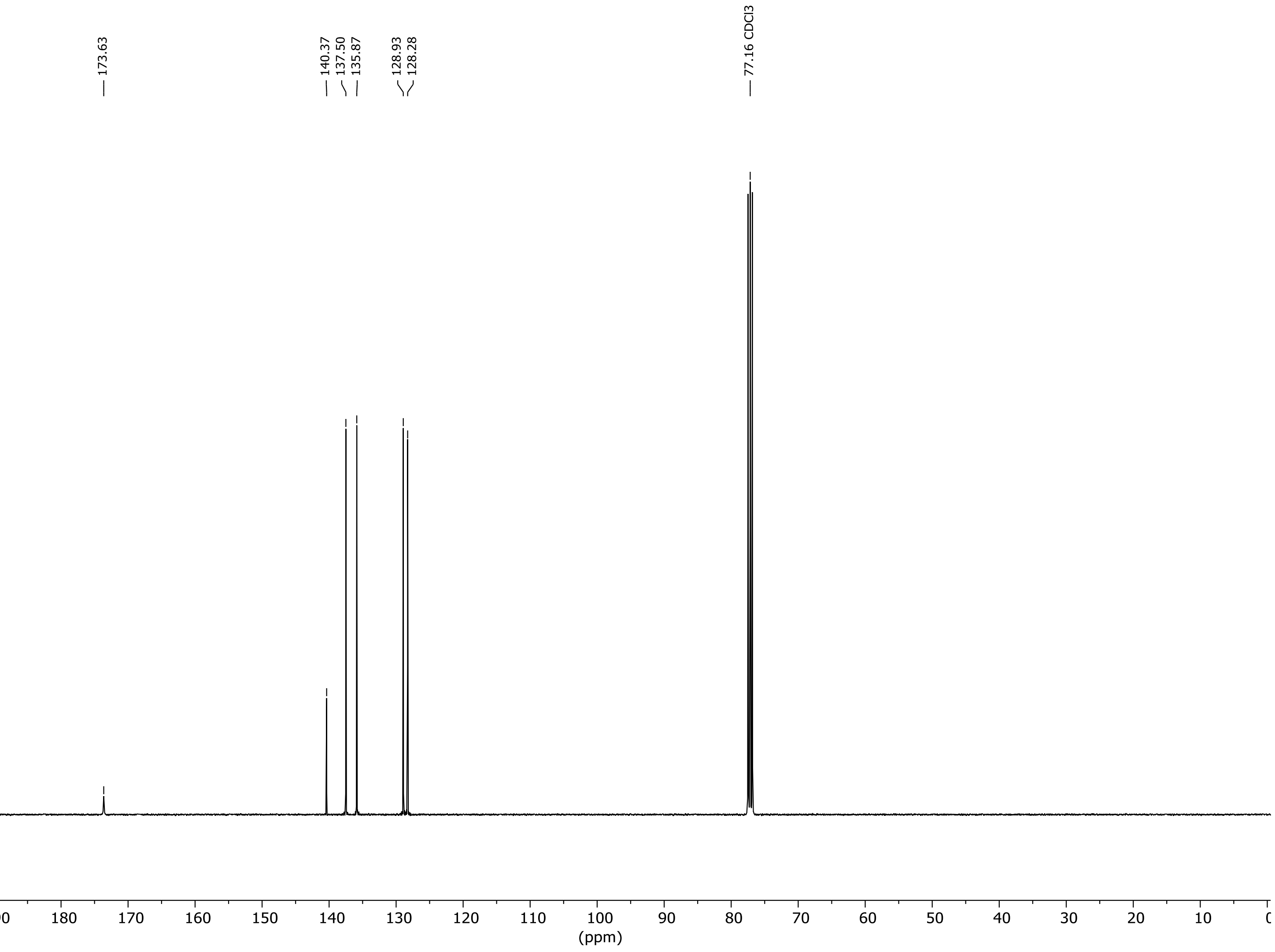

1-Cl – ^{1}H NMR (400 MHz, $CDCl_3$):

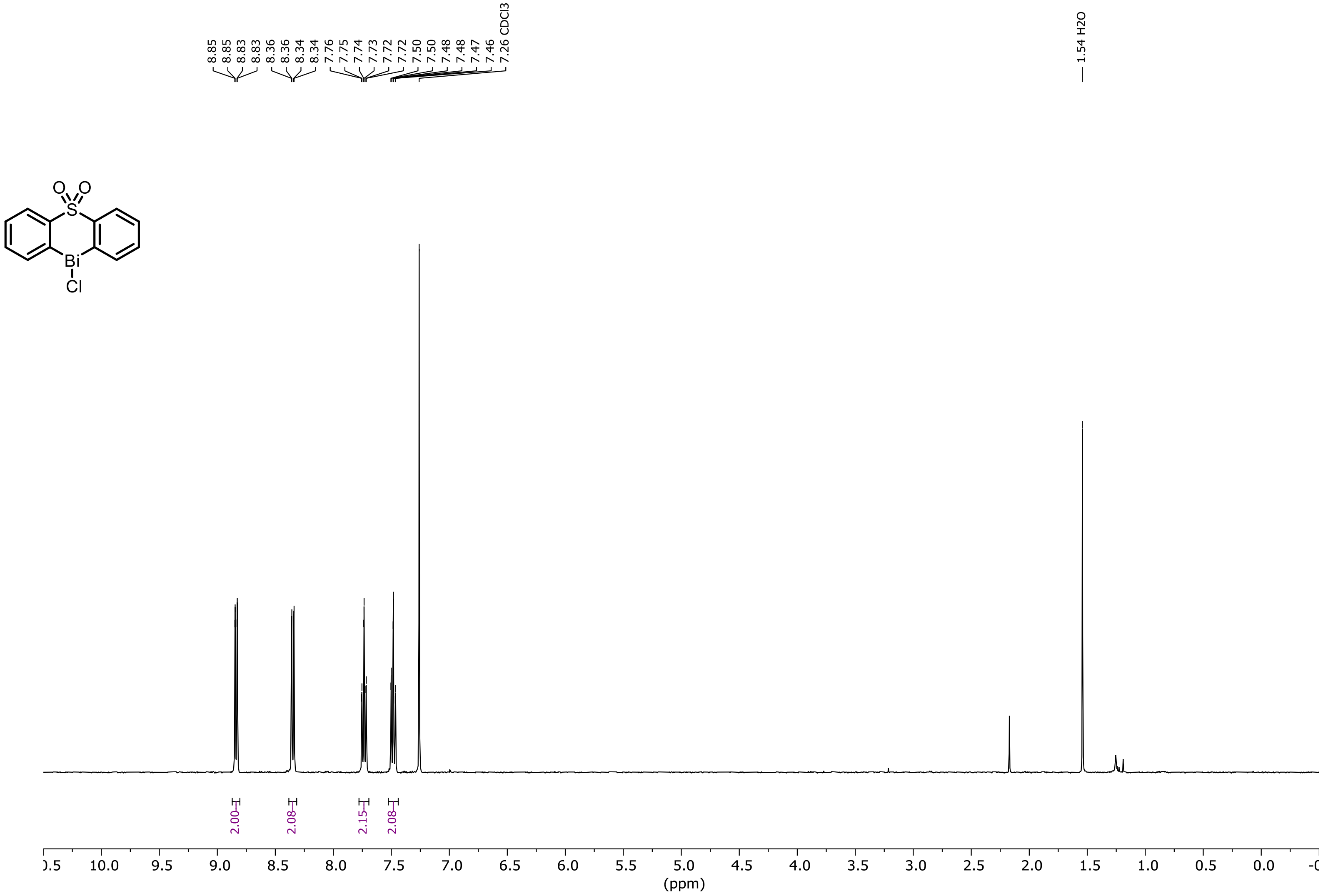

1-Cl – $^{13}C\{^1H\}$ NMR (101 MHz, $CDCl_3$):

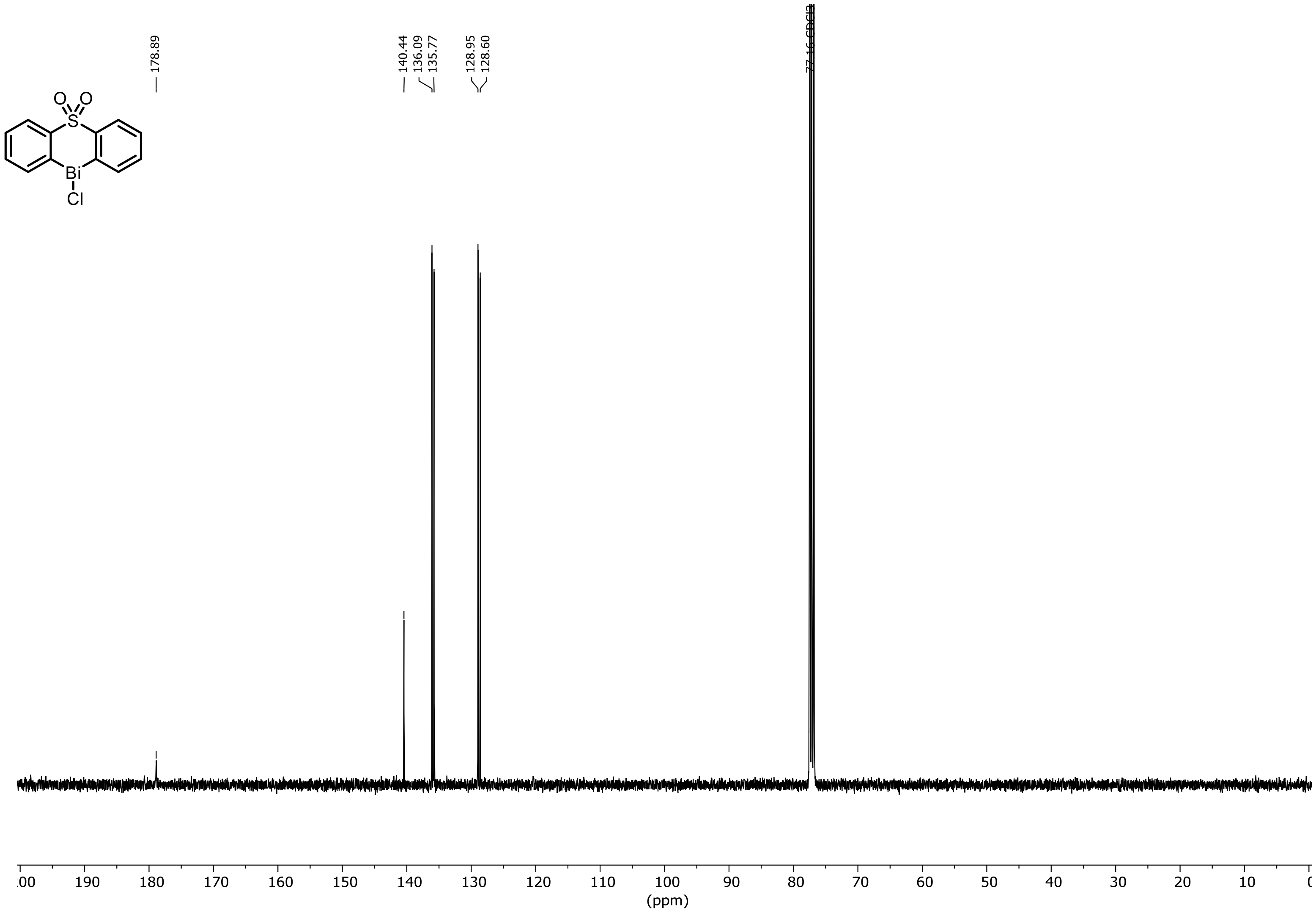

2a – ^{1}H NMR (400 MHz, CDCl₃):

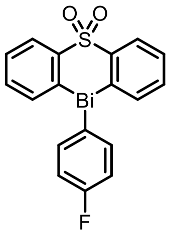

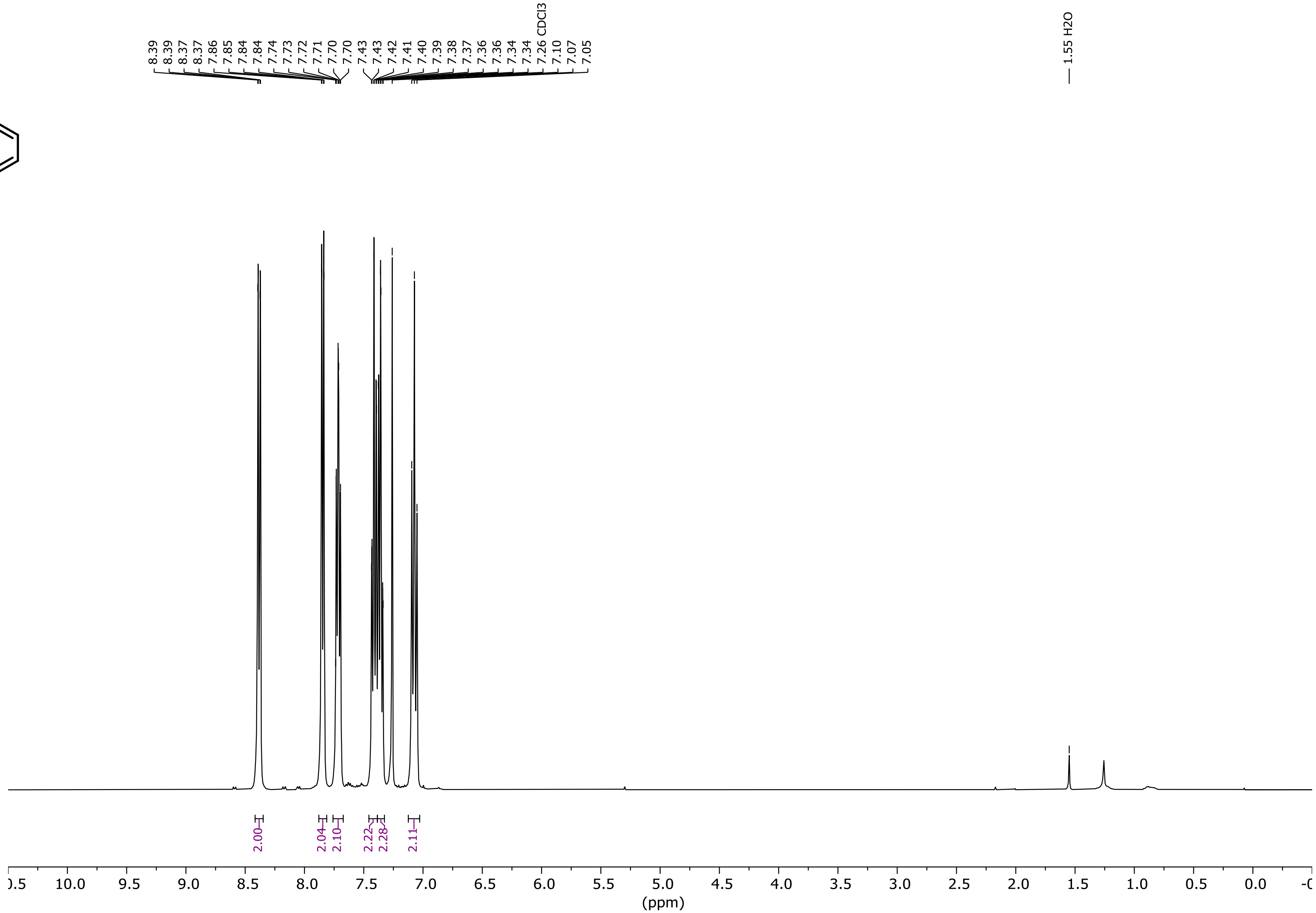

2a – $^{13}C\{^{1}H\}$ NMR (101 MHz, CDCl₃):

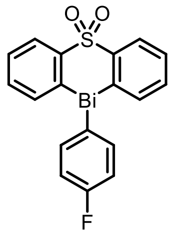

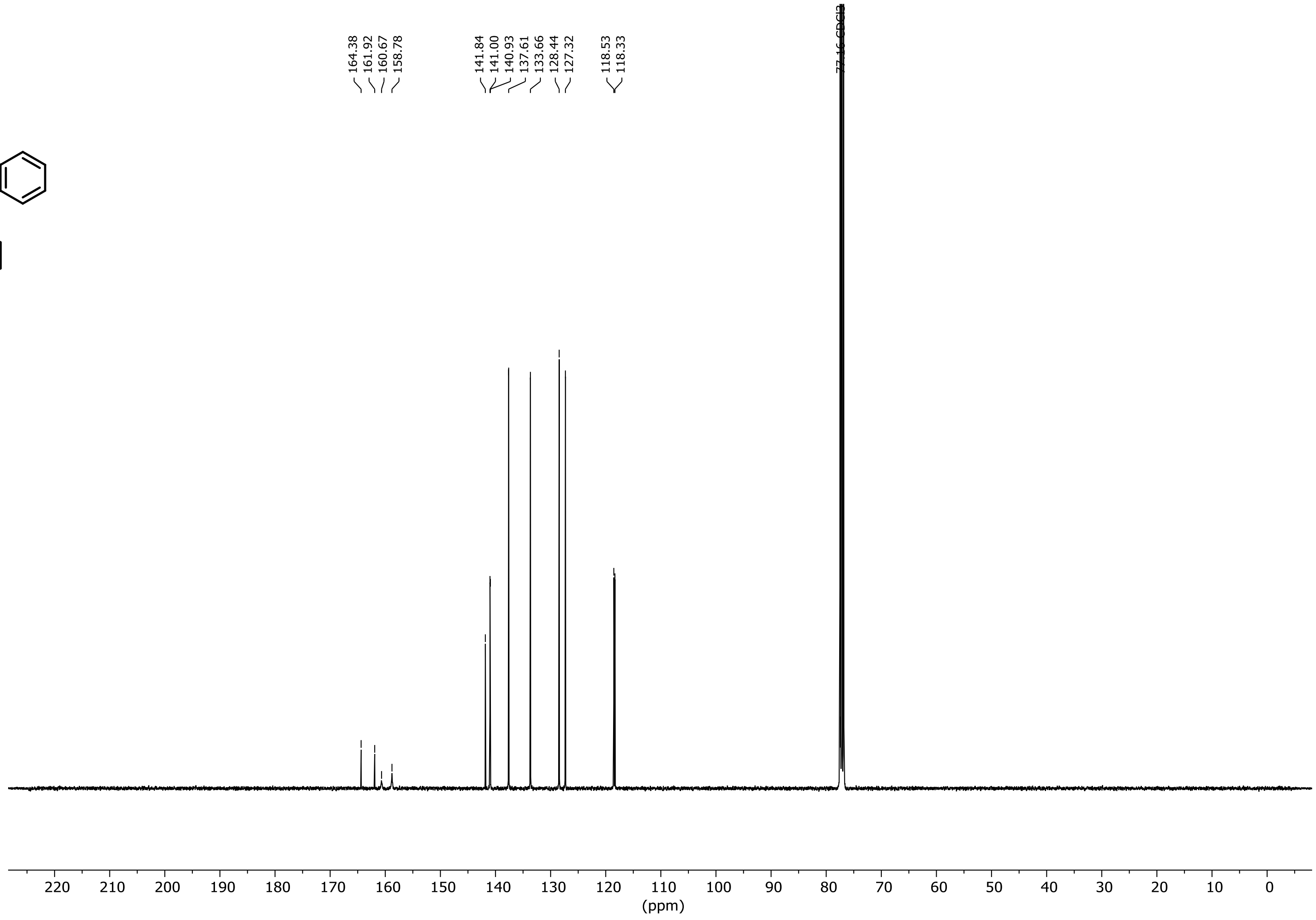

2a – ^{19}F NMR (377 MHz, $CDCl_3$):

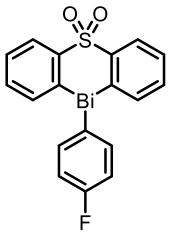

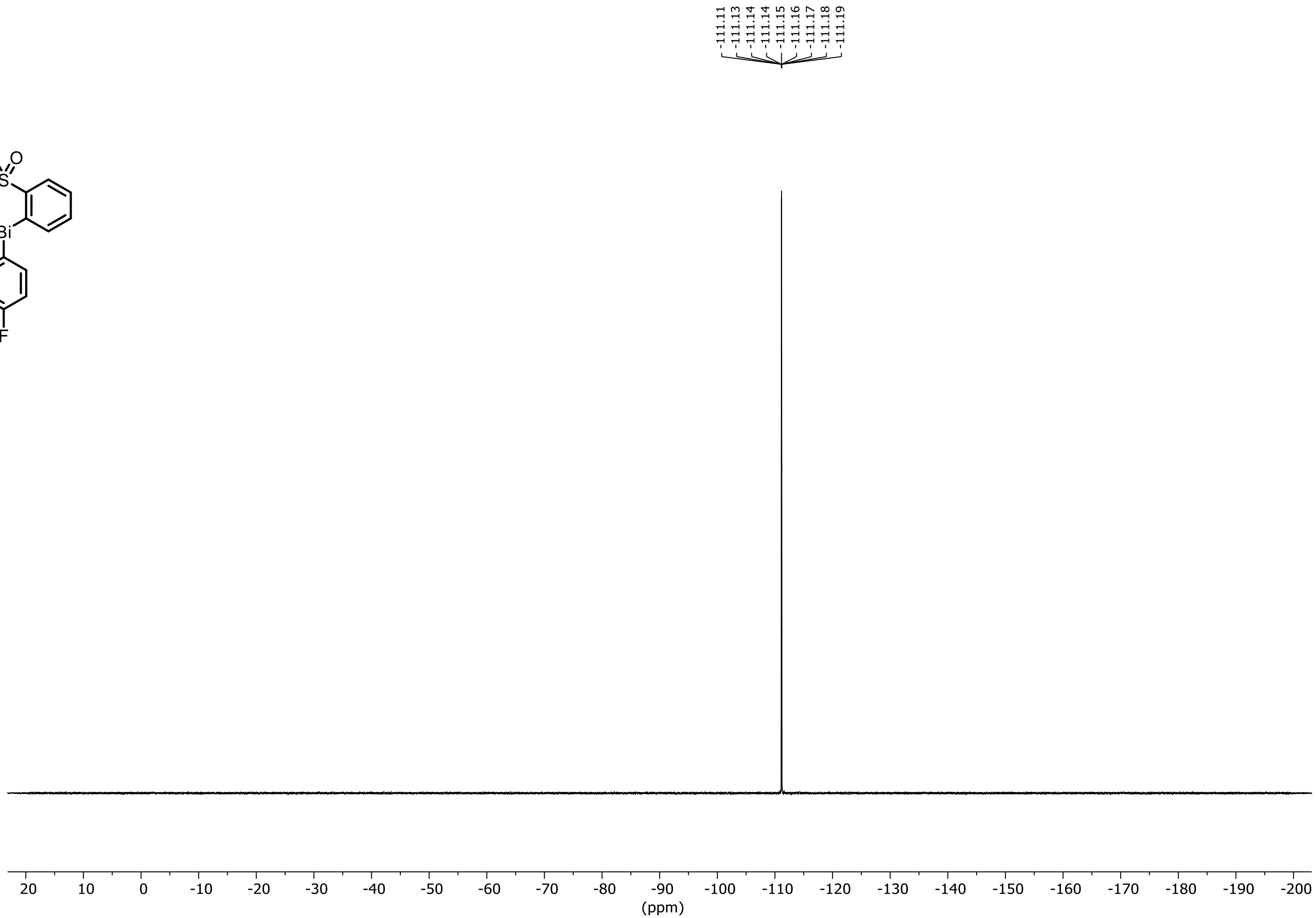

2b – ^{1}H NMR (400 MHz, $CDCl_3$):

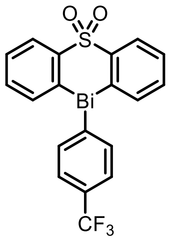

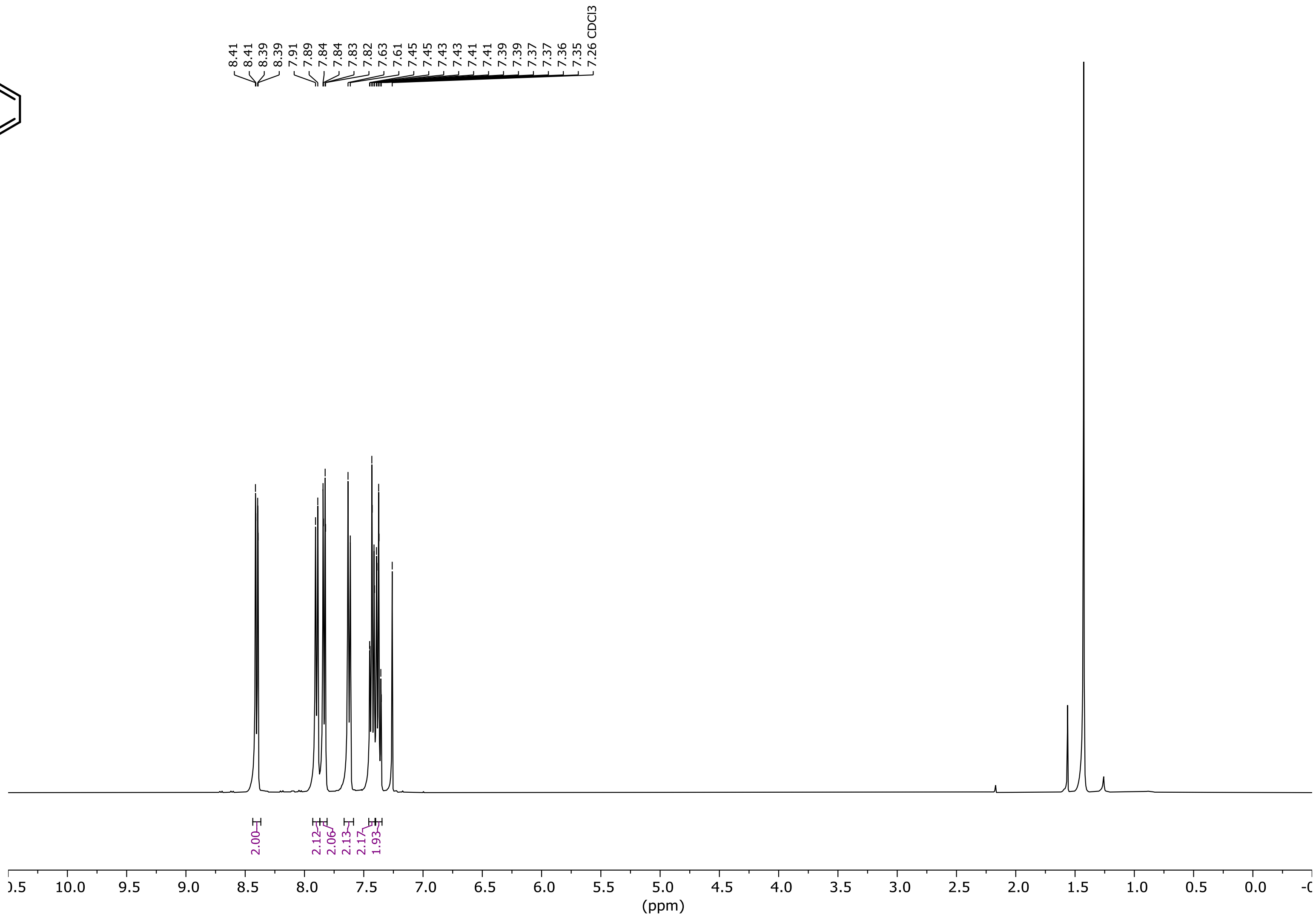

2b – $^{13}C\{^{1}H\}$ NMR (101 MHz, $CDCl_3$):

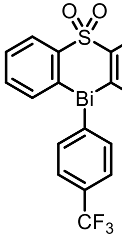

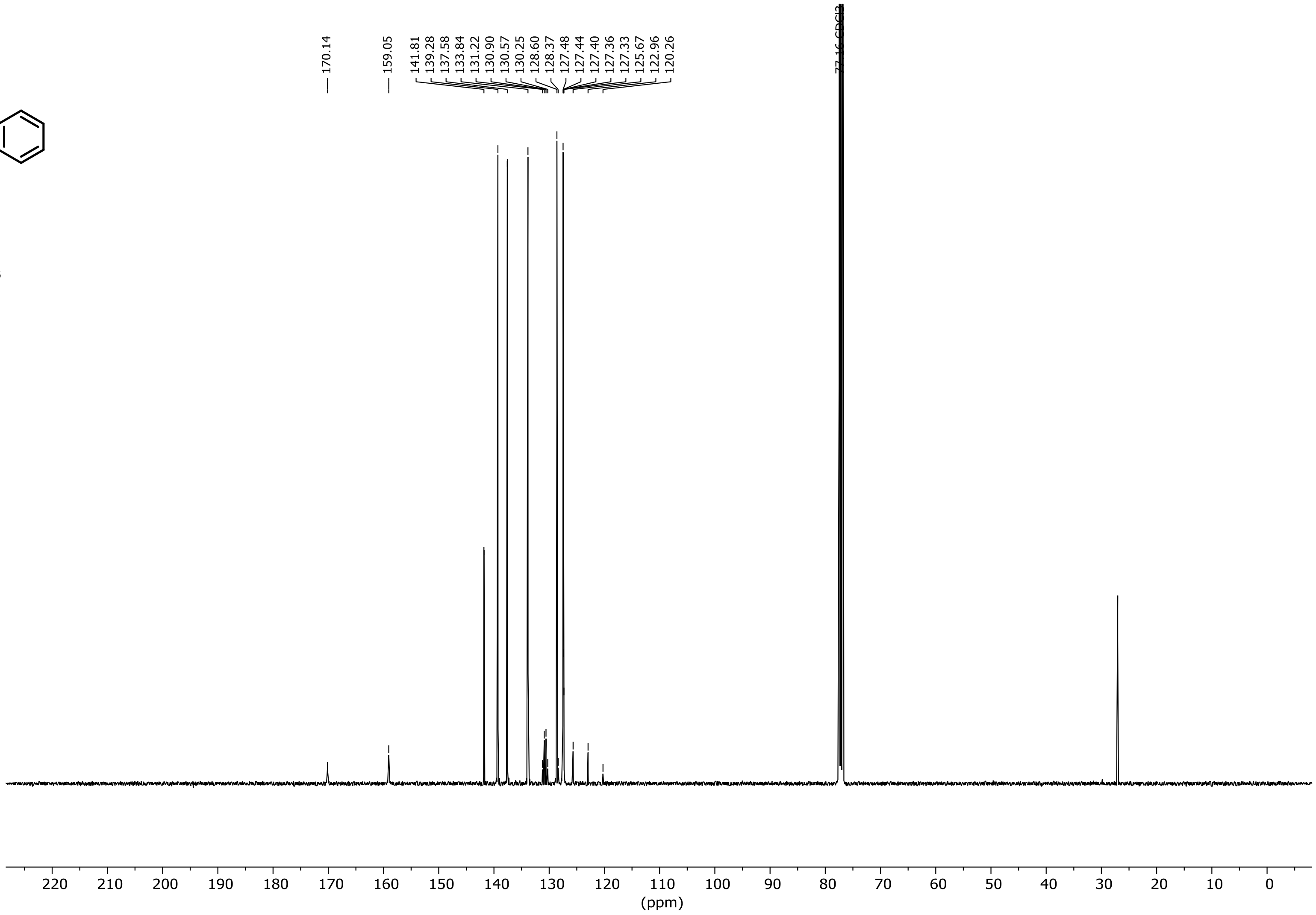

2b – ^{19}F NMR (377 MHz, $CDCl_3$):

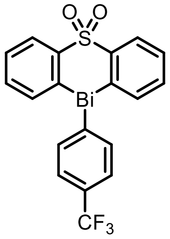

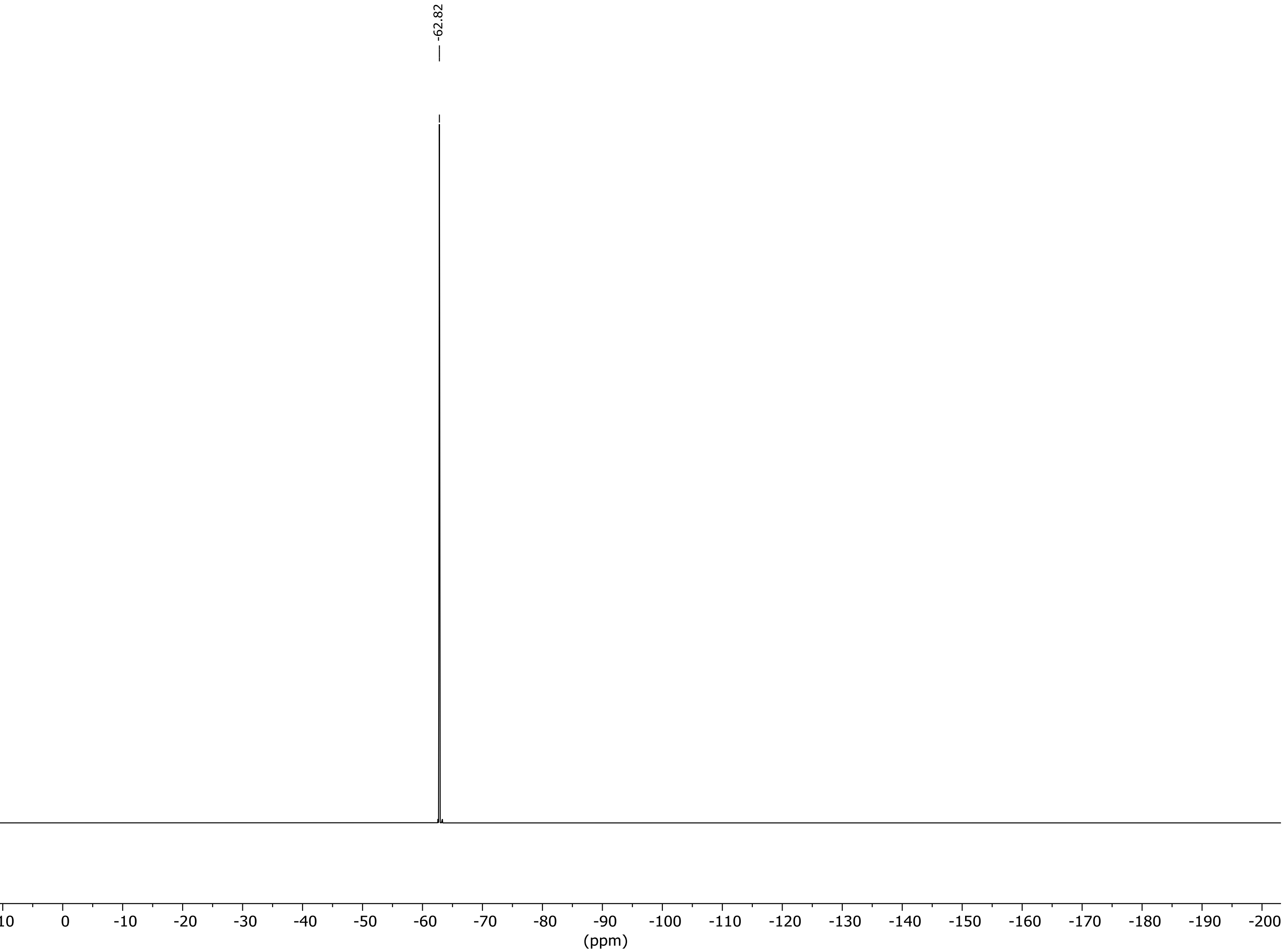

2c – ^{1}H NMR (400 MHz, CDCl₃):

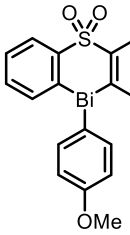

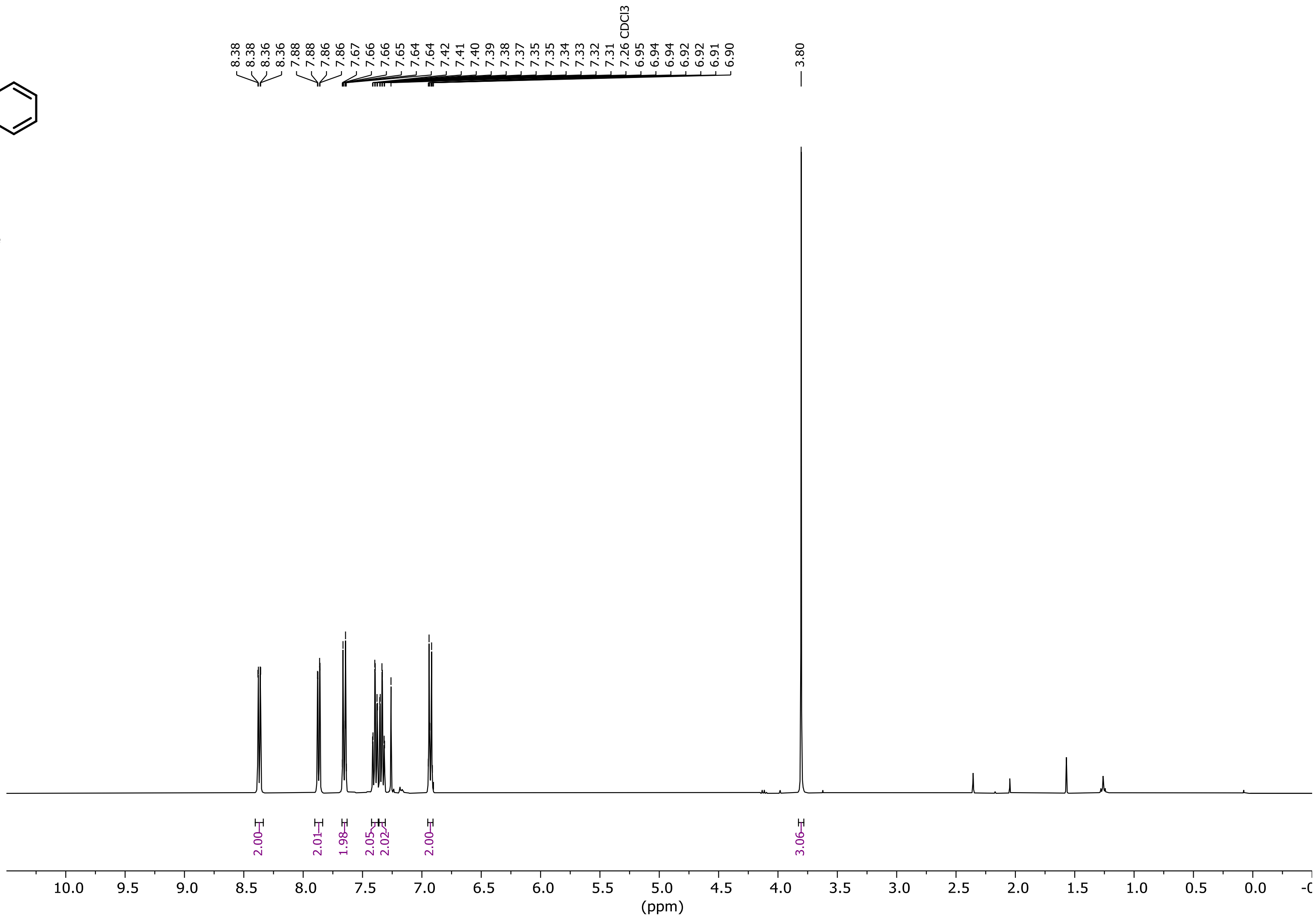

2c – $^{13}C\{^1H\}$ NMR (101 MHz, $CDCl_3$):

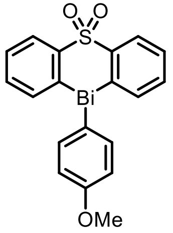

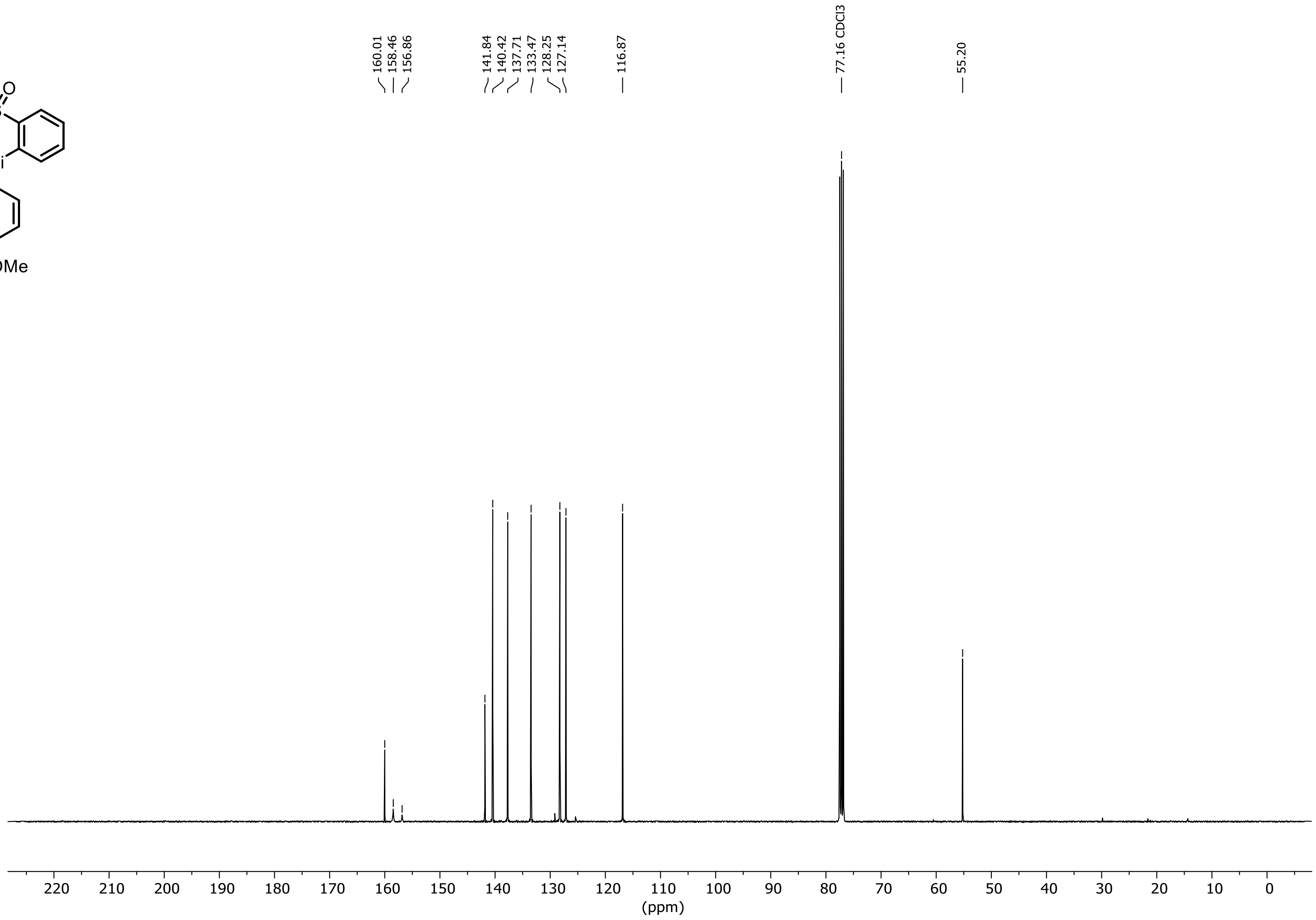

2d – ^{1}H NMR (400 MHz, $CDCl_3$):

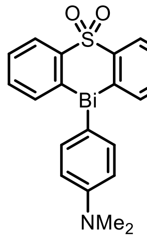

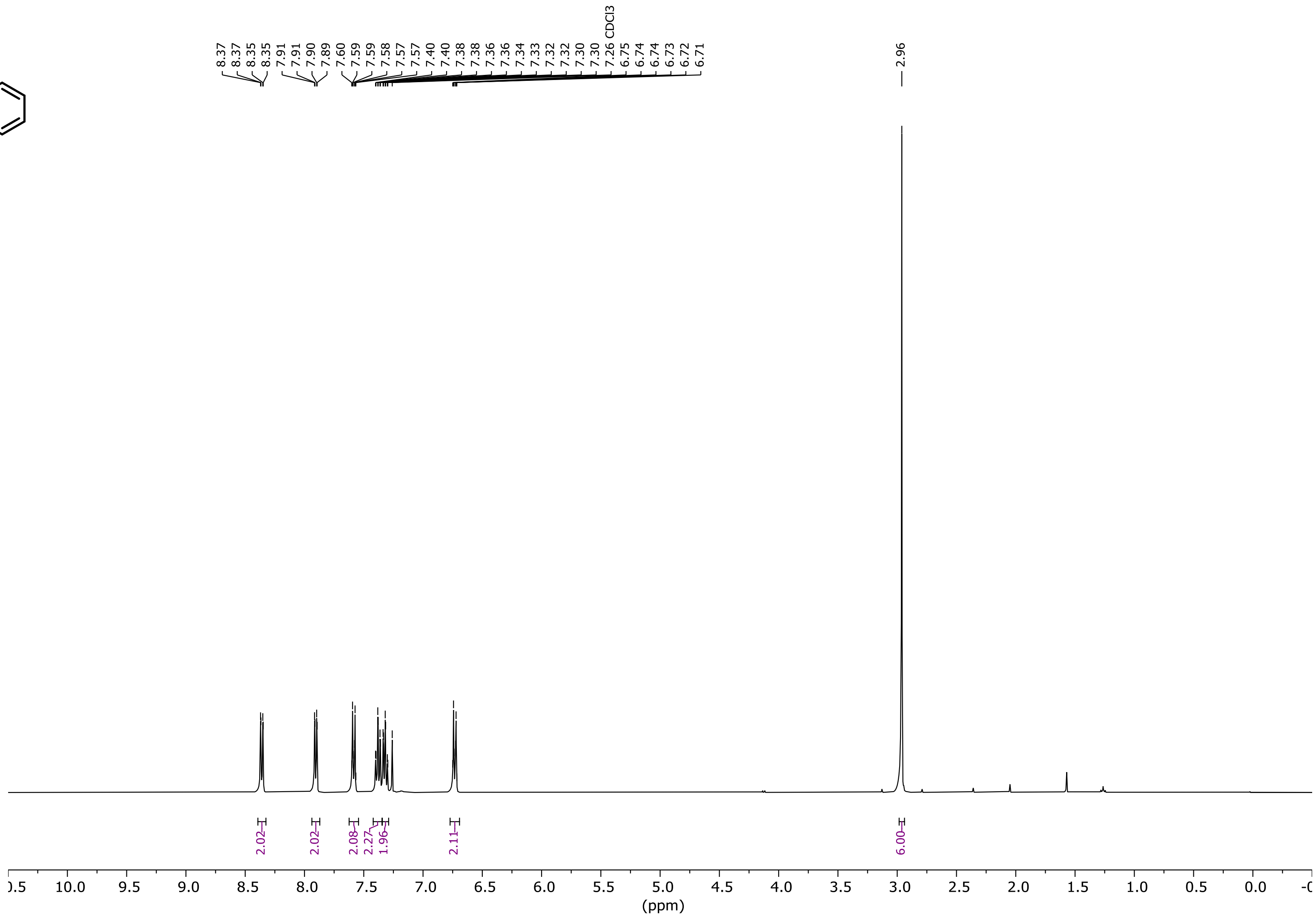

2d – $^{13}C\{^1H\}$ NMR (101 MHz, $CDCl_3$):

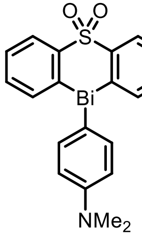

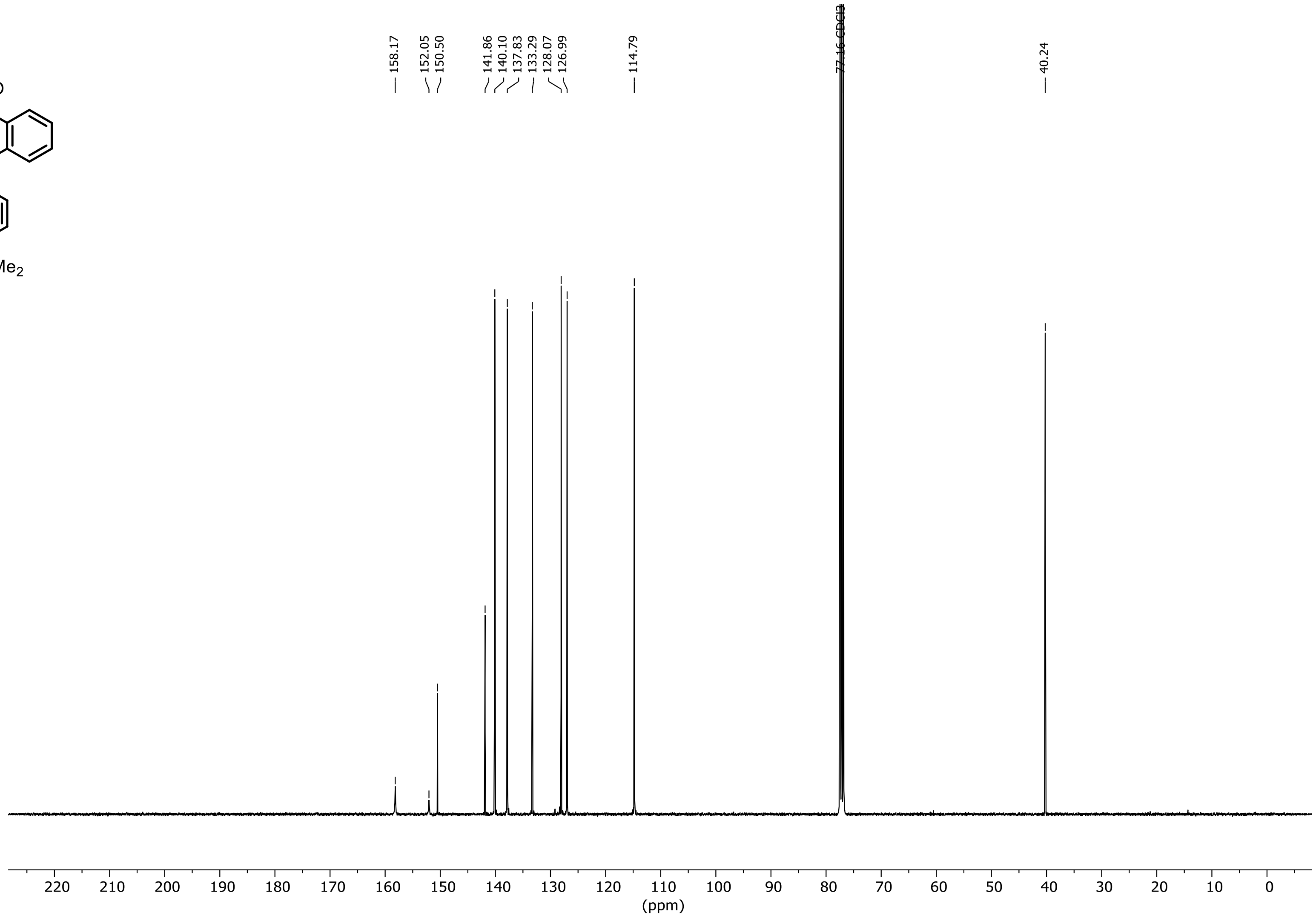

2e – ^{1}H NMR (400 MHz, $CDCl_3$):

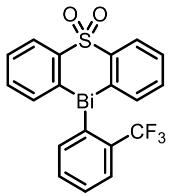

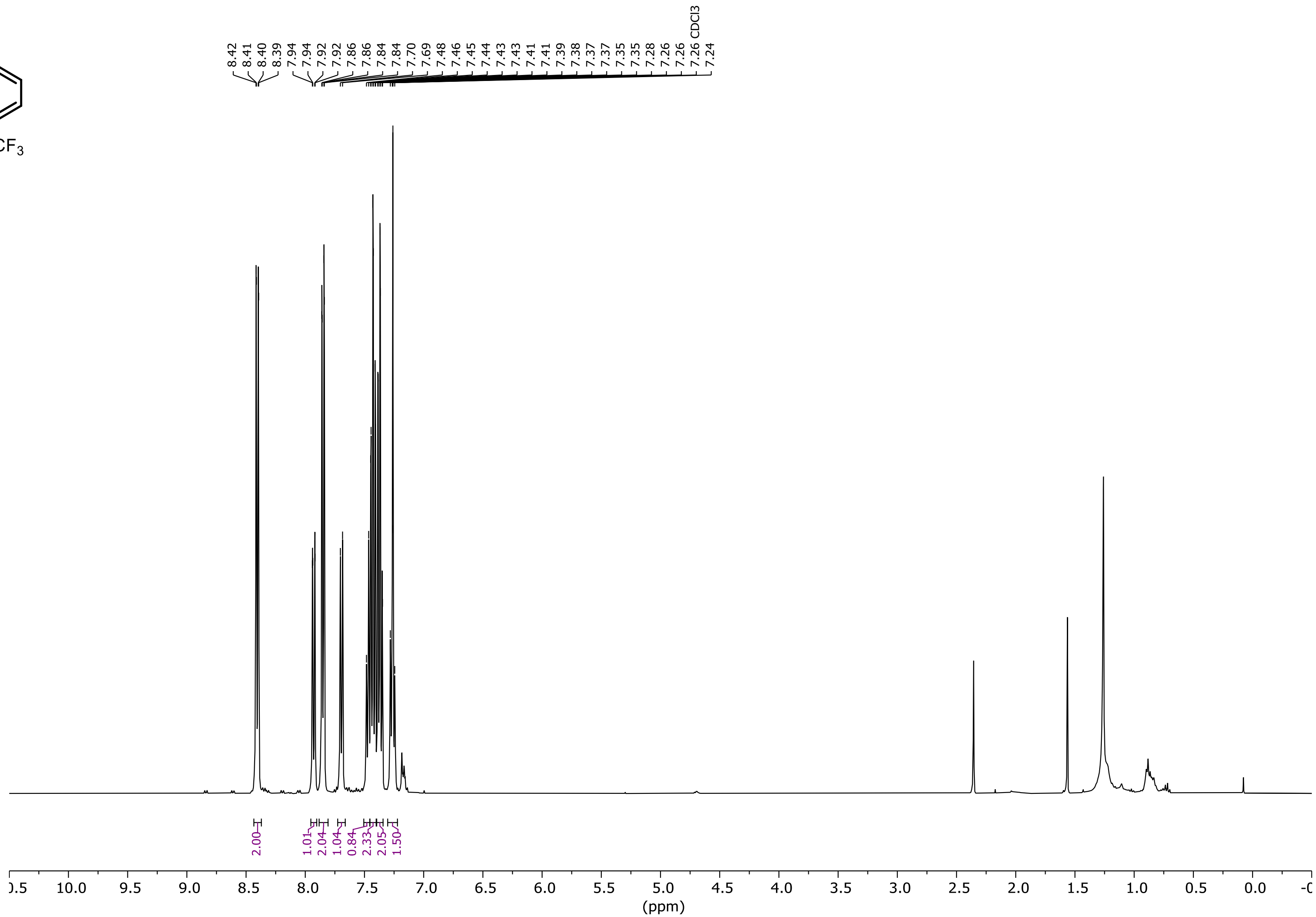

2e – $^{13}C\{^1H\}$ NMR (101 MHz, CDCl₃):

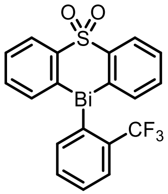

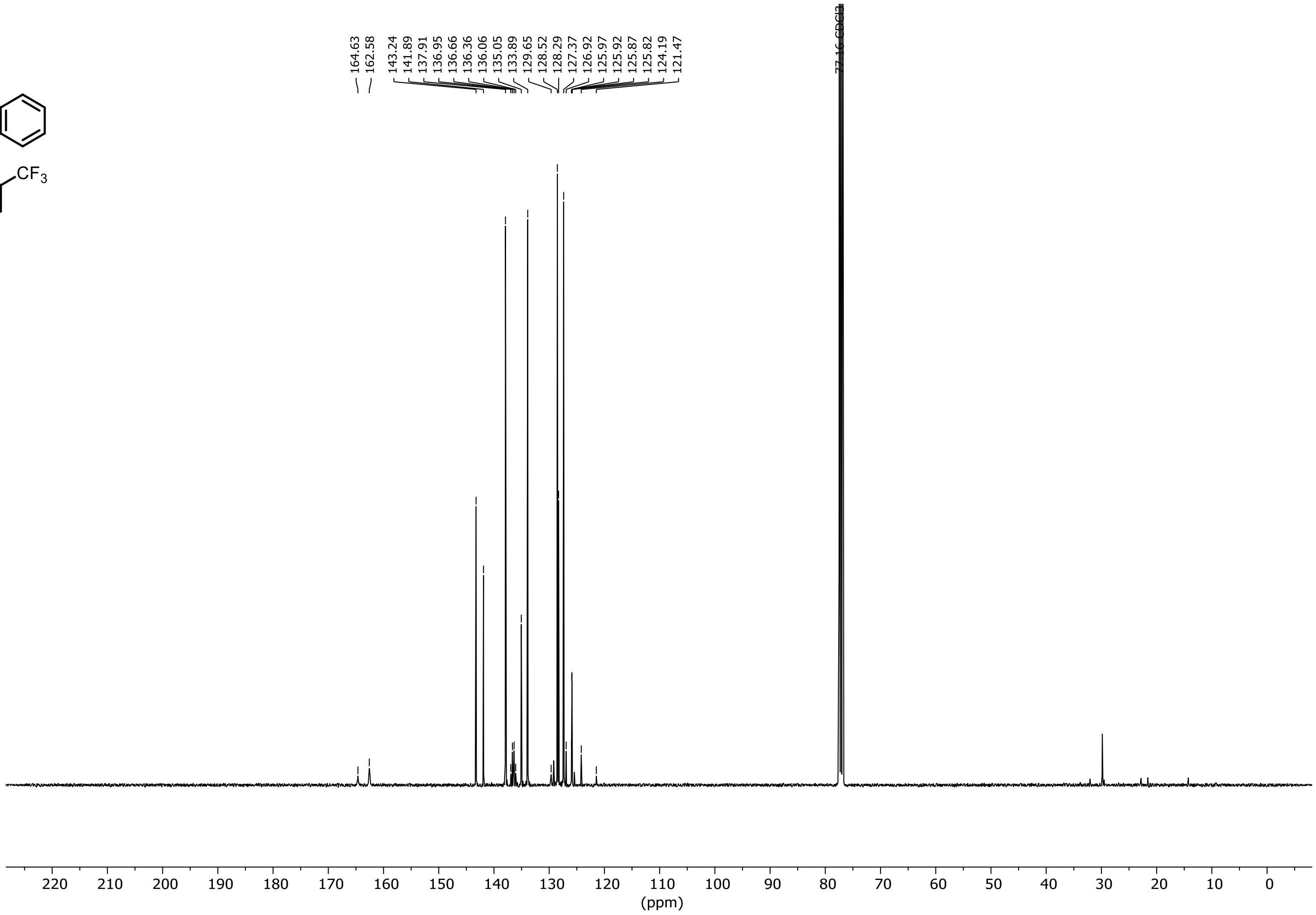

2e – ^{19}F NMR (377 MHz, $CDCl_3$):

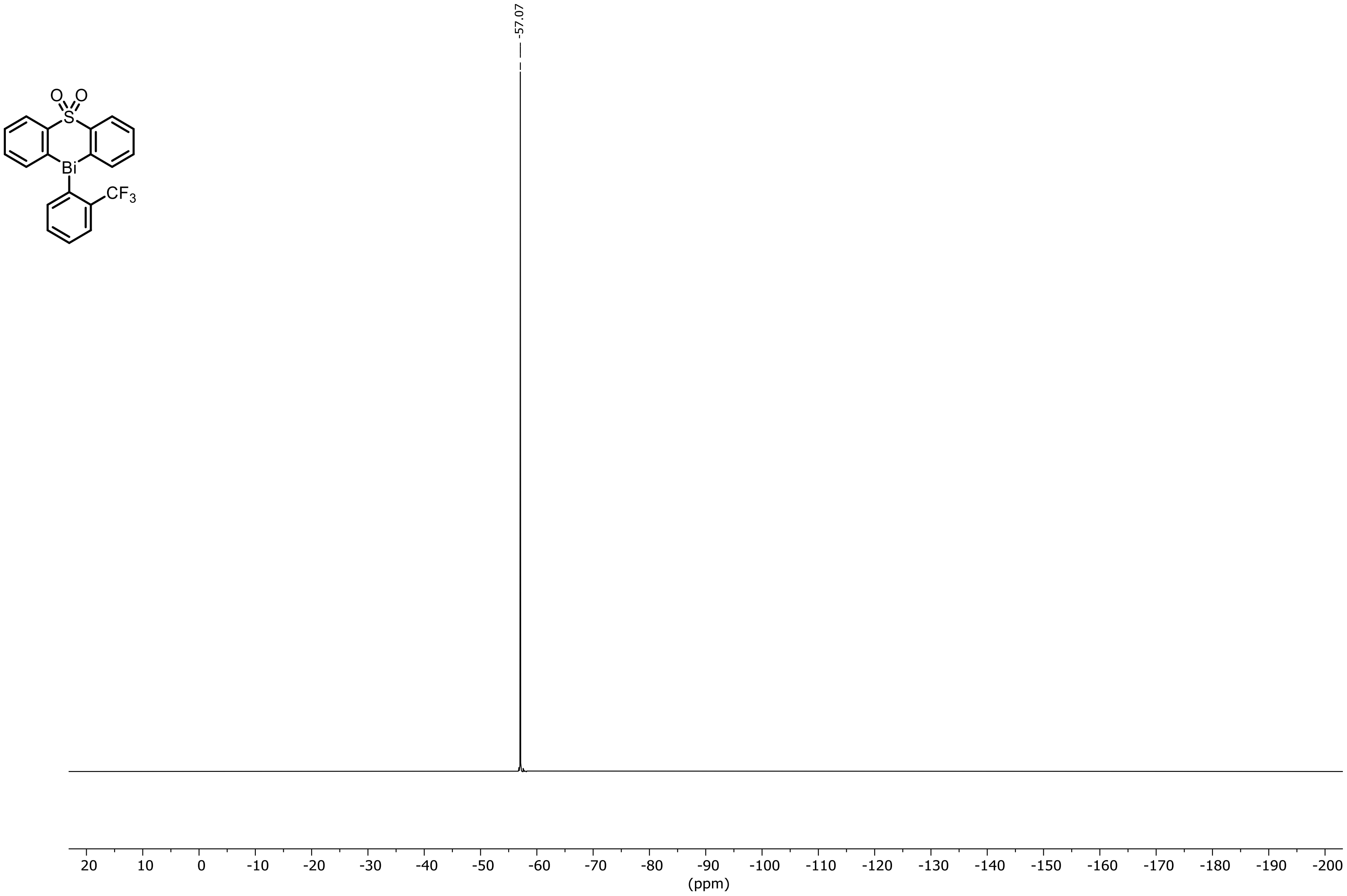

2f – ^{1}H NMR (400 MHz, CDCl$_3$):

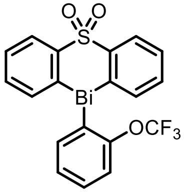

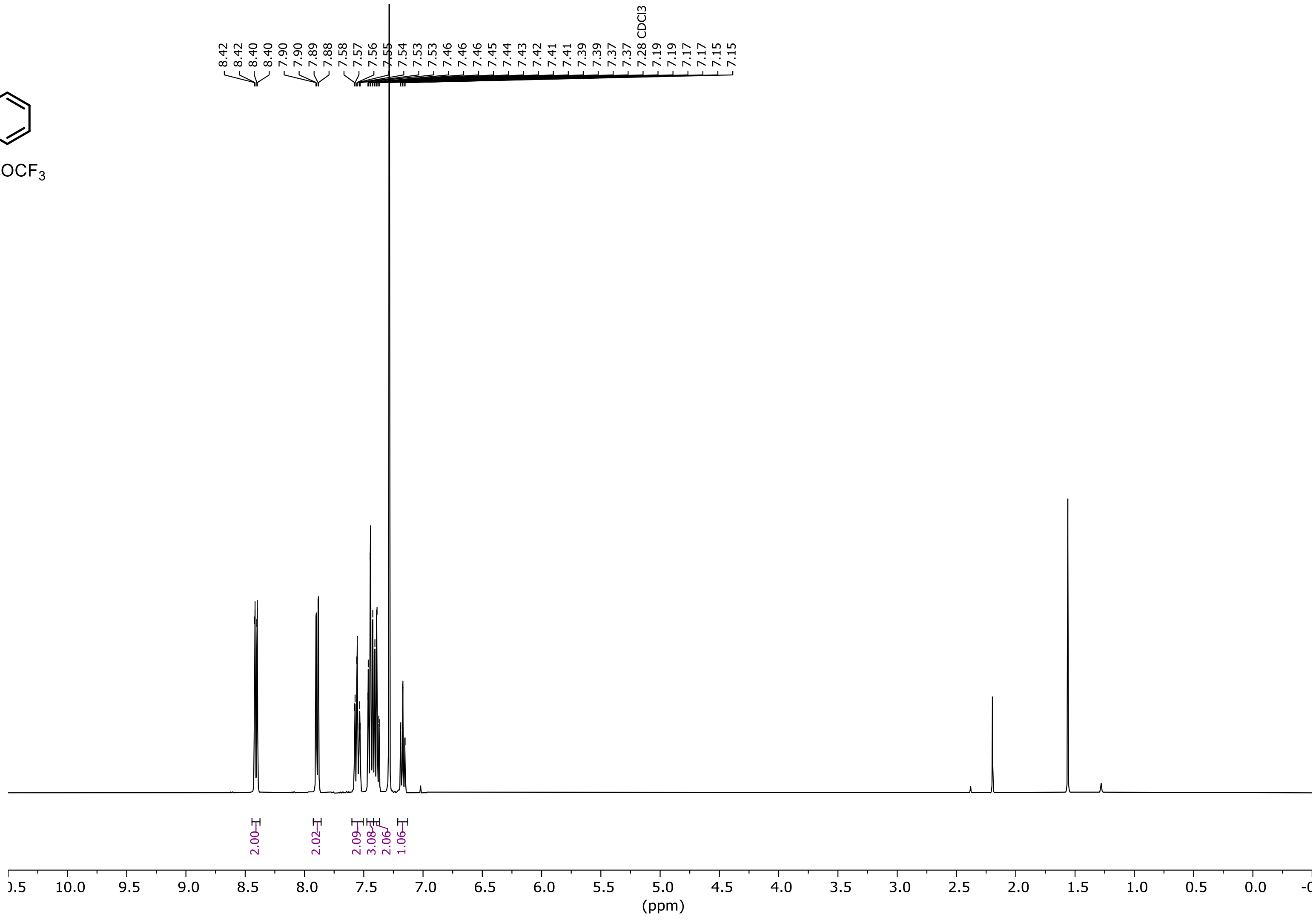

2f – $^{13}C\{^{1}H\}$ NMR (126 MHz, CDCl$_3$):

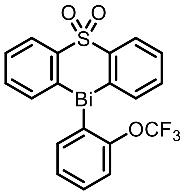

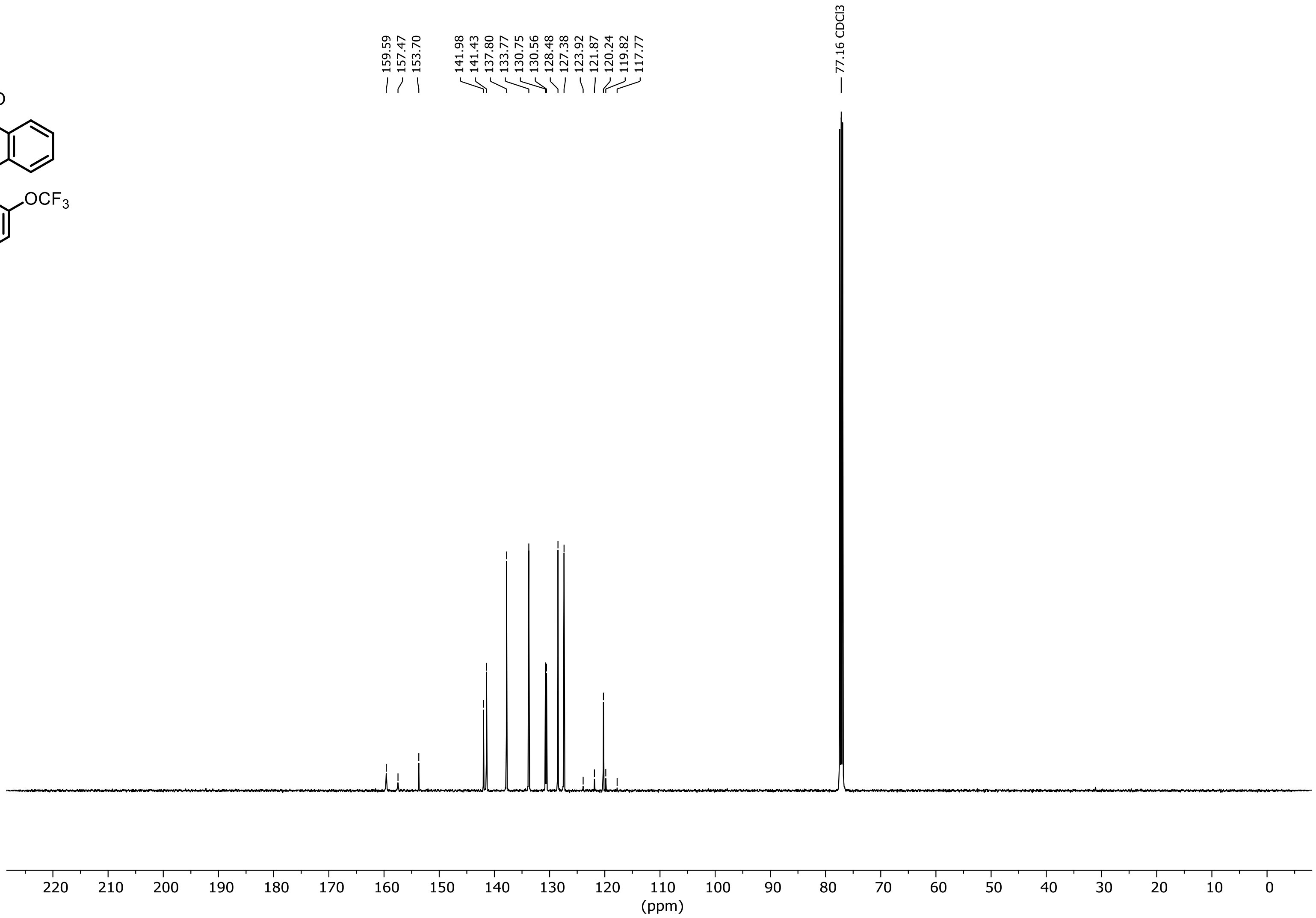

2f – ^{19}F NMR (377 MHz, $CDCl_3$):

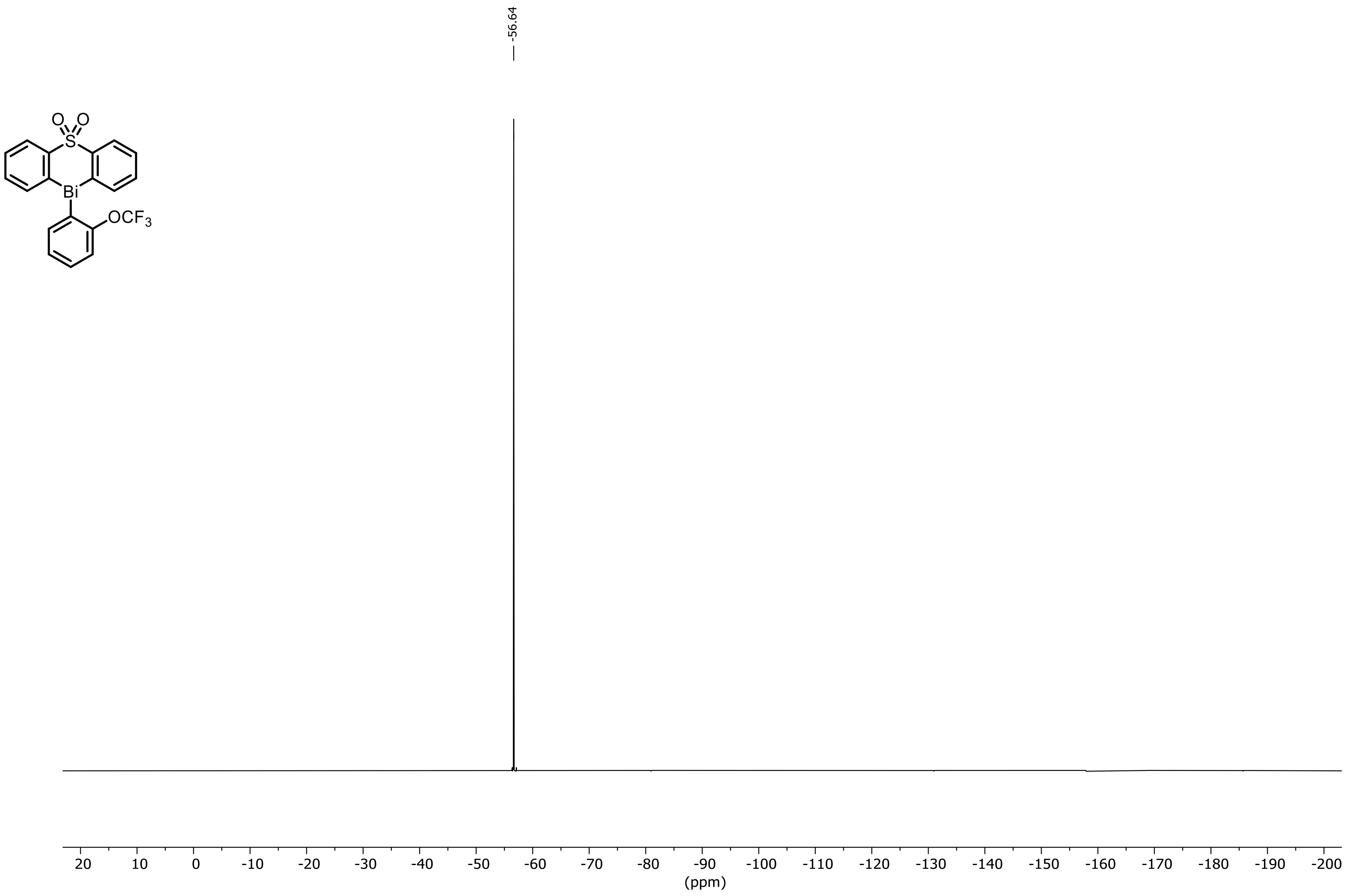

2g – ^{1}H NMR (400 MHz, CDCl₃):

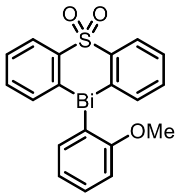

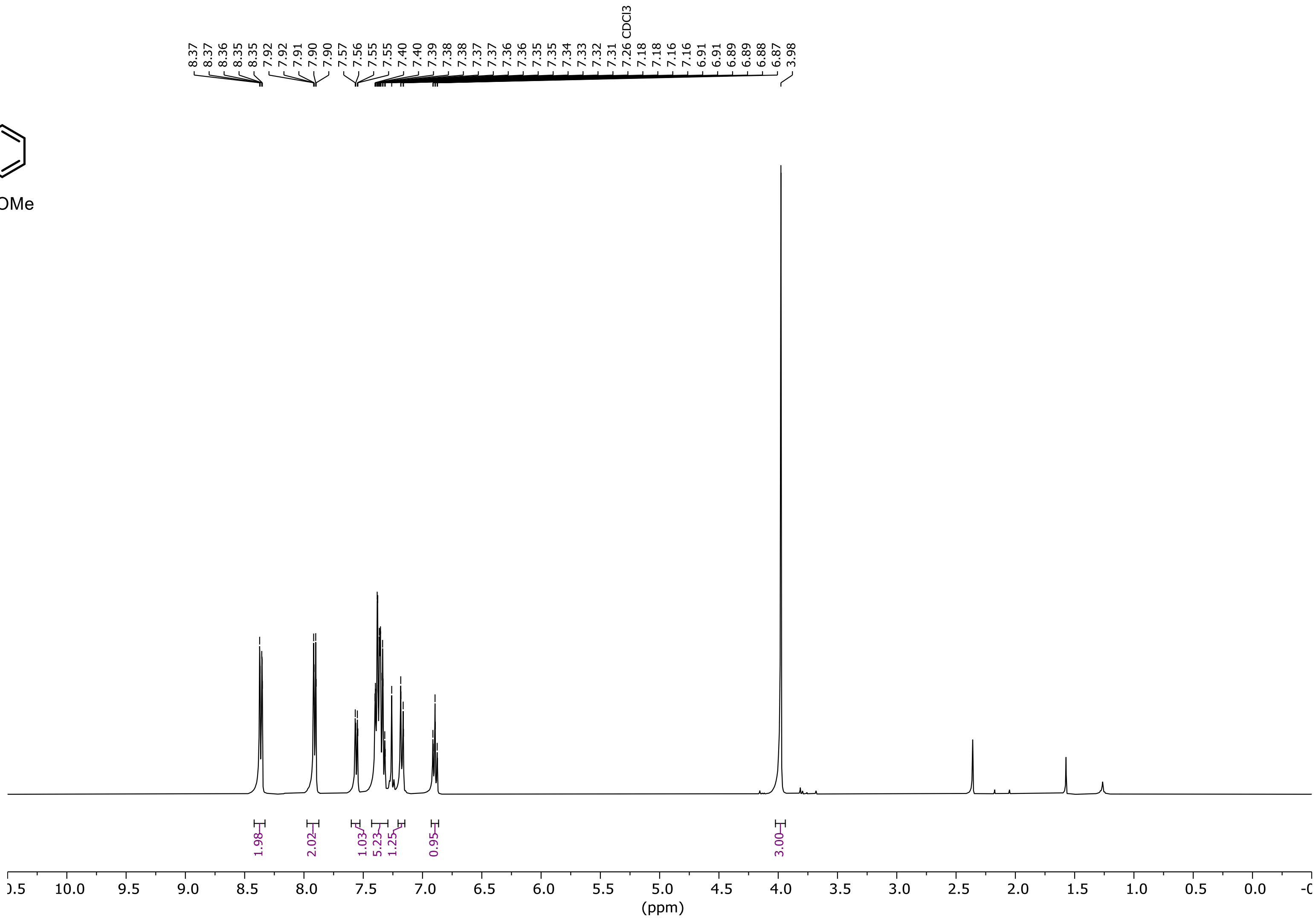

2g – $^{13}C\{^{1}H\}$ NMR (101 MHz, $CDCl_3$):

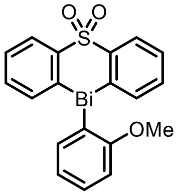

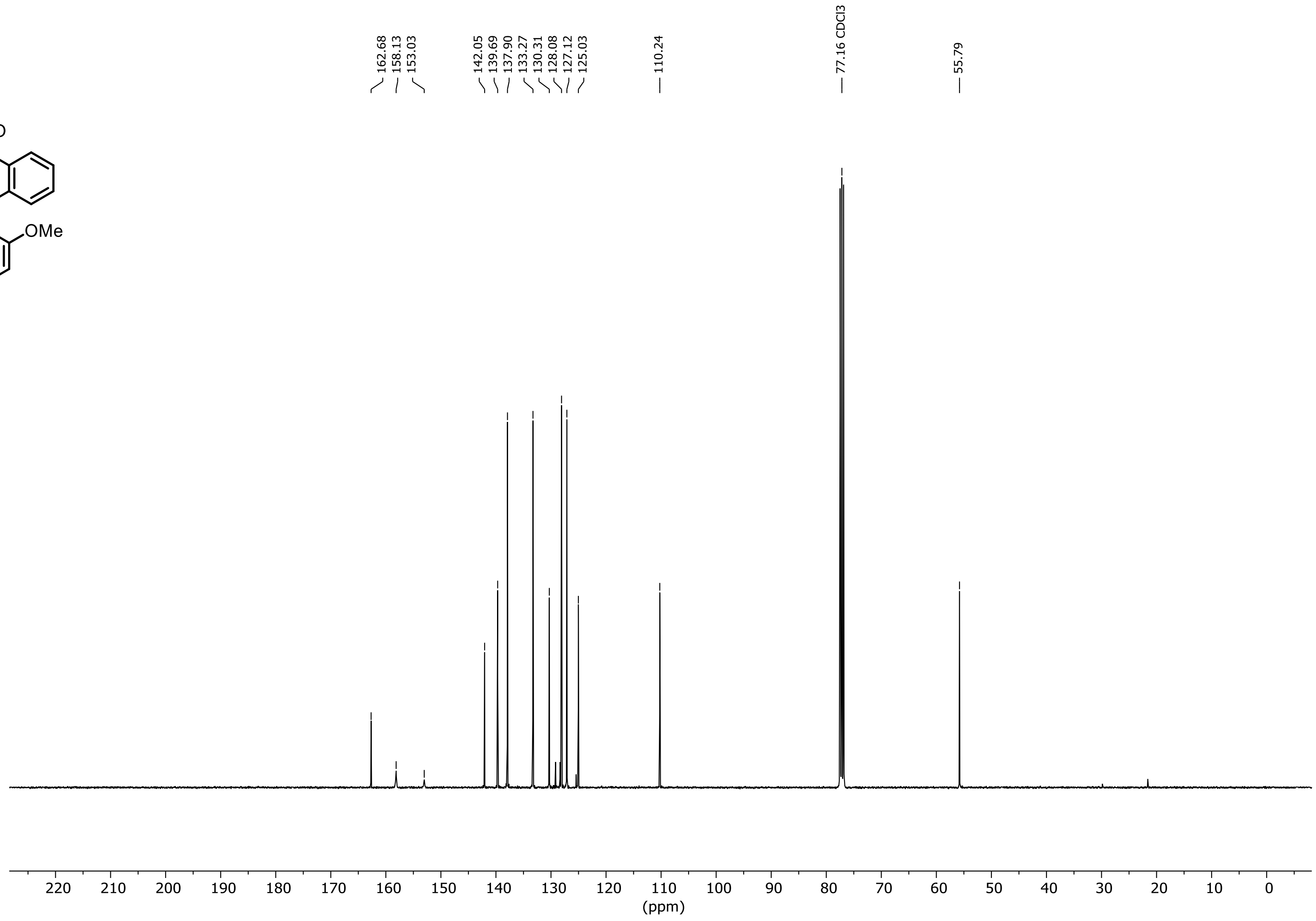

2h – ^{1}H NMR (400 MHz, CDCl₃):

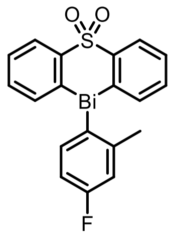

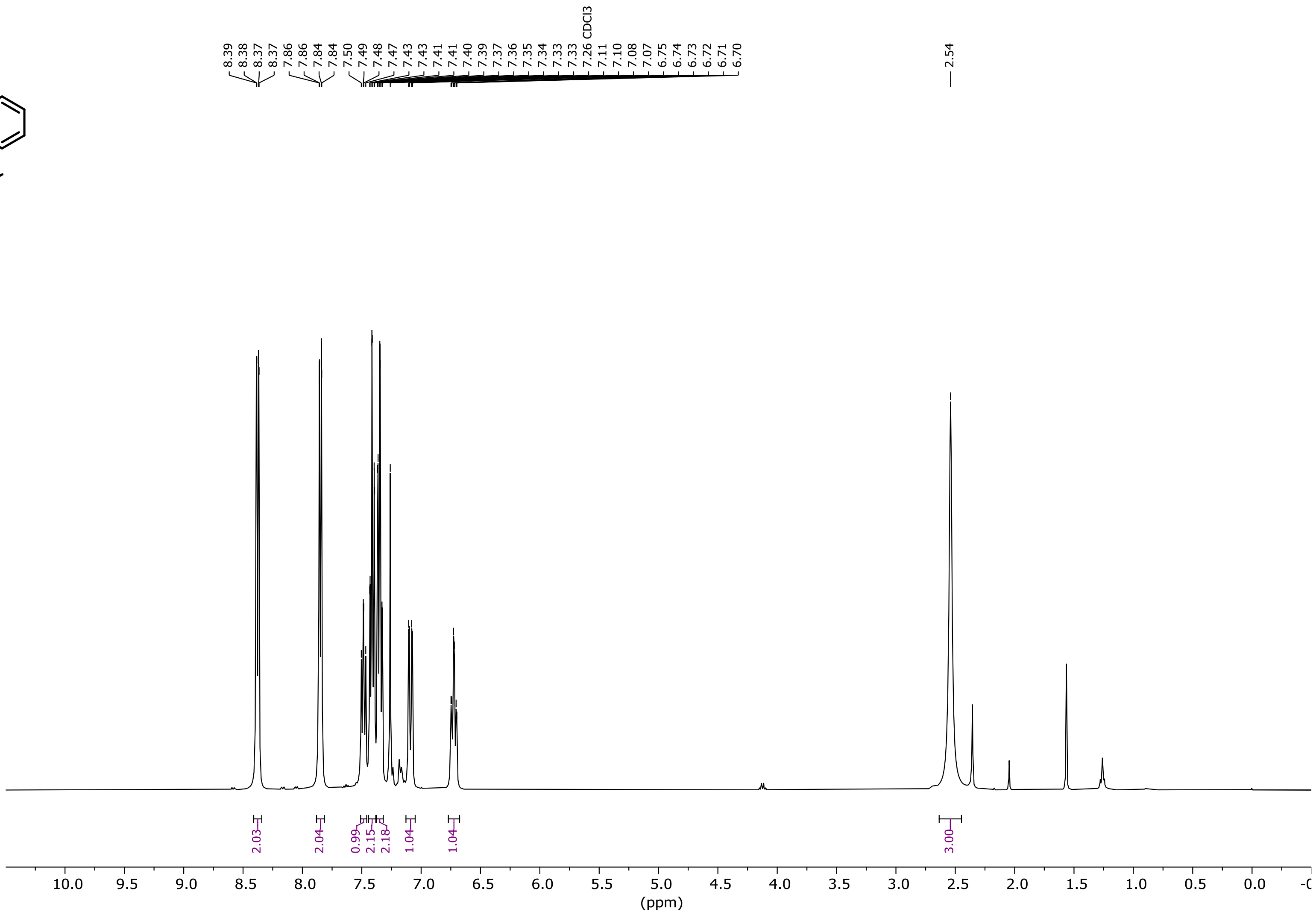

2h – $^{13}C\{^{1}H\}$ NMR (101 MHz, CDCl₃):

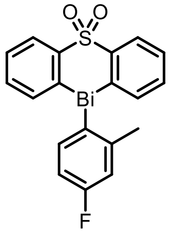

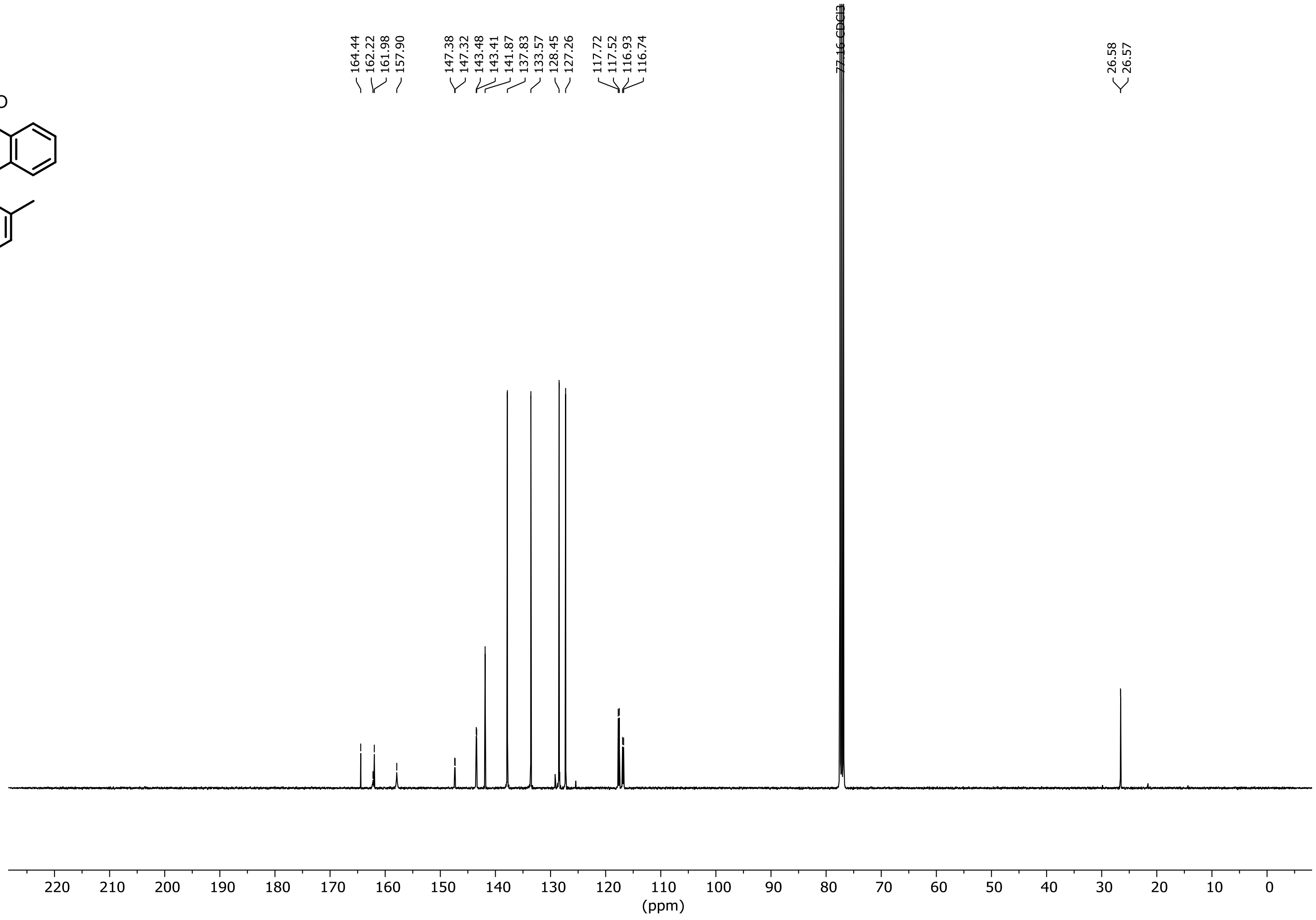

2h – ^{19}F NMR (377 MHz, CDCl$_3$):

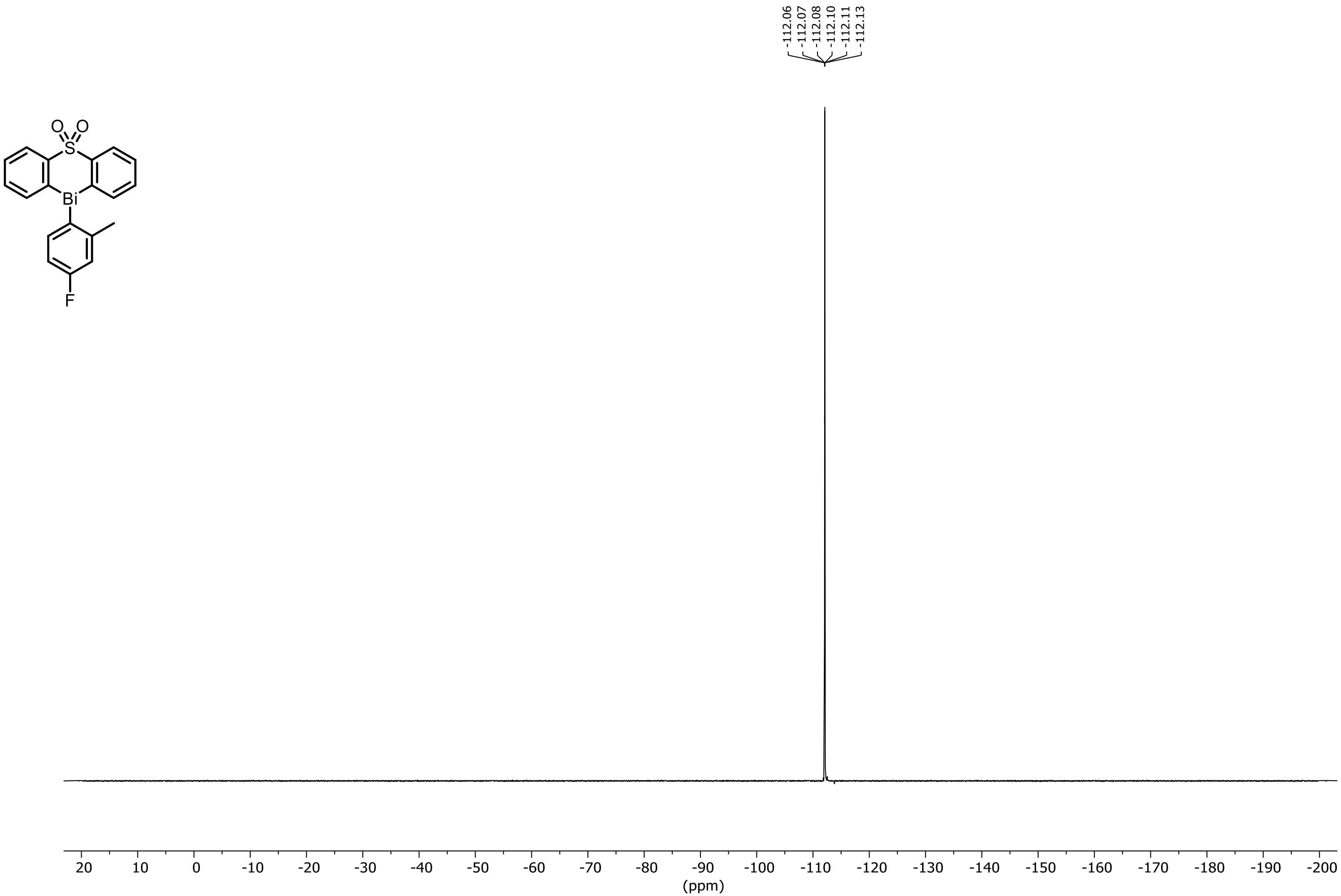

2i – ¹H NMR (400 MHz, CDCl₃):

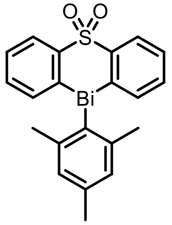

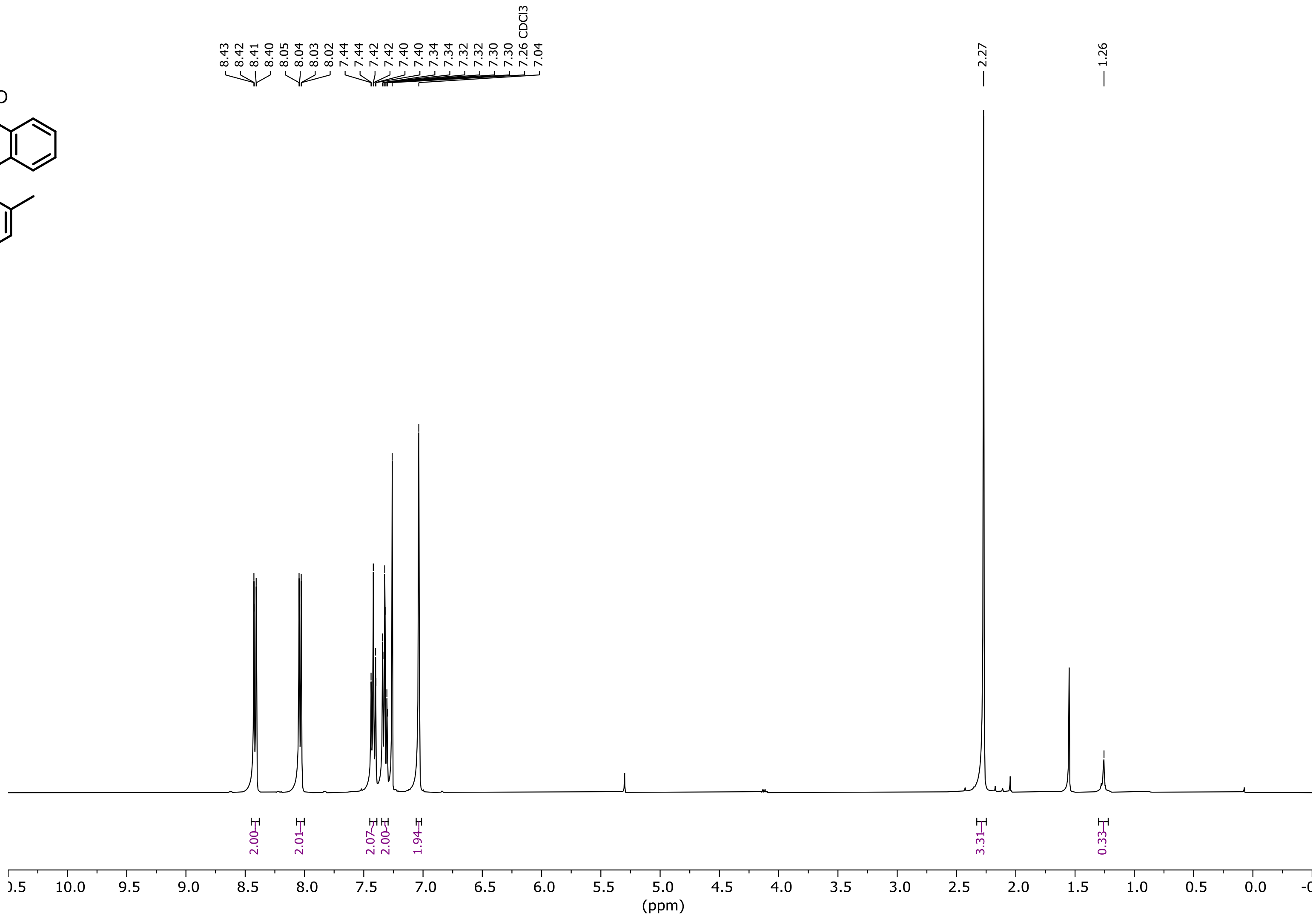

2i – $^{13}C\{^1H\}$ NMR (101 MHz, $CDCl_3$):

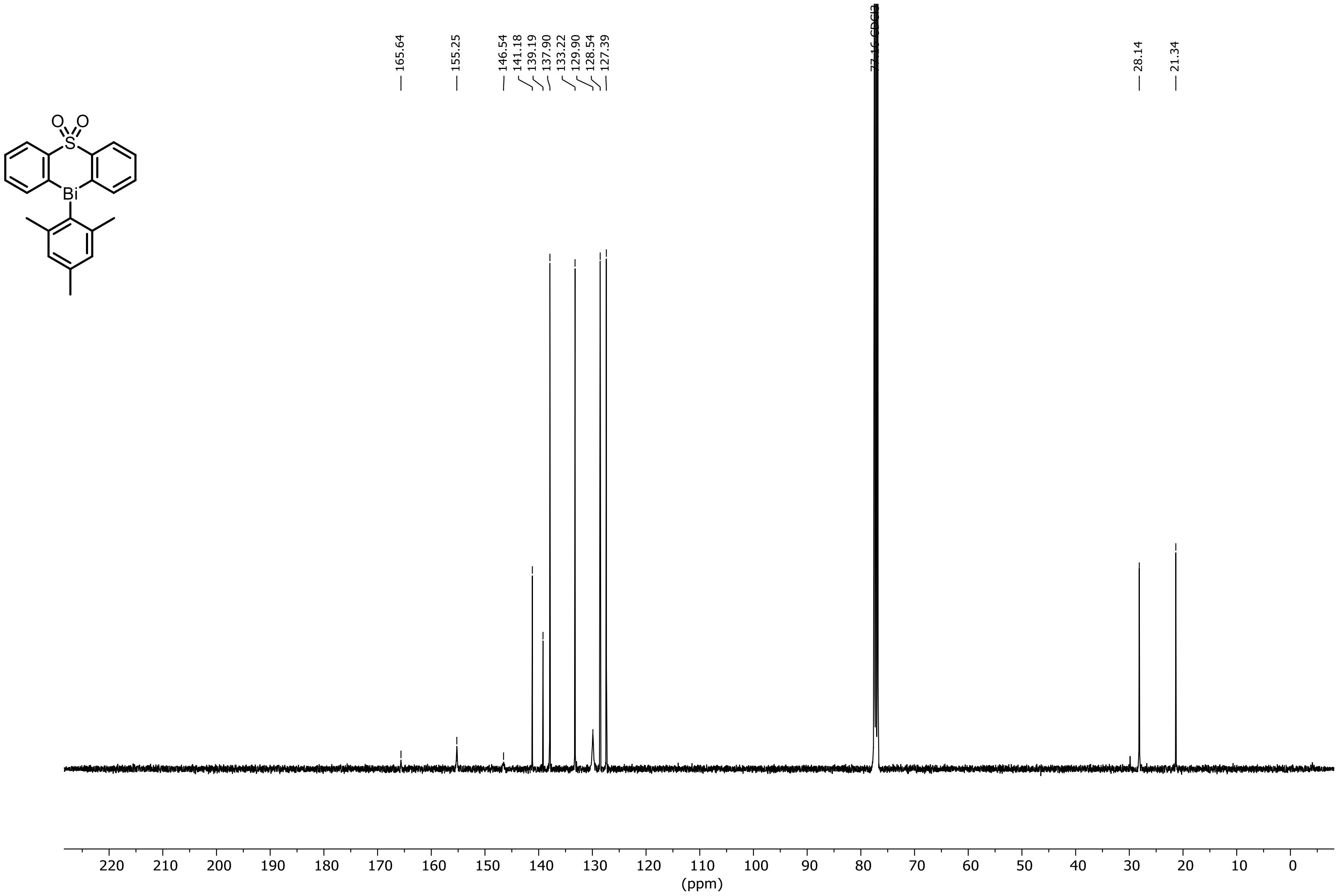

2j – ^{1}H NMR (400 MHz, $CDCl_3$):

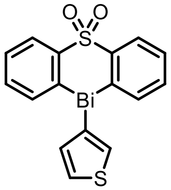

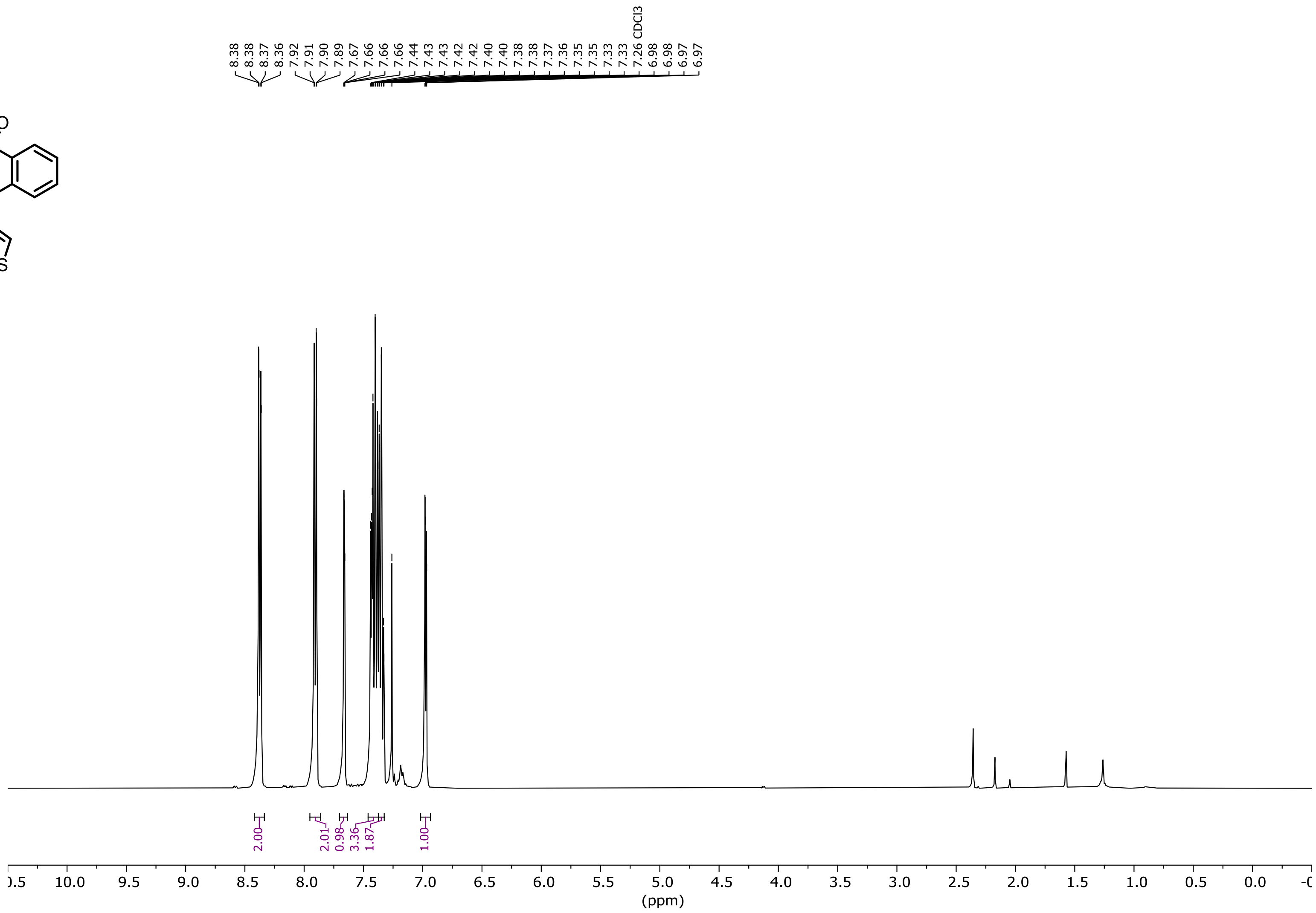

2j – $^{13}C\{^{1}H\}$ NMR (101 MHz, $CDCl_3$):

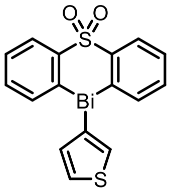

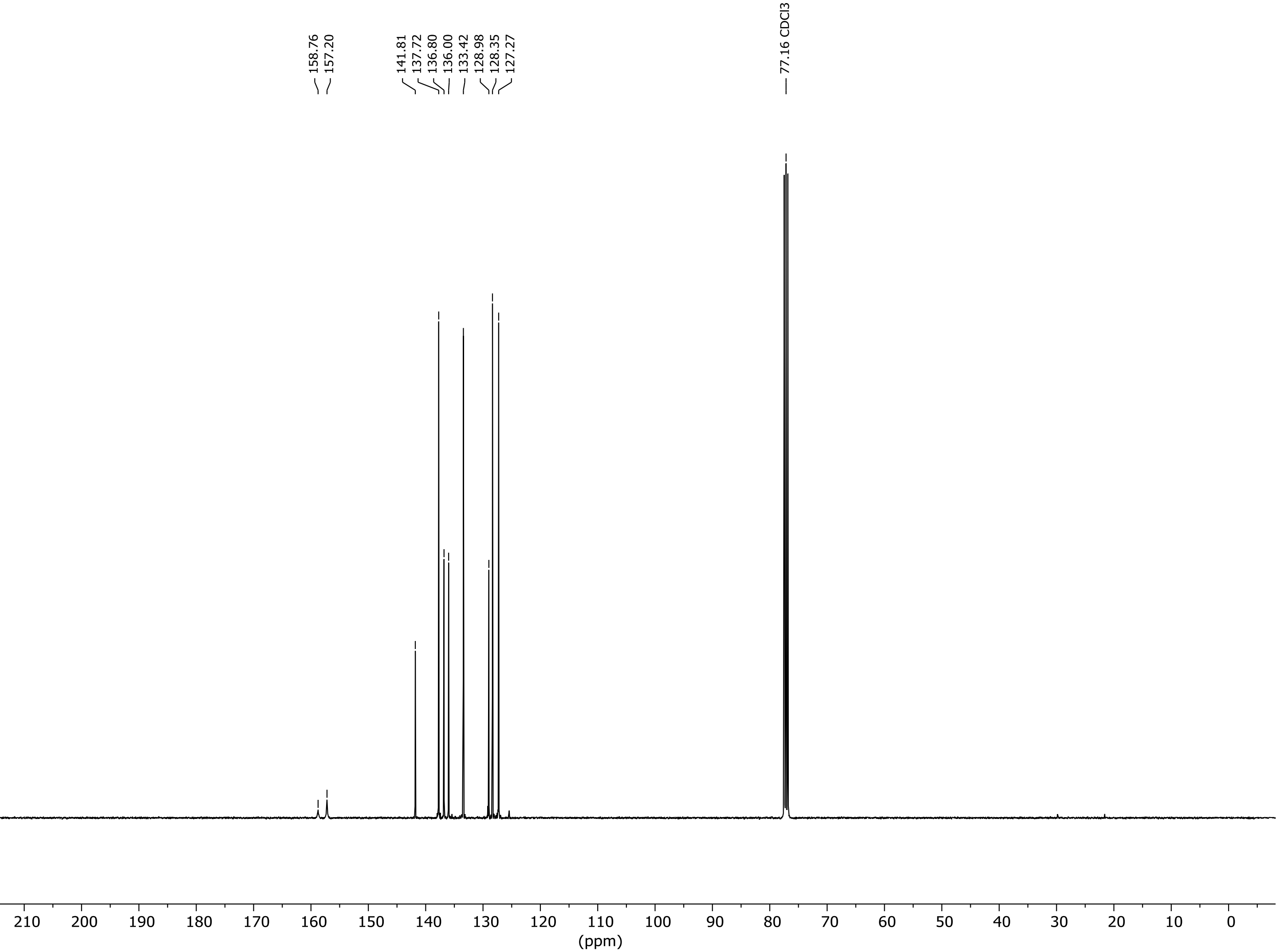

2k – ^{1}H NMR (400 MHz, $CDCl_3$):

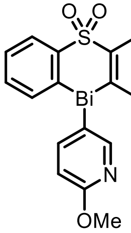

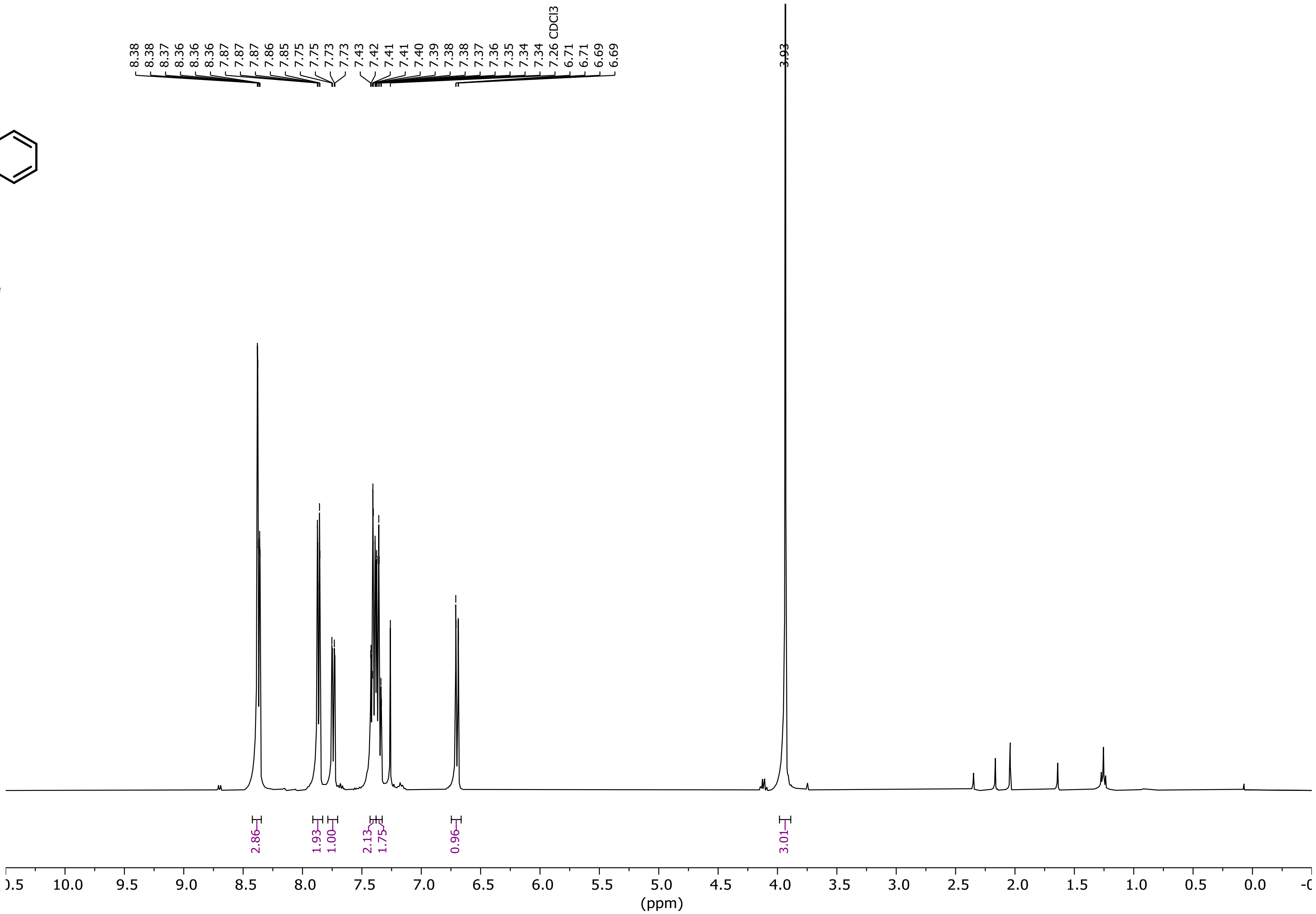

2k – $^{13}C\{^{1}H\}$ NMR (101 MHz, $CDCl_3$):

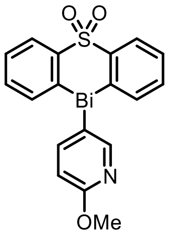

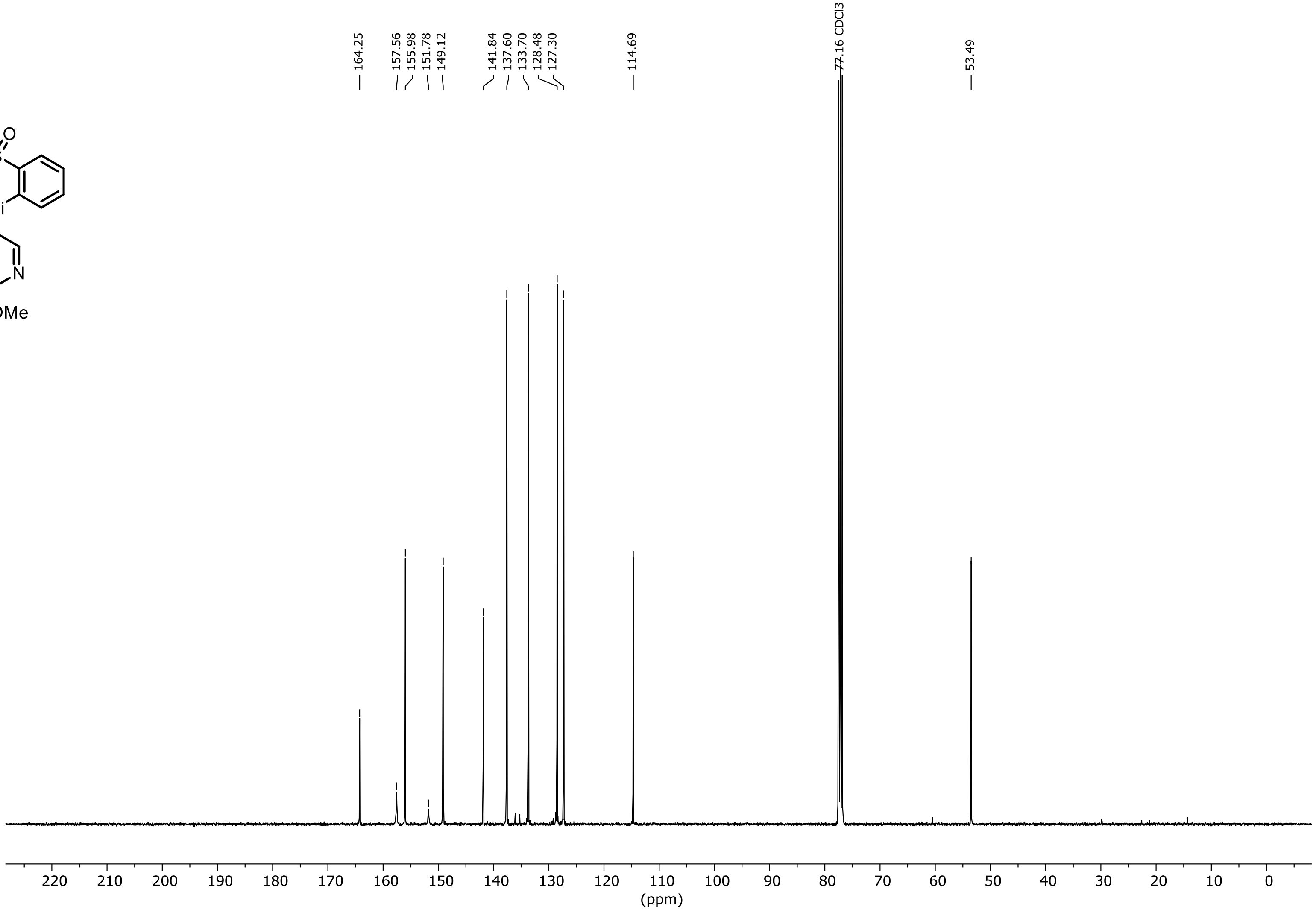

5 – ^{1}H NMR (400 MHz, DMSO-D₆):

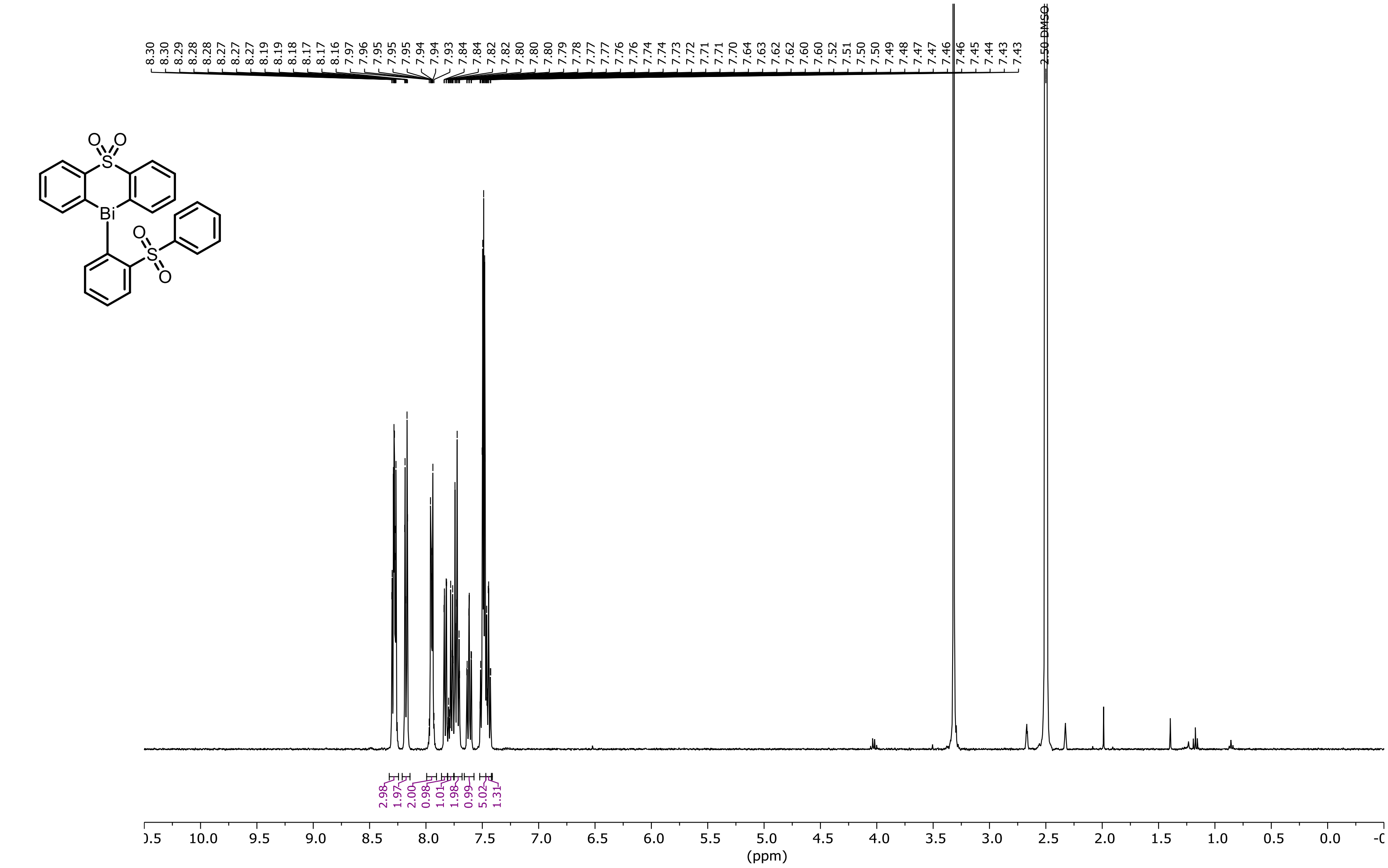

5 – $^{13}C\{^{1}H\}$ NMR (101 MHz, DMSO-D$_6$):

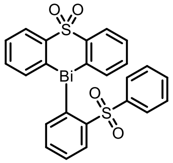

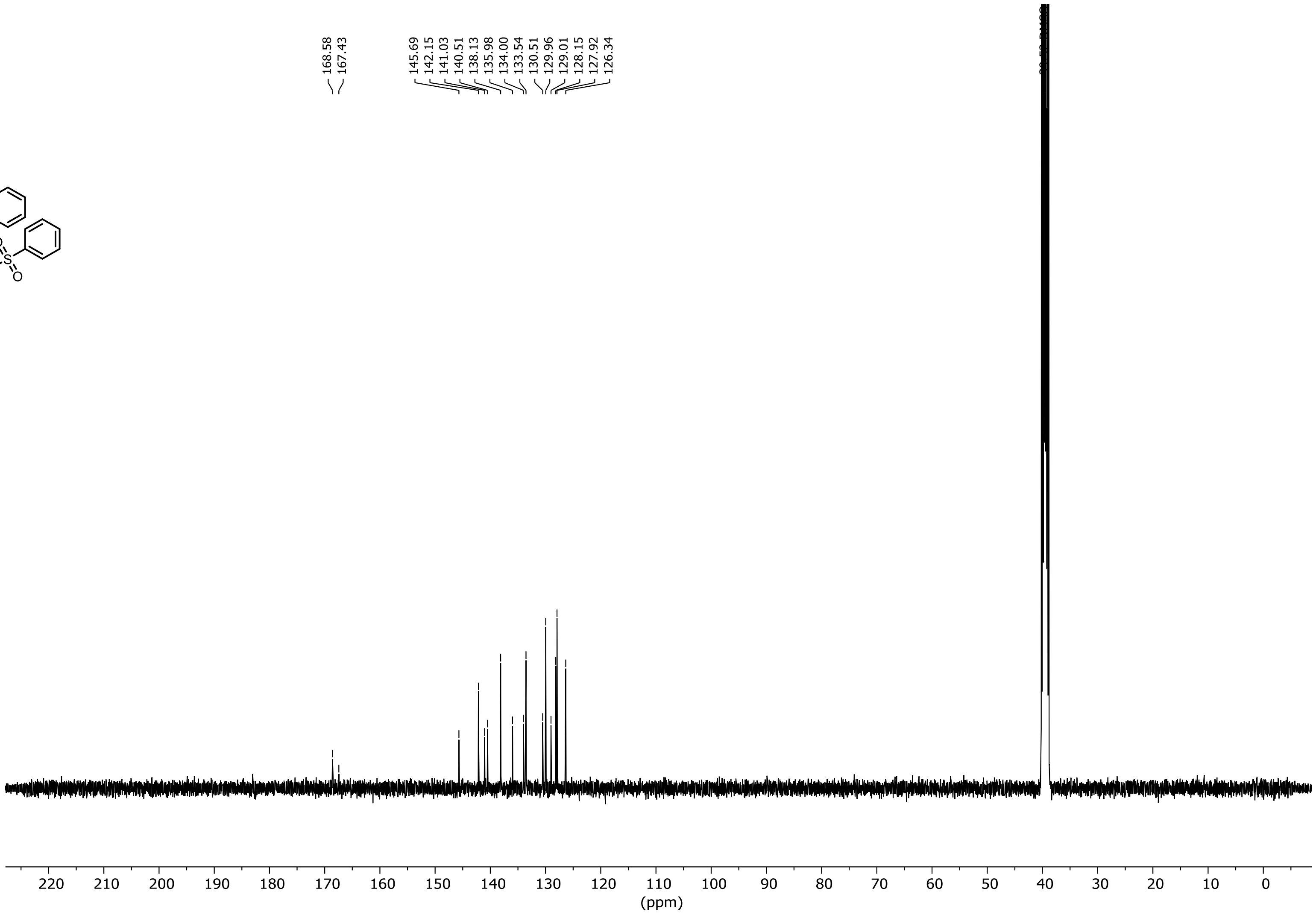

6 – ^{1}H NMR (400 MHz, $CDCl_3$):

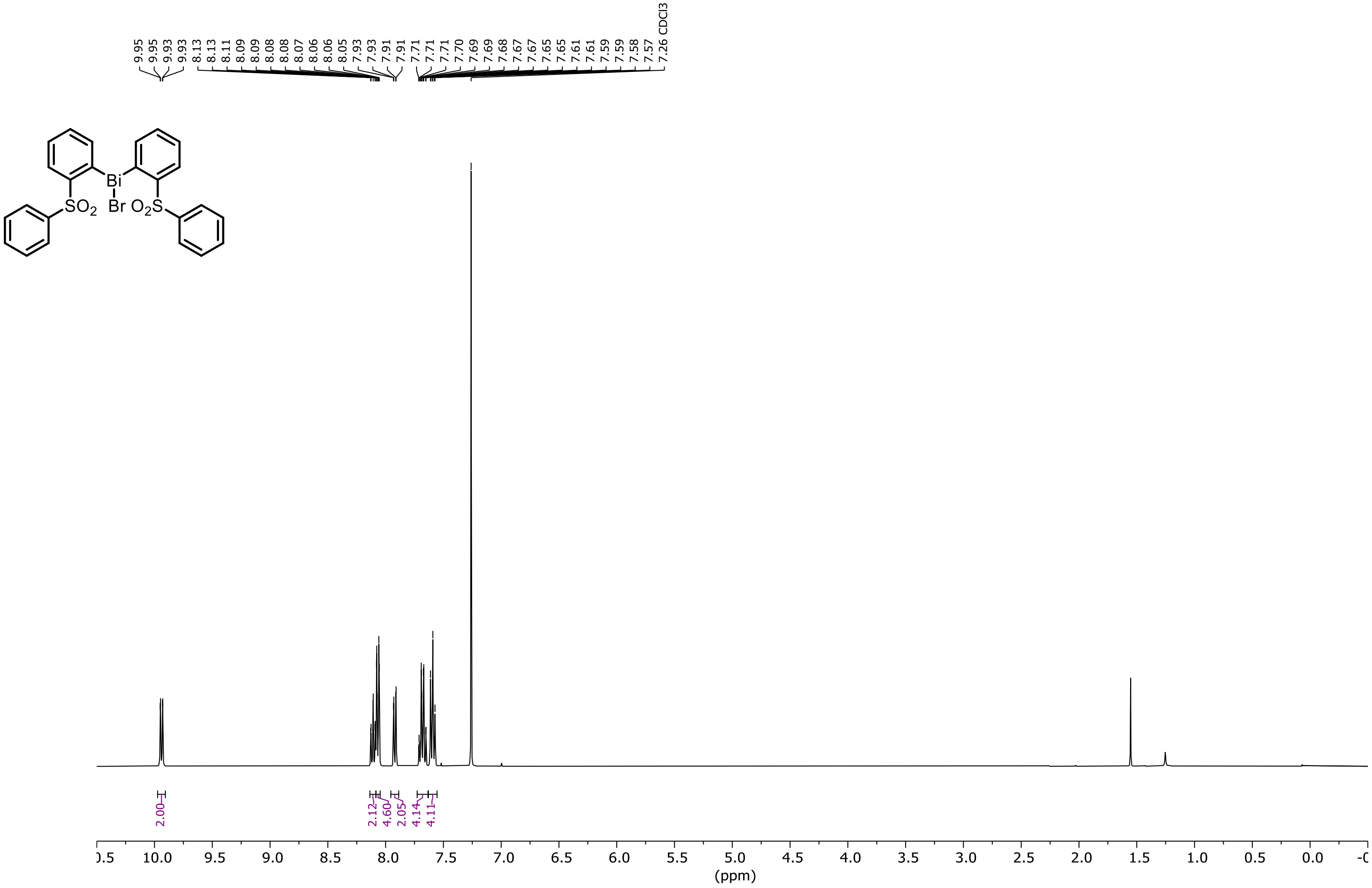

6 – ^{13}C {^{1}H} NMR (101 MHz, CDCl$_3$):

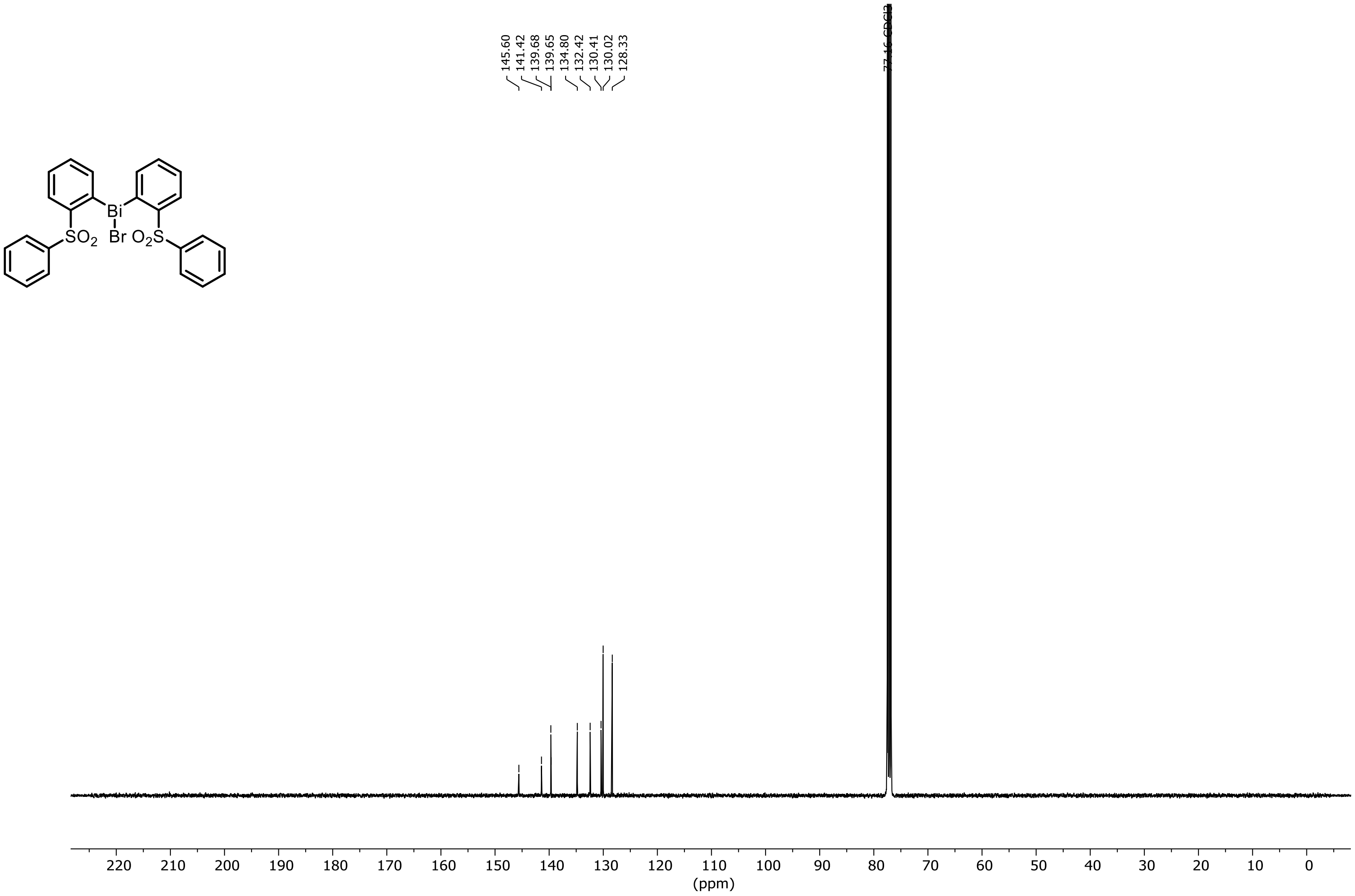

7 – ^{1}H NMR (400 MHz, $CDCl_3$):

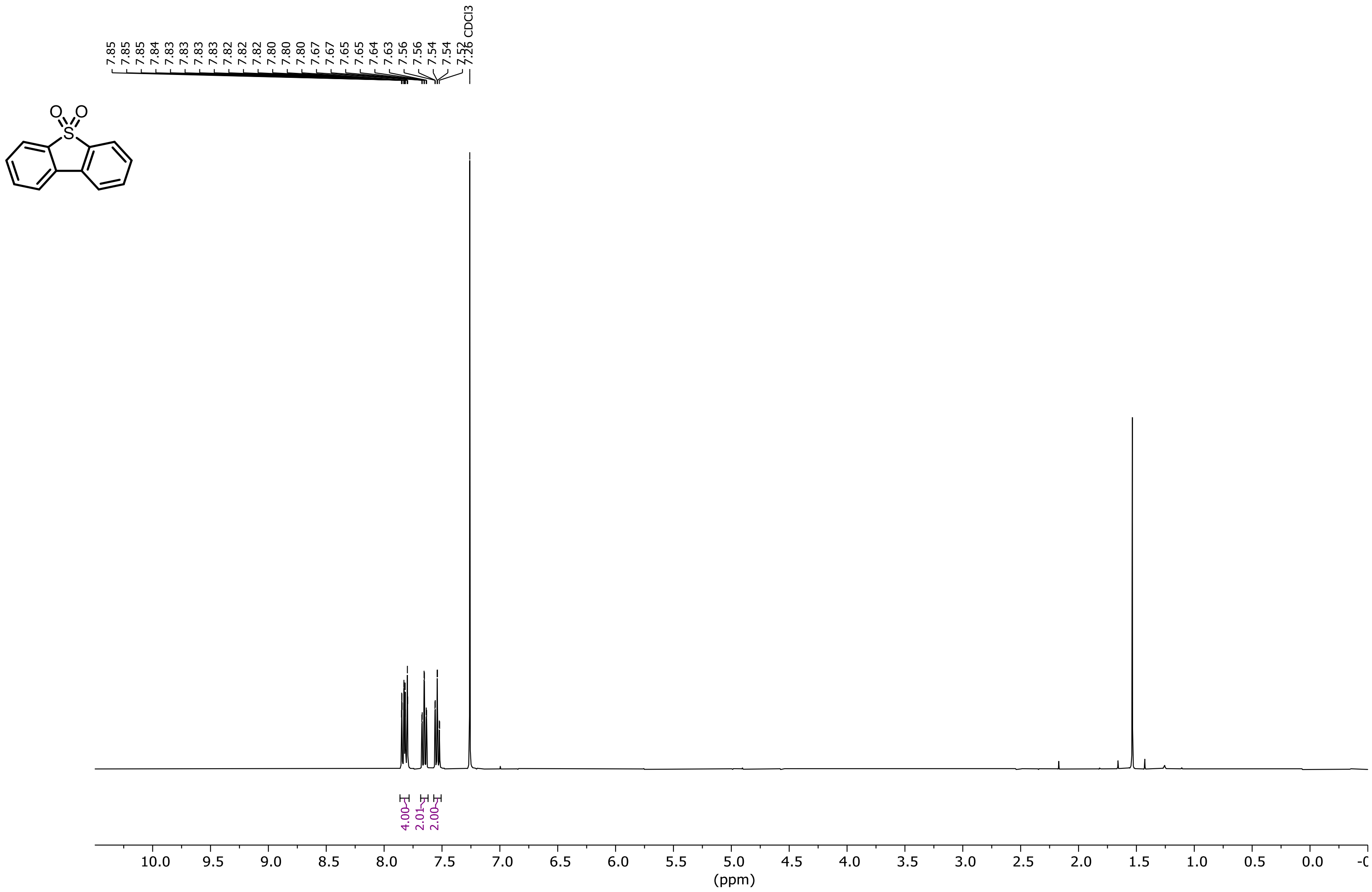

7 – $^{13}C\{^{1}H\}$ NMR (101 MHz, $CDCl_3$):

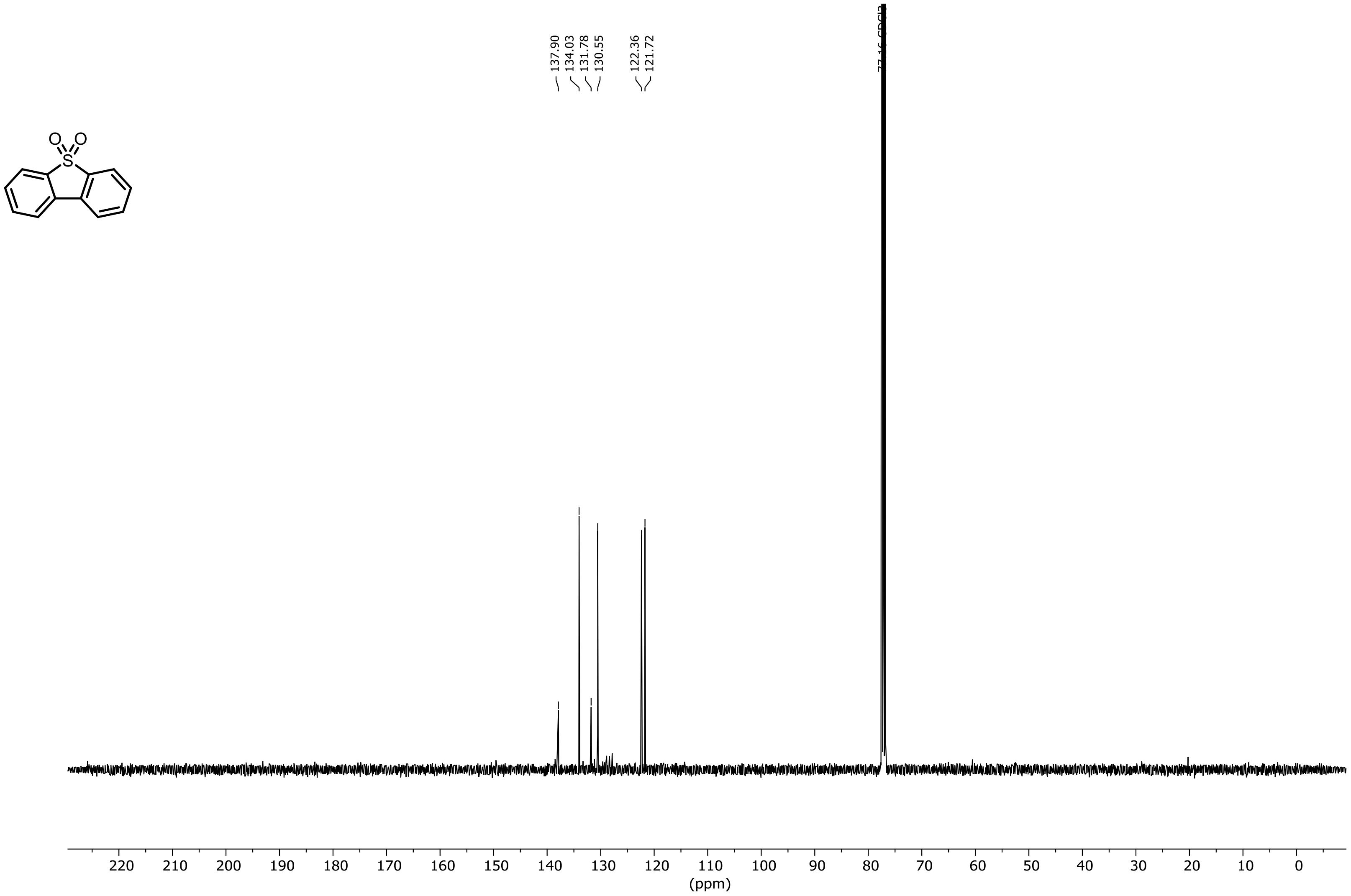

8 – ¹H NMR (400 MHz, CDCl₃):

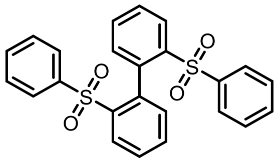

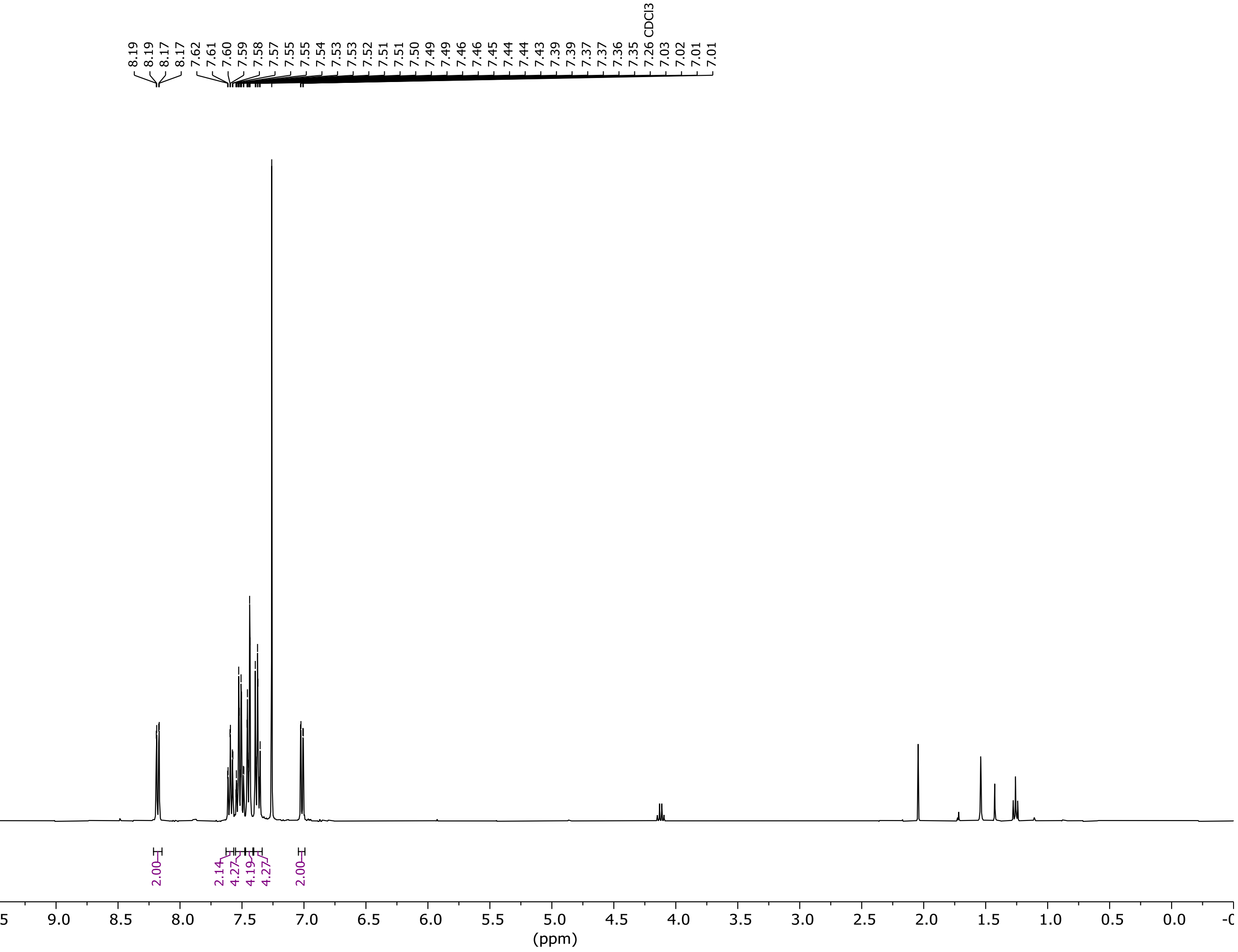

8 – $^{13}C\{^1H\}$ NMR (101 MHz, $CDCl_3$):

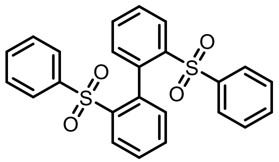

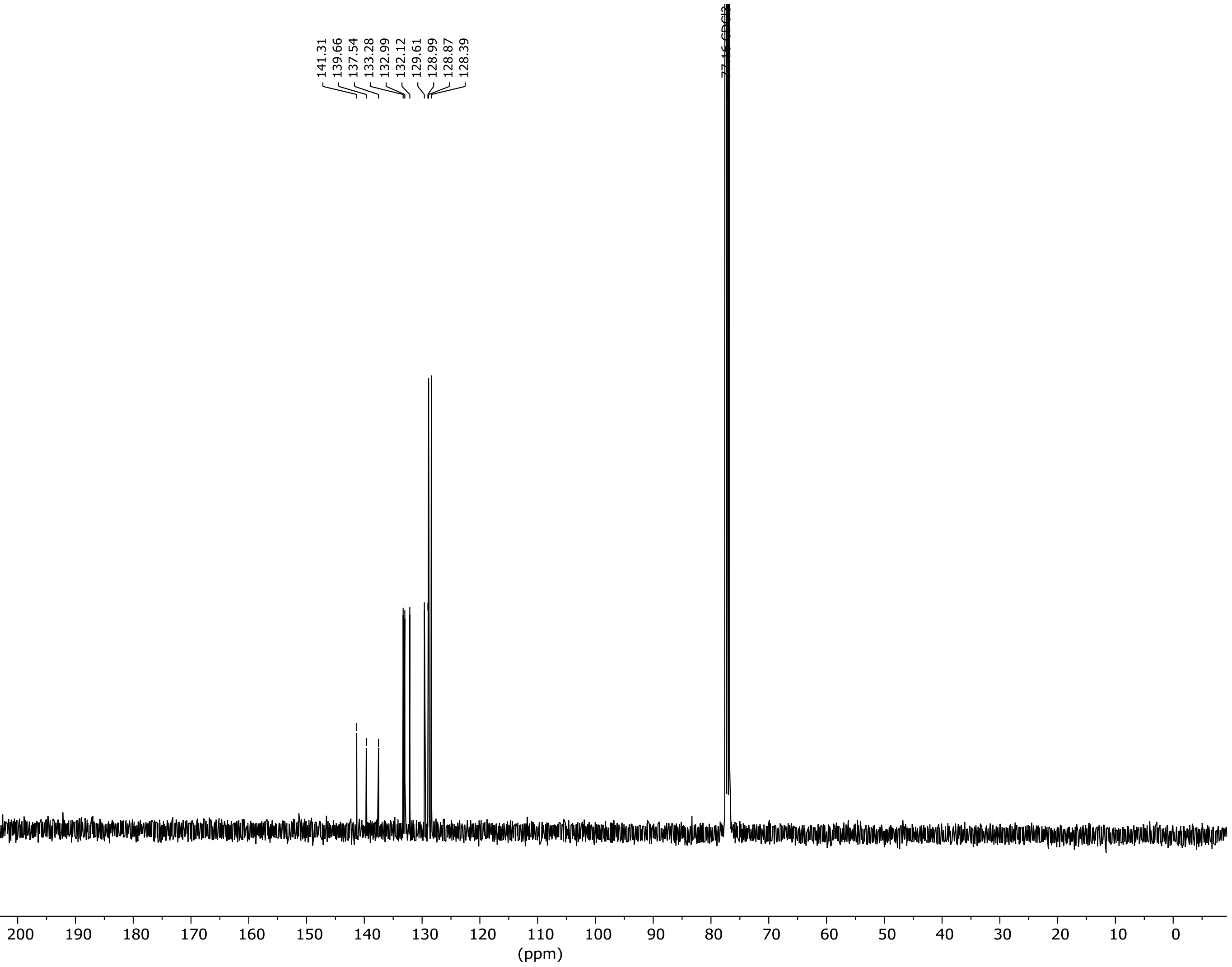

Supplement: Supplementary file 1 — op3c00509_si_001.pdf [file op3c00509_si_001.pdf]
